# Diagnostics for Stochastic Genome-Scale Modeling via Model Slicing and Debugging

**DOI:** 10.1371/journal.pone.0110380

**Published:** 2014-11-04

**Authors:** Kevin J. Tsai, Chuan-Hsiung Chang

**Affiliations:** 1 Bioinformatics Program, Taiwan International Graduate Program, Academia Sinica, Taipei, Taiwan; 2 Institute of Biomedical Informatics, National Yang-Ming University, Taipei, Taiwan; 3 Center for Systems and Synthetic Biology, National Yang-Ming University, Taipei, Taiwan; Koc University, Turkey

## Abstract

Modeling of biological behavior has evolved from simple gene expression plots represented by mathematical equations to genome-scale systems biology networks. However, due to obstacles in complexity and scalability of creating genome-scale models, several biological modelers have turned to programming or scripting languages and away from modeling fundamentals. In doing so, they have traded the ability to have exchangeable, standardized model representation formats, while those that remain true to standardized model representation are faced with challenges in model complexity and analysis. We have developed a model diagnostic methodology inspired by program slicing and debugging and demonstrate the effectiveness of the methodology on a genome-scale metabolic network model published in the BioModels database. The computer-aided identification revealed specific points of interest such as reversibility of reactions, initialization of species amounts, and parameter estimation that improved a candidate cell's adenosine triphosphate production. We then compared the advantages of our methodology over other modeling techniques such as model checking and model reduction. A software application that implements the methodology is available at http://gel.ym.edu.tw/gcs/.

## Introduction

In order to represent various biological mechanisms, modeling in systems biology has taken on several different forms [1]. Some modelers, such as the creators of the Systems Biology Markup Language (SBML), have advocated that modeling maintain a fundamental of standardized representation that can be exchanged, interpreted and simulated by a variety of applications outside of the environment that the model was created in [2]. Other practitioners have advocated that the next evolution of modeling is to move towards software engineering and have turned to programming or scripting tools such as MATLAB, and several object-oriented languages such as Java, C#, and Python [3]–[5]. They have done so in order to take advantage of analysis tools such as run-time debuggers, build automation and other features of common integrated development environments (IDE).

These complexity issues are currently serious obstacles for biological modelers trying to maintain modeling fundamentals [6]–[9]. For example, modelers are often left to question whether a poor simulation is due to the stochastic nature of the simulation or a flaw in the model design. If the flaw is in the model design, then the modeler is left to guess where in the model to begin a model analysis. As models reach higher levels of complexity, the number of computations also increases, creating scalability and performance issues that further burden diagnostics. More generic approaches used in other modeling fields, such as traditional model checking, struggle with compatibility to a biological context due to the scale and stochastic nature of systems biology.

Although there exists efforts at model refinement protocols with tools such as SBMLToolbox and COBRA Toolbox, performing these manual refinement methods can take months to a year [10]–[12]. Our work on a diagnostic application offers algorithms to narrow the scope of areas requiring investigation. The application is built specifically for standardized model representation formats such as SBML and address complexity, scalability, exchangeability and efficiency. The methodology consists of creating an instance of the model in physical memory, mapping core debugging practices from software engineering, and applying computational algorithms developed for a systems biology context. These complementary features allow a modeler to perform focused analysis on specific model mechanics without being convoluted by model complexity.

## Methods

### 2.1 Diagnosis Methodology

#### Reaction graph

**Figure 1:**
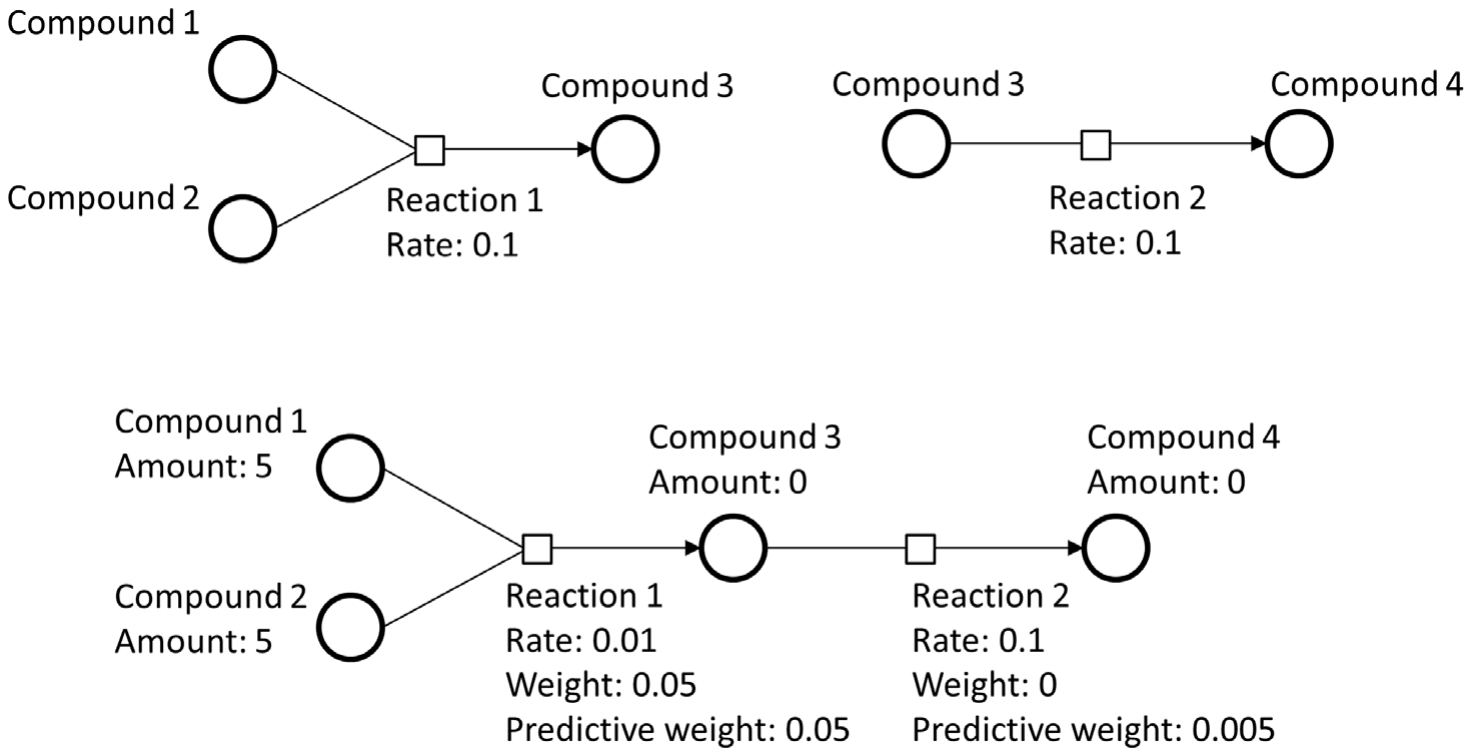
A concept diagram of the reaction graph. In this example, the product for reaction 1 is a reactant for reaction 2. These two reactions can be connected together with reaction 2 designated as a downstream reaction for reaction 1. If the reactants for reaction 1 are available but the reactants for reaction 2 are not, reaction 2 will receive a non-zero predictive weight due to being a downstream reaction of an available reaction. The predictive weight represents a probability that a reaction will occur after the execution of mandatory upstream reactions.

After instantiation of a model, a reaction graph is created to act as a data structure for the creation of model slicing and predictive weights. Upstream connections for a reaction *r* are determined by all reactions that produce the reactants for reaction *r*. Conversely, all downstream connections for a reaction *r* are determined by all reactions whose reactants are produced by reaction *r*. An example of a reaction graph with up and downstream reactions is demonstrated in Figure 1. In order to prevent infinite or repetitive links, a downstream connection will not be added to the same reaction graph twice, which can occur during a network cycle or converging reaction paths. Exceptions are made for up and downstream connections for a list of common cofactors that can be defined by the user.

#### Model slicing

**Figure 2:**
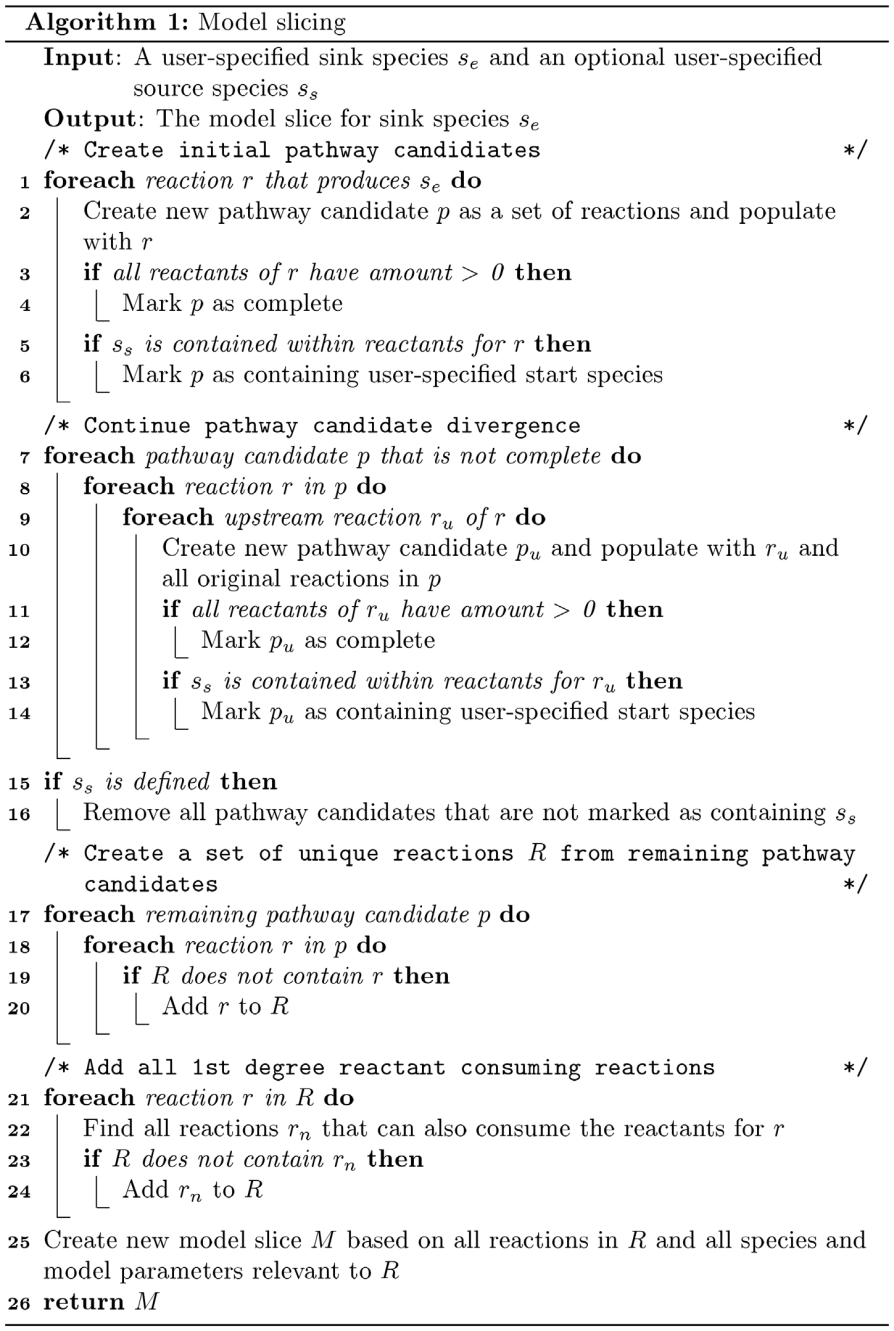
The algorithm for model slicing.

In order to address the scalability issue of genome-scale modeling we have implemented a model slicing technique, which borrows in fundamentals from program slicing, where portions of the model are sliced away to give a much simpler model subset [13]. Slicing is performed by identifying and combining all reaction paths that can create the desired species, each path known as a pathway candidate, into a new abridged model. Only the unique reactions of pathway candidates become part of the slice, with completely redundant candidates left unneeded. The technique complements core debugging methodologies that are more effective as the scope of model information is reduced. Model slicing allows the modeler to view a limited number of reactions, parameters, and species that are relevant and have a dynamic impact on a species of interest. This allows simulation-based analysis to become much more feasible. The algorithm for model slicing is described in Figure 2.

#### Evaluation of model slicing

**Figure 3:**
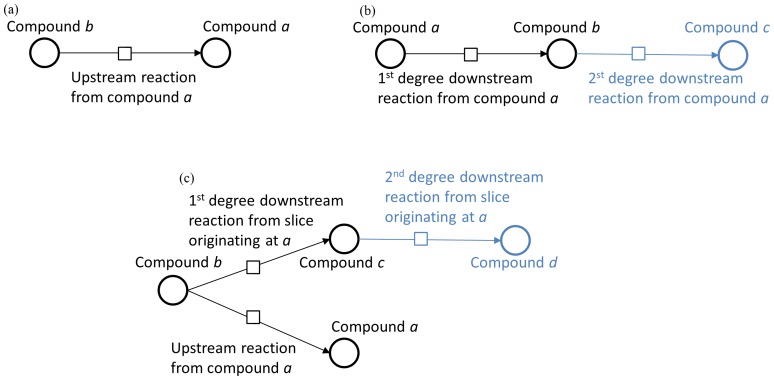
A concept diagram of the model slicing evaluation. In this example we have graphically represented the evaluation of model slicing with simplified upstream, downstream, and combination of upstream/downstream connections as an inductive foundation for more complex examples. The first figure (a) shows the inductive step of upstream connections, where anything upstream of species *a* is included in the slice. The second figure (b) show a 1^st^ and 2^nd^ degree downstream connection, where the 2^nd^ degree downstream connection is removed, however the 1^st^ is kept due to its direct influence on *a*. The third figure (c) represents how slicing treats downstream connections for peer branch species of *a.* In all figures, the light blue color represents the model information cleaved from model slicing with respect to compound *a.*

We used a heuristic justification approach to analyze the relevant information maintained by the model slice and assume all cofactors are readily available. Figure 3 uses simplified examples to demonstrate the logic behind the model slice. The assumption regarding the availability of cofactors is detailed in the discussion.

1. Base: A model slice for a model with a single species *a* will still yield the same model according to the creation of the initial pathway candidates.
2. Inductive step of upstream connections: An added species *b* with a reaction converting *b* to *a* will result in an upstream connection for *a*, yielding a slice of the same composition as the original. All further upstream reactions are similarly handled.
3. Inductive step of downstream connections: If a single species *b* and a new reaction in the downstream direction are added, the model slice will also include the new species and new reaction for only the 1^st^ degree downstream reaction. All 1^st^ degree downstream reactions that contain current model slice species as reactants must also be added to the model slice because they consume the key reactant. When a 2^nd^ degree reaction, e.g. a reaction converting species *b* to a new species *c* is added, the model slicing begins to slice away information irrelevant to the status of species *a*.

The evaluation of model slicing demonstrates how simple networks treat slicing for information upstream and downstream of a target species *a* while maintaining biologically relevant information for the behavior of the target species. Larger networks that are used for biological network modeling are essentially extensions of these base examples and follow the same up and downstream patterns that model slicing recognizes. By following these patterns and scaling up, model slicing aims to extract network behavior that influences a target species while behavior that is not of direct influence to the target is removed, making for a simpler composition that is easier to model relative to a specific species. After correcting model behavior for the slice, the modeler can reintroduce the edited slice to the original model.

#### Forward algorithm-like predictive weights

**Figure 4:**
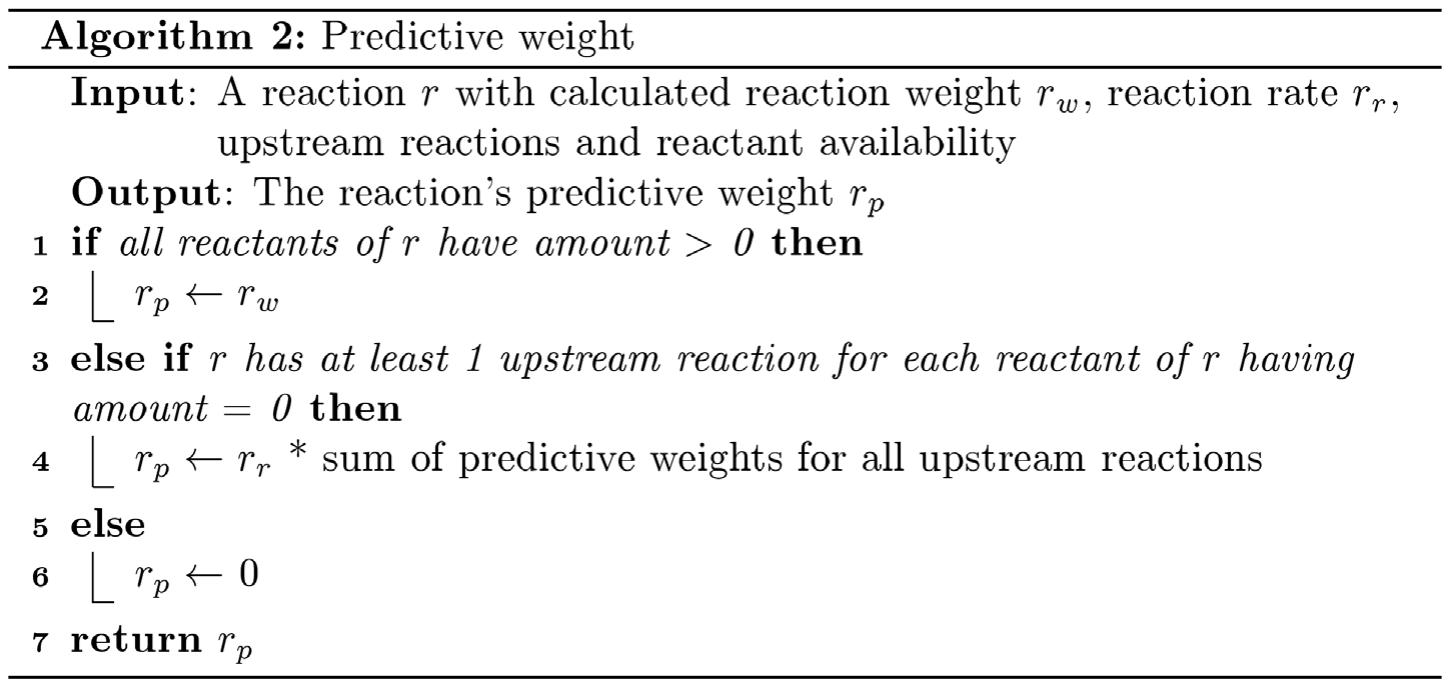
The algorithm for predictive weights.

The probability that a reaction occurs at a given time is determined by the Gillespie algorithm implemented in the simulation engine. The algorithm takes into account reactant availability and the reaction's kinetic law, specified in the model, in order to compute a reaction weight, which is correlated with the probability. In several instances, especially that of long pathway networks, several reactants may not yet be available until the execution of mandatory upstream reactions. However, a calculation is still useful to determine the probability of reaching a certain reaction as the species created may be of central interest to the network. To address this, we have implemented an algorithm based on the forward algorithm for hidden Markov models which calculates a state sequence probability by iterative probability aggregation. The algorithm uses the same reaction graph model slice to calculate the probability of a reaction occurring when none of the reaction's reactants are immediately available. The algorithm looks at the first instance of reaction availability in upstream reactions. This “predictive weight” is calculated dynamically at each simulation time point using the algorithm described in Figure 4.

#### Core model debugging

The methodology of debugging for biochemical organisms consists of core methodology derived from traditional software debugging methods found in common IDEs. The most commonly used features are: allowing a user to specify conditions in which runtime execution of a simulation will halt, observable values of model components at any simulation time point, modifying the data objects and reaction ordering of the model during runtime, and receiving an informative notification when the instruction set reaches an inoperable, non-progressable state.

Being able to control the simulation and having direct access to the parameters in the model can provide insight into faulty model behavior and mechanics [14]. The parameters determine the rates of reactions and the upcoming pattern of the model. After evaluating the status of the model at varying time points, decisions can be made on whether to continue the simulation or make modifications that will affect the following set of instructions.

The debugging application also provides the user with detailed information on simulation “deadlock”. Execution of an instruction can modify a model to an inoperable and non-progressable state and a notification will be produced with the reason for being inoperable. Common reasons are: lack of available reactants in the model, reaction rates of available reactions being too low, or a constraint is set by the model's instruction set that does not allow simulation to advance. The application allows on the fly modification to get out of a deadlock, or allows the user to skip the terminating instruction.

#### Complementary features

There are several complementary pieces of functionality in the debugging application that allow a user to further analyze and improve the accuracy of a systems biology model. A table of all the debugging features in the application along with descriptions and possible usage scenarios can be found in the supporting information.

### 2.2 Simulation

#### Stochastic algorithm

The algorithm implemented in the application's simulation engine is based on Gillespie's exact stochastic simulation algorithm (SSA) for coupled chemical reactions, which is a Monte Carlo procedure for time trajectories of molecular populations [15]. A stochastic algorithm has been found to be more accurate than a deterministic algorithm for smaller systems such as cells because they assume a large number of molecules and no random fluctuations in population values [6].

For stochastic simulation, the 4-step algorithm expects at minimum a core set of species and reactions within the model [16] and is described below:

1. Initialization: Initialize the model with time = 0 and set all species to their initial amounts.
2. Monte Carlo step: Propensity values or reaction weights are calculated based on the reaction rate to determine the probability of the next reaction and the combinatorial evaluation of available reactants. The chosen reaction and time of execution is dependent on a comparison of the reaction weight.
3. Execution: When the next reaction is selected, the time units are increased according to the Monte Carlo step. Each species' populations are changed according to the execution of the reaction.
4. Iterate: The process is repeated starting from the Monte Carlo step.

#### Deterministic algorithm

One of the benefits of using standardized model formats is that they can be interpreted differently by various simulation models [17]. The reactions in our implementation are executed in a stochastic manner, but the methodology can similarly be applied to biochemical models that contain deterministic behavior. Examples of such are triggered events, assignment rules, or rate rules where the simulated equations always result in the same outcome.

### 2.3 Model Source

The BioModels database, hosted by the European Bioinformatics Institute, has a category of whole-cell metabolic network models available for download. These contain both computationally translated models such as those done by the path2models project and manually created efforts [18]. One of which, an effort by Palsson's group at the University of California, San Diego, BioModels ID MODEL1108160000, reconstructed a model to represent the entire metabolic network of an *E. coli* cell [19] using a reconstruction protocol originally published in Nature Protocols [11]. While the latest model is the most comprehensive model to date it still lacks, due to the intended flexibility of the model, several required pieces of information and is not available for simulation in its initial state.

## Results

Our goal was to demonstrate computer-aided identification of modifications required to boot up the energy generating mechanics of the model via promotion of adenosine triphosphate (ATP) development and observe their effect on the glycolysis pathway. We chose ATP production since it is a well-known, important and complex cell function that a modeler may be interested in reproducing [20]. Application of the debugging methodology is not focused on correcting issues that are hard to solve, but proving its effectiveness in computer-aided identification of issues that may be hard to find. Although there are many uses and approaches possible with our diagnostic methodology, our approach for this exercise was as follows:

1. Create a model slice specific to ATP production
2. Identify and refine the mechanics required to produce ATP
3. Insert the modified model slice back into the original model to confirm a transfer of simulation behavior

Reaction nomenclature such as R_PGM and R_PGK is used to identify specific metabolic reactions. More information regarding the reaction details can be found in the corresponding reference [19] and the supporting information.

### 3.1 Model Slicing of Genome-Scale Models

#### Initial pathway candidates

**Figure 5:**
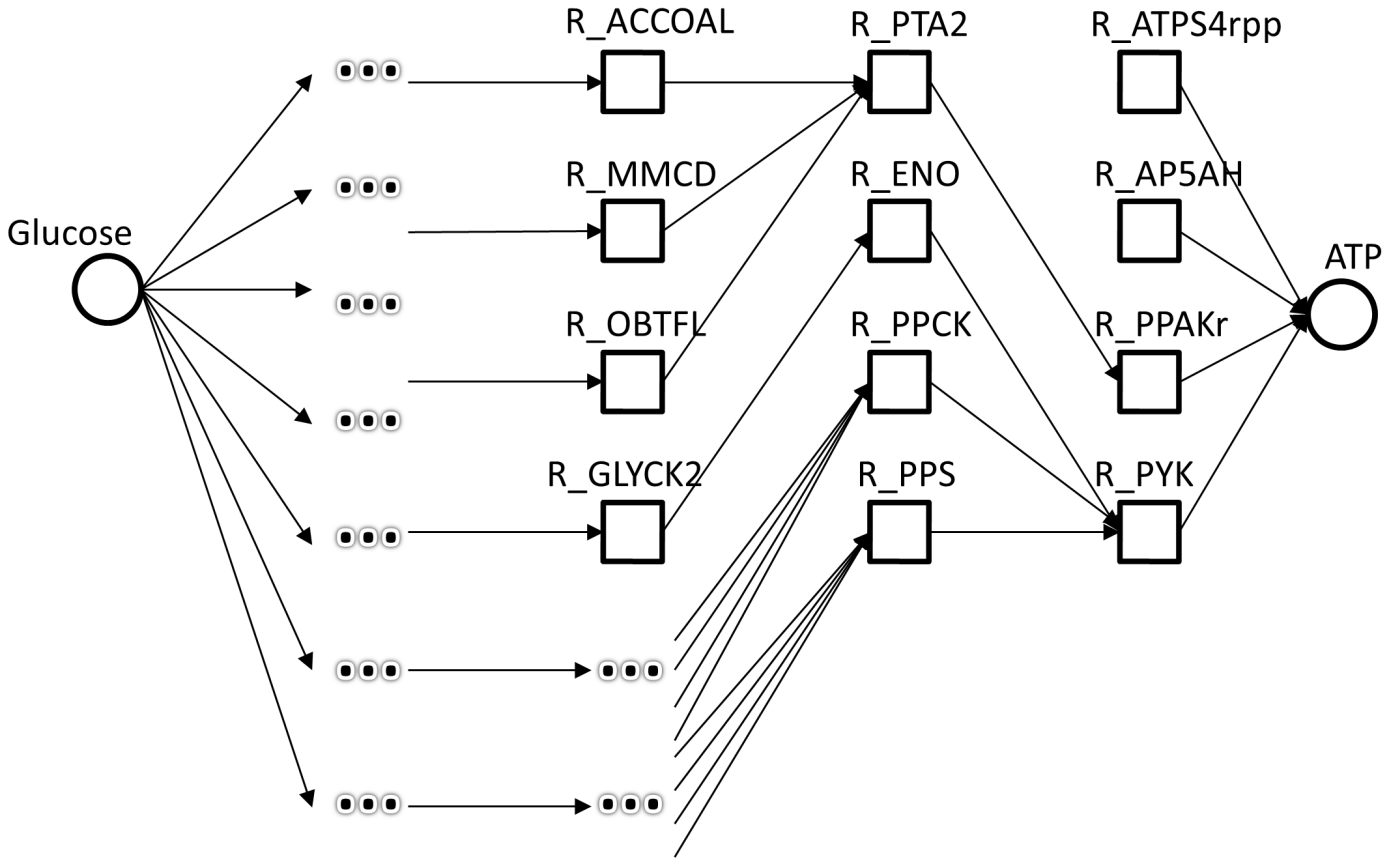
An excerpt of pathway candidates generated during an initial model slicing of the *E. coli* model using ATP as the sink species and glucose as a source. R_PYK, the pyruvate and ATP generating reaction, was correctly connected to R_ENO, which produces phosphoenolpyruvate, during creation of the reaction graph. However, R_ENO only contained one upstream reaction, R_GLYCK, due to the incorrect assignment of R_PGM's reversibility. This reaction is normally upstream to R_ENO in the glycolysis pathway. The reactants in R_ENO are not considered dead-end metabolites that would normally be found in gap analysis as they can still be produced by R_GLYCK.

Using the model slicing algorithm, we created an initial slice of the model which yielded 7,061 unique potential pathway candidates that could generate ATP. Evaluation of the pathway candidates showed missing connections in the reaction graph for expected glycolysis reactions, shown in Figure 5. This identification, which would not have been discovered via gap analysis of dead-end metabolites, led to the discovery of missing reversibility assignments in two key glycolysis reactions, R_PGM and R_PGK. Although reversibility is assumed to be true by default for the version of SBML that the model was encoded in, by the latest SBML standards the model does not meet that minimum requirement. We resolved the issue by specifying the correct reversibility for the reactions and regenerated a new set of pathway candidates that contained the expected pathway.

#### Selection of source metabolites

After correcting the model reversibility of select reactions we generated a model slice consisting of 8,240 unique pathway candidates, 1,323 unique reactions and 1,160 unique species. Using model reaction count as a measure for model complexity, this yielded less than a 50% reduction in model complexity from the original model. We then assigned glucose as the primary source for ATP generation to reduce the scope of possible sugar sources. The comparison of original model and model slice are represented in Table 1.

**Table 1:** Comparison of complexity reduction when specifying different source and sinks for the model slice.

Model	Reactions	Species
Original model	2,583	1,807
Pyruvate slice	1,755	1,386
ATP slice	1,323	1,160
ATP slice with glucose as a source	178	304

By setting pyruvate as the sink, the original model with 2,583 reactions and 1,807 species was reduced to a model slice with 1,755 reactions and 1,386 species, a very small reduction as many reactions can lead to pyruvate and several upstream paths can lead to those reactions. Assigning ATP as a source yielded a model slice with 1,323 unique reactions and 1,160 unique species, also a small reduction due to the number of energy sources used to generate ATP. By specifying glucose as the source, we achieved a slice of 178 reactions and 304 species, over a 93% reduction in model complexity.

### 3.2 Analysis of Behavioral Mechanics

**Figure 6:**
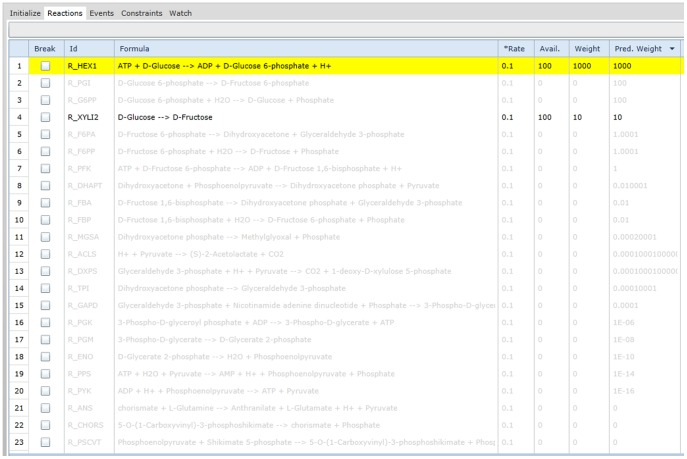
A list of reactions as seen in the main workspace of the diagnostic application. The highlighted reaction represents the reaction that the Gillespie algorithm has chosen to execute based on reaction weight. The predictive weight column informs the user of the normalized probability of the reaction taking place even if the reactants are not directly available. For example, R_PYK, has a zero reaction weight due to the lack of phosphoenolpyruvate but a predictive weight of 10^−5^. This value is a result of the availability of glucose and the upstream R_HEX1 reaction.

In order to run an initial simulation of the model we needed to populate the model with an initial amount of species and non-zero reaction kinetic law values, two pieces of information not in the original model. We first set all reactions to an arbitrary 0.1 kinetic law value with the intention of refining the species amounts first. With non-zero kinetic law values we were able to calculate reactions weights and use predictive weights to determine species that would have an effect on the eventual production of ATP, even if they were not related to reactions that directly produced ATP. Each time a new metabolite was produced, the predictive weight for all reactions in the model slice were recalculated. A screenshot of the diagnostic application's reaction workspace with reaction and predictive weights is seen in Figure 6.

**Figure 7:**
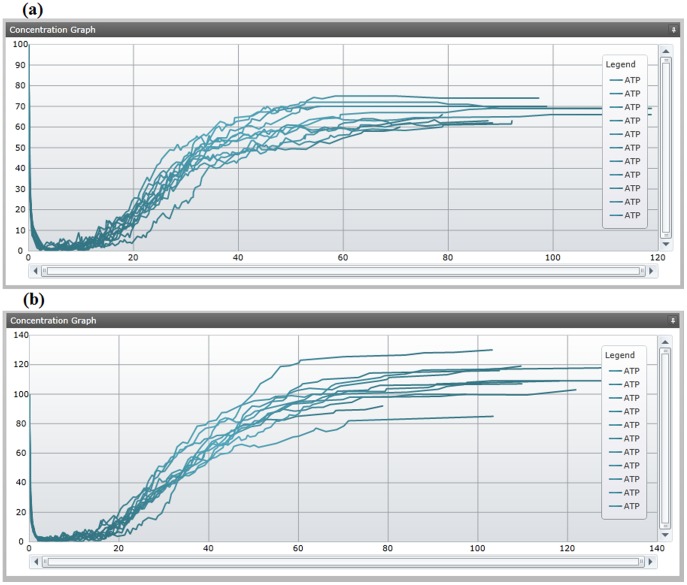
Concentration plots for ATP with initial metabolites and varying reaction kinetic law values. We repeated each simulation 11 times to generate a sample of simulation variability, with some simulations reaching a deadlock state before others. The concentration plot (a) shows the level of ATP, initialized at 100 units, throughout each simulation with arbitrary 0.1 reaction kinetic law values. ATP is consistently consumed towards the start of simulation and slowly builds back up to plateau at an amount ranging from 60 to 80. The exact number of units produced for each species for the model slice is available in the application's watch panel. We then used the diagnostics application to adjust kinetic law values according to reaction weights at simulation breakpoints which allowed the simulation to bypass reaction bottlenecks (b).

Although the applications of predictive weights can go far beyond testing of initial species, we were able to test the influence of a minimum set of initial species based on their influence on ATP generating reactions. We then added the cofactors ATP, ADP, phosphate and NAD due to their positive influence on ATP generation and simulated our new model with the initial species amounts using the application's multiple simulation feature. The feature overlays concentration plots for select species in order to capture more informative insight as to the possible fluctuation due to stochastic behavior. Each simulation demonstrated ATP growth and has been plotted in Figure 7.

### 3.3 Parameter Estimation via Core Debugging

After setting the required initial amounts for a glycolysis pathway we began to refine the efficiency of the pathway's kinetic law values. From the application's watch panel we observed a drop-off in production of 2-phosphoglycerate, which is largely dependent on the execution of the R_PGM reaction, a reaction that turns 3-phosphoglycerate to 2-phosphoglycerate. Once we identified 2-phosphoglycerate as a bottleneck metabolite, we set breakpoints at all 3-phosphoglycerate consuming reactions to determine the simulation mechanic when 3-phosphoglycerate becomes available. Aside from R_PGM, the only other reaction in our model slice was R_PGCD, a reaction that turns 3-phosphoglycerate to 3-phosphohydroxypyruvate.

**Figure 8:**
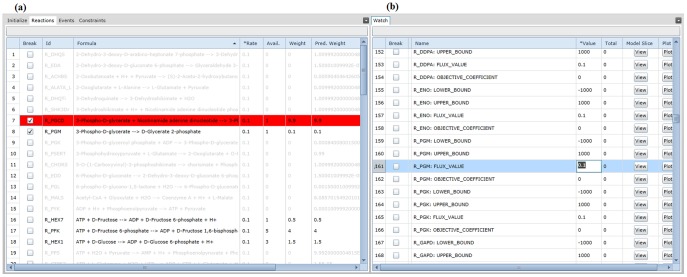
Refining simulation behavior with breakpoints and the watch panel. The reaction list (a) represents the initialized instructions of the model, with each reaction offering a breakpoint or conditional breakpoint to pause the simulation when specified criteria are met. Breakpoints can also be set by the application based on outliers of a multiple simulation. The watch panel for the model (b) shows current amounts and total production amounts for parameters, species and compartments.

Each time 3-phosphoglycerate became available, we compared the reaction weight of R_PGCD with R_PGM at the breakpoint. The weight for R_PGCD was exponentially larger due to the Gillespie algorithm identifying multiple combinations of available reactants for R_PGCD, which also uses NAD, whereas R_PGM only uses 3-phosphoglycerate. We then used the watch panel to adjust the kinetic law value for R_PGM to raise the weight to be more relative to the R_PGCD weight observed during breakpoints. Alternatively, lowering the kinetic law values for R_PGCD could generate a similar effect. A screenshot of the diagnostic application during breakpoint usage in this scenario is demonstrated in Figure 8.

### 3.4 Incorporation into the Whole-Cell Model

The motivation for model slicing was not only to reduce the scope of irrelevant information so that it could be easily modified but to put the modifications back into the whole cell and transfer similar behavior to the original. Glycolysis is an exceptional pathway in that it requires ATP before it can generate ATP. The reaction graph used to create the model slice does not make upstream and downstream connections with cofactors such as ATP for reasons detailed in the discussion. Therefore, as we created pathway candidates for the model slice, the slice skipped the reactions that consume ATP and the downstream associations of those reactions never connected.

**Figure 9:**
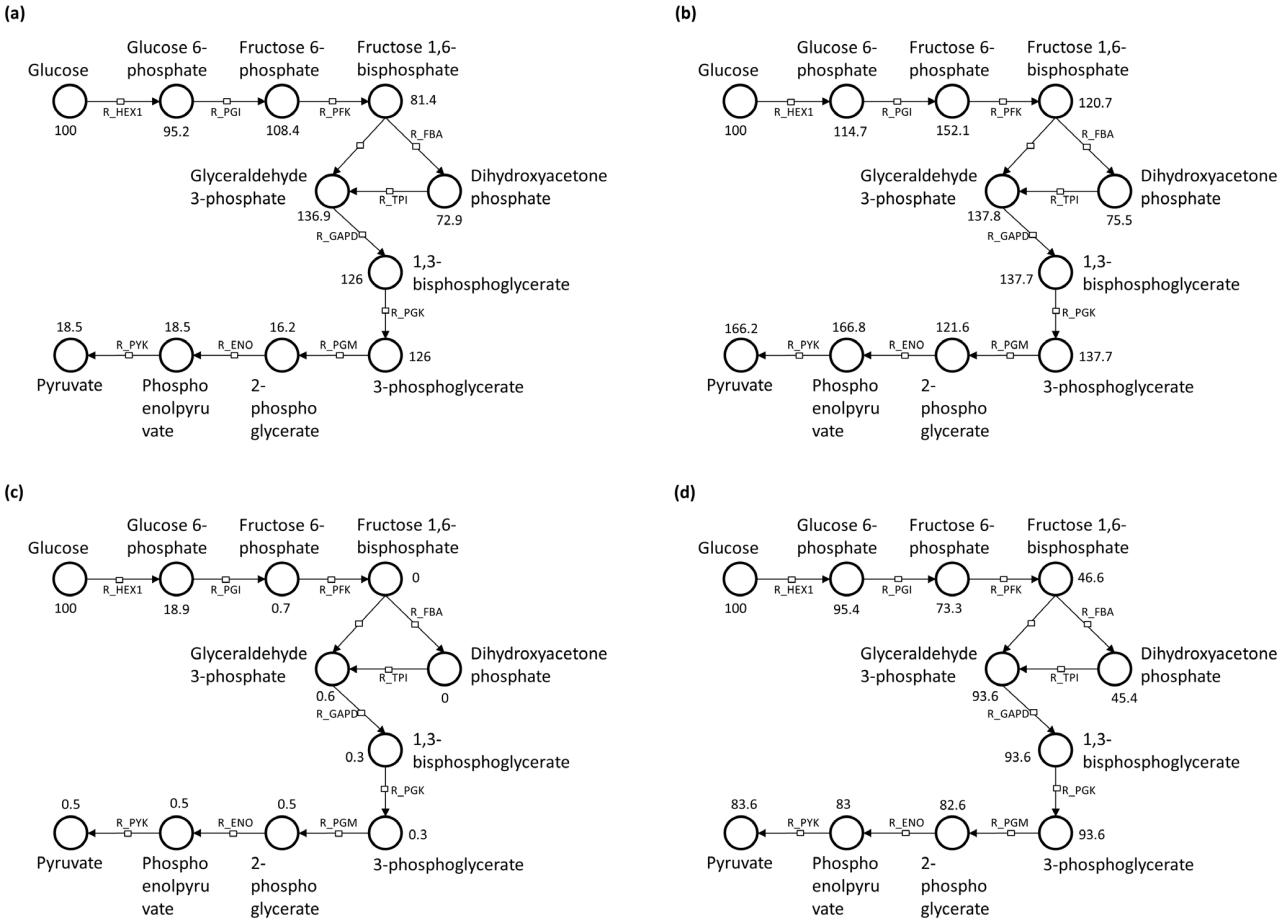
Various network graphs created from production counts in the application's watch panel. The production counts were averaged over multiple simulations. In the initial slice simulation with arbitrary reaction kinetic law values (a), 100 glucose units conserve semi-efficiently through the glycolysis pathway until the production of 2-phosphoglycerate, which only produces 16.2 units on average. The representation in (b) shows the results after the adjustment of the R_PGM reaction. Production amount of downstream species can sometimes be greater than the production of their upstream counterparts due to other species and reactions not listed in the glycolysis pathway that are nonetheless part of the slice. These can be cyclic paths that cause high production counts for those specific intermediates but do not cause a high overall amount. The initial simulation of the whole model with the adjusted slice (c) demonstrated glycolysis bottlenecks towards the initial ATP consuming reactions, however, after adjusting for competing ATP consuming reactions that were not incorporated into the reaction graph we were able to observe adequate production (d). The debugging information for the network representations were obtained from the application's watch panel and are not typically observable in traditional concentration plots.

We were able to observe a lack of ATP due to the low production of initial glycolysis species in the application's watch panel. Since ATP is required for ATP production, we lowered the kinetic law values of ATP consuming reactions. We identified 4 ATP consuming reactions (R_AP4AS, R_NADK, R_PPKr, R_PPK2r) that were in the original model and not accounted for in the model slice. These reactions could be executed with the initial reactants of the model slice during predictive weight testing. The results in species production for all model simulations are represented in Figure 9.

## Discussion

### 4.1 Debugging Compatibility in a Biological Context

While debugging's effectiveness in software and electrical engineering has been clearly defined and practiced for over 60 years, debugging fundamentals have not yet been mapped to a standardized systems biology modeling context. Often times, in-depth knowledge of a model's mechanics can bypass the use of a debugger, such as immediately checking glycolysis reactions and maxing out their kinetic law amounts without an analysis. However, that knowledge will not always be available, especially for less well-defined cell functionality and with models that are constantly exchanged. With the trend that model complexity has taken over the past years, the idea of mapping a scalable debugging practice from software and electrical engineering to standardized biological modeling is new, yet reasonable given the associations systems and synthetic biology has drawn from engineering. However, there are still some important caveats that need to be addressed.

The stochastic behavior of common simulation algorithms cause instructions to be executed in random order. In software, the code is normally executed in a constant order. Reactions, however, are analogous to multi-threaded processes. Each reaction belongs to its own thread and the connection pool decides which thread to execute. Instances like that are uncommon in most software applications, however, in some pathway situations, the occurrence or magnitude of occurrence of reactions will have an orderly pattern as downstream reactions will not occur without proper upstream reactions. In such cases, breakpoints can also be useful in determining when the stochastic execution of reactions has reached certain bottlenecks in a pathway.

Similarly, in some designs, for example the classic repressilator design, single reactions are very insignificant in respect to the grand pattern of a model's behavior. The same reactions are repeated and they need to be repeated several times in order to generate the underlying pattern. In software, each line can be very distinct and the impact can be very powerful, yet software can also contain loops in which several iterations need to be run before the software demonstrates a noticeable change in behavior. In cases like these, using the debugging method's step by step approach would not be a fruitful exercise, but a similar scenario is equally inefficient in a software debugger.

### 4.2 Comparison of Analytical Approaches

Traditional model checking is an expensive process that aims to exhaustively explore all possible behaviors of a model [21] and becomes unrealistic when applied to the random behavior of stochastic models [22]. In recent years there have been several statistical approaches that use Bayesian approaches or probabilistic models to accommodate for the stochastic behavior. These use mathematical approximations and are recommended to be used with other approaches based on simulation runtime [23] for more accurate results.

As systems become more complex, the idea that simulation defects can appear that are undetectable by compile time model checking will become more evident. Attempts have been made to create simulation-based model checking [21] but even for relatively minimal models, applying model checking's fundamentals can require over 10,000 simulations. Computation time for this type of checking can become improbable when a complex model simulation can reach 10 hours [24]. The diagnostic methodology allows analysis at specific problem areas and debugging has been proven to be more scalable in its use with software engineering.

Several modeling tools have compile time integrity checks for a network where defects such as incorrect formats or contradicting interactions will be analyzed [25], [26]. Those checks are based on the model and the information in the model, while runtime debugging is directed towards checking for defects during the simulation of the model and interaction of components.

The advantage of model checking is that there is little user involvement required to perform the diagnostic. A modeler can begin a model checking routine and return the next day to see the results, while original forms of model diagnostics required modelers to manually simulate and analyze the results of their models. Two traditional approaches to a manual form of analysis that are still practiced and more intuitive for new modelers are (1) pausing or slowing a simulation during defective behavior to analyze the current model variables and (2) printing out model simulation information for post-simulation analysis, otherwise known as tracing.

We analyzed a cell cycle control model that was benchmarked in the Jha et al. article on Bayesian model checking [22] to understand the differences between model checking and manual approaches. The model checking approaches yield statistically high acceptance rates, but the high sampling count required several hours to complete. Manual approaches, while much less computationally intensive, became infeasible due to the growing speed and capacity of modern computers. For example, without a way to narrow the scope of data, a modeler would have to analyze the entirety of 606 MB of simulation data for our current *E. coli* example if they were performing the tracing approach.

For the pause or slow down simulation speed approach that was successful with earlier diagnostics, nowadays a user would be very unlikely to pause a simulation at an informative frame. In order to quantify the probability of pausing at an informative frame, we determined a possible range of 3,010 instructions from the moment a simulation first expressed undesirable behavior to the frame the user could pause the simulation with average reaction speed. This was calculated by taking the average number of instructions per second performed by our simulation engine, 14,000, multiplied by the perception to finger movement reaction time, approximately 0.280 seconds depending on hand position [27]. In order for this method to be effective, the defect causing mechanism would still have to be prevalent approximately 3,010 instructions after the modeler first observed undesirable results.

A hybrid, or computer-aided debugging approach, provides the modeler with the benefits of both types of approaches. Breakpoints can be set to perform computer-aided pauses at exact behavioral events and output variables for the model are always available, narrowing the context of simulation data. Although these tools offer great aid, it should be mentioned that the feasibility of discovering a defect with a standard simulation sampling is still somewhat dependent on the expertise and knowledge of the modeler. Multiple simulations may need to be debugged in order to create a hypothesis of model behavior. In our *E. coli* example, an average of 4 full debugging simulations were required to construct a thorough analysis and correction on the mechanics of the genome-scale ATP production model behavior.

### 4.3 Comparison with Model Reduction

Although there have been efforts in systems biology towards reducing overall model complexity known as model reduction, there are key differences that specialize model slicing as a unique type of model reduction. General model reduction is a reduction that focuses on the removal of model intermediates while maintaining stoichiometry [28], most commonly via mathematical evaluations of differential equation dynamics within a model [29], [30]. Model slicing, in this context, is focused on the application to stochastic, randomized simulations with a reduction of outer information rather than the reduction of intermediates and cannot be evaluated with mathematical equations.

The reduction of model slicing is relative to a user-defined species and at a user-defined simulation time point. When a species is chosen, the slicing removes information that is not relevant to that specific species. The slice can change depending on the time point that the slice occurs. At different time points, the amounts of the species will vary and influence the availability of certain reactants which will affect the slice, as seen in the pseudo-code in Algorithm 1.

**Figure 10:**
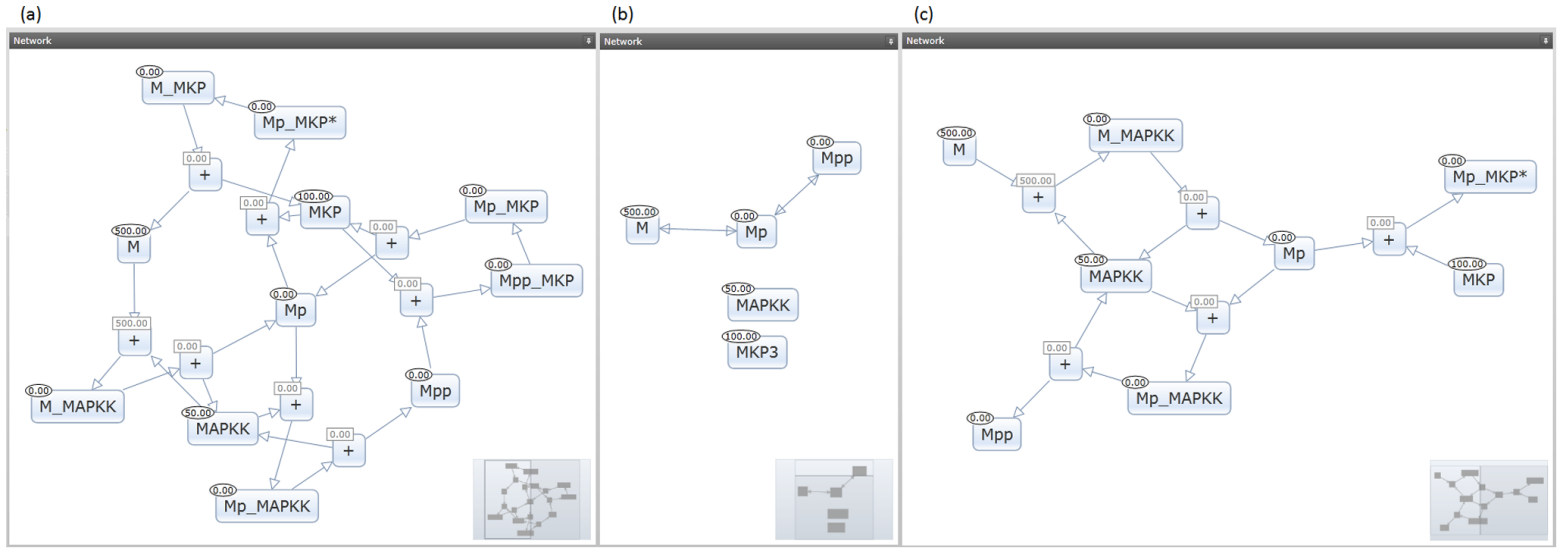
Graphical representations of the original model (a), Gay et al. model reduction (b), and MPP model slice (c). Regarding the Gay et al. model reduction, although MAPKK and MPK3 have no substrate or product role in a reaction, their concentration influences the rate of other reactions as described in the model. Furthermore, unlike model parameter constants, they have been defined as independent species and are thus represented on the figure as being part of the collection of species but without having any direct reaction connections. The reduction removes the intermediate species and directly connects M, MP and MPP while preserving the redundant reaction traffic between the species. As mentioned previously, removal of intermediates is a common approach for model reduction. The MPP model slice preserves the intermediate connections, but has a more definitive source and sink architecture resulting in less redundancy of M to MPP flow. Although fewer reactions occur and the slice is designed for a source/sink architecture, the MPP model slice is able to preserve the time unit speed of reactions to reach stabilization and demonstrates more similar behavior to the original model.

We have performed a comparison of the model reduction algorithm described in Gay et al. [29] versus our model slicing approach. The models used were MAPK cascade models, BioModels ID BIOMD0000000026 as the original and BIOMD0000000027 as the reduction, from the BioModels database. The authors of the model reduction had evaluated these models with their model comparison algorithm and had determined them to be reductions in the same family. Since the original model has a source/sink architecture with 500 units of M slowly transferring to MPP till stabilization, we used a model slice relative to the sink, MPP, and designated M as the source, the only species with a non-zero initial amount. A figure with a graphical representation of the three models is shown in Figure 10.

In Table 2 we present the quantified comparison. Data was collected from a single instance, but multiple replications showed little variation. The first three columns of our table focus on the effectiveness of the reduction, while the last three focus on the accuracy of the reduction. For the model size, a number of components was calculated using the sum of model reactions and species. The model slice was slightly larger than the reduction, but the complexity of the remaining reactions led to vast improvements in simulation speed and memory required. For the behavior of the model reductions, both reductions had slightly different end amounts for MPP, with model slicing being slightly more accurate. However, the rate of the behavior and time to behavior stabilization was much more accurate in the model slice than with the graphical model reduction method. The percentage of model behavior difference was calculated using the sum of the percentage difference in time unit count to stabilization and the percentage difference in ending MPP amount. Time of simulation stabilization was defined as the first time unit count at which the amount of MPP differed by less than 1% within the proceeding 10 time units. This definition assumes that there is no significantly different behavior in the time period between the two measurements, which is characteristic of the simulation behavior for this particular set of models. The increase in MPP amount is most drastic towards the beginning of a simulation and slowly plateaus in magnitude.

**Table 2:** Comparison of efficiency in available model reduction methodologies for systems biology models.

Model reduction methodology	Model size, number of components	Simulation runtime, seconds	Memory used, MB	Time unit count at behavior stabilization	MPP amount at stabilization	Percentage in model behavior difference
Original model	21	60.0	423	80	375	0.0%
Gay et al. model reduction	9	50.2	370	10	250	120.8%
Model slicing	13	0.8	8	80	475	26.0%

All simulations were run using an Intel Core i7 3.4 GHz, 16 GB RAM, 64-bit personal computer running Windows 7 Professional.

### 4.4 Comparison with Flux Balance Analysis

An analysis tool used to analyze the flow of metabolites through a system known as flux balance analysis (FBA) has been growing in popularity [31]. While today it is seen as a possible approach to identify bottlenecks in a system, there are still substantial qualitative differences between the flux balance analysis approach and the slice and debugging approach. The FBA approach determines the balanced state of a system as a whole, while the slice and debug approach focuses on specific areas of interest, which may be more favorable during the creation of a model where the entire model may not be finished and ready to be balanced. FBA also does not use kinetic parameter information, which the modeler may be interested in understanding how different parameter values can affect behavior. Finally, FBA uses value constraints to limit the result of the balance, whereas debugging is more interactive in that conditional breakpoints are set to prompt the modeler on next steps when a constraint is reached instead of obtaining a balanced system.

### 4.5 Reaction Graph Refinement in Future Work

#### Unidentified Co-factors

The inclusion of co-factors into the reaction graph can cause misleading information. In the 1^st^ reaction of glycolysis, an H^+^ is created during the hexokinase of glucose. H^+^ compounds are also involved in several reactions that create pyruvate as seen in the reactions below. We produced a list of common cofactors to negate this false pathway creation during upstream and downstream connections, but this technique depends on the identification of those cofactors that are not normally identified in SBML. Other compounds that are associated with reactions outside the expected pathway may cause similar effects.

- Upstream reaction:
  1. ATP +D-Glucose → ADP +D-Glucose 6-phosphate +H^+^
- Pyruvate producing downstream reaction:
  1. H^+^+ Oxaloacetate → CO2 + Pyruvate
- Alternate pyruvate producing downstream reaction:
  1. ADP +H^+^+ Phosphoenolpyruvate → ATP + Pyruvate

Co-factor list used: H^+^, AMP, ADP, ATP, CoA, NAD, NADH, NADP, NADPH, and phosphate

Ignoring these cofactors makes assumptions that they will exist in ample amount in the whole-cell. This can cause discrepancy if the assumption is not held between the slice and the whole-cell [32] as seen in the glycolysis example where ATP was quickly exhausted.

#### Compatibility of reaction weights with probabilities for the forward algorithm

As mentioned in the methods section, the predictive weights correlate with the probability a reaction will occur without having the reactants for the reaction readily available, which can make it more useful than the reaction weight. This is calculated by using the forward algorithm traditionally seen in hidden Markov models on reaction weights. However, reaction weights do not follow the same mathematical principles as probability, for example, the weights could be greater than 1. This can cause incompatibilities that may lead to misleading predictive weights. For example, in order to determine the probability that two reactions will occur consecutively, traditionally statistics would suggest multiplying both probabilities together. However, if those probabilities are instead weights, and are instead greater than 1, the multiplication will result in a product larger than either of the initial weights, which incorrectly suggests the probability of the reactions occurring consecutively is greater than the probability of either reaction occurring independently. One approach to solve this is to use another metric to convert and normalize the weights into a traditional probability, but the methodology and accuracy of such a conversion has not yet been determined.

## Conclusion

Model slicing and debugging offers a different approach that may be more intuitive for the creation or reconstruction of models of a studied system and that combines manual interactivity with computer-aided computation. During an early phase, rates and parameters that best mimic a studied system will need to be specified in order to have a model that can be simulated. The glycolysis example shows how a model that needs to be adjusted to look like glycolysis can be created by (1) identifying the desired behavior, (2) supplying possible value parameters (calculated from observed data or arbitrary in our case), (3) running the simulation and identifying that there is a defect, (4) slicing for the goal species and debugging the defect, (5) iteratively repeating until the desired behavior is met.

## Supporting Information

File S1 Diagnostic platform features, API documentation, and reaction listing of *E. coli* metabolic network.
(DOC)

## References

[1] Hunt C, Ropella G, Park S, Engelberg J (2008) Dichotomies between computational and mathematical models. Nature Biotechnology 26: 737–738. 10.1038/nbt0708-737 18612289

[2] Hucka M, Finney A, Sauro HM, Bolouri H, Doyle JC, et al (2002) The systems biology markup language (SBML): a medium for representation and exchange of biochemical network models. Bioinformatics 19: 524–531. 10.1093/bioinformatics/btg015 12611808

[3] Lopez C, Muhlich J, Bachman J, Sorger P (2013) Programming biological models in Python using PySB. Molecular Systems Biology 9: 646. 23423320 10.1038/msb.2013.1 PMC3588907

[4] Miller J, Nair R, Zhang Z, Zhao H (1997) JSIM: A Java-based simulation and animation environment. Simulation Symposium 31–42.

[5] Peters SA (2008) Evaluation of a generic physiologically based pharmacokinetic model for lineshape analysis. Clin Pharmacokinet 47(4): 261–75. 18336055 10.2165/00003088-200847040-00004

[6] Mendes P, Hoops S, Sahle S, Gauges R, Dada J, et al (2009) Computational modeling of biochemical networks using COPASI. Methods Mol Biol 500: 17–59. 19399433 10.1007/978-1-59745-525-1_2

[7] Lu TK (2010) Engineering scalable biological systems. Bioeng Bugs 1: 3780384. 10.4161/bbug.1.6.13086 PMC3056087 21468204

[8] Purnick PE, Weiss R (2009) The second wave of synthetic biology: from modules to systems. Nat Rev Mol Cell Biol 10: 410–22. 19461664 10.1038/nrm2698

[9] Roberts E, Magis A, Ortiz J, Baumeister W, Luthey-Schulten Z (2011) Noise contributions in an inducible genetic switch: a whole-cell simulation study. PLoS Comput Biol 7: e1002010. 21423716 10.1371/journal.pcbi.1002010 PMC3053318

[10] Keating SM, Bornstein B, Finney A, Hucka M (2006) SBMLToolbox: an SBML toolbox for MATLAB users. Bioinformatics 10: 1275–1277. 10.1093/bioinformatics/btl111 16574696

[11] Thiele I, Palsson B (2010) A protocol for generating a high-quality genome-scale metabolic reconstruction. Nat Protoc 5(1): 93–121. 20057383 10.1038/nprot.2009.203 PMC3125167

[12] Schellenberger J, Que R, Fleming RM, Thiele I, Orth JD, et al (2011) Quantitative prediction of cellular metabolism with constraint-based models; the COBRA Toolbox v2.0. Nat Protoc 6(9): 1290–307. 21886097 10.1038/nprot.2011.308 PMC3319681

[13] Weiser M (1984) Program slicing. IEEE Transactions on Software Engineering 10(4): 352–357.

[14] Gill S (1951) The Diagnosis of Mistakes in Programmes on the EDSAC. Proceedings of the Royal Society of London 206(1087): 538–554.

[15] Gillespie DT (1977) Exact stochastic simulation of coupled chemical reactions. J. Phys. Chem 81: 2340–2361.

[16] Gillespie DT (2007) Stochastic simulation of chemical kinetics. Annu. Rev. Phys. Chem 58: 35–55. 10.1146/annurev.physchem.58.032806.104637 17037977

[17] Jong HD (2002) Modeling and simulation of genetic regulatory systems: a literature review. J. Comp. Biol 1: 67–103. 10.1089/10665270252833208 11911796

[18] Wrzodek C, Buchel F, Ruff M, Drager A, Zell A (2013) Precise generation of systems biology models from KEGG pathways. BMC Systems Biology 7: 15. 23433509 10.1186/1752-0509-7-15 PMC3623889

[19] Orth JD, Conrad TM, Na J, Lerman JA, Nam H, et al (2011) A comprehensive genome-scale reconstruction of Escherichia coli metabolism. Molecular Systems Biology 7: 535. 21988831 10.1038/msb.2011.65 PMC3261703

[20] McCloskey D, Palsson B, Feist AM (2013) Basic and applied uses of genome-scale metabolic network reconstructions of Escherichia coli. Molecular Systems Biology 9: 661. 23632383 10.1038/msb.2013.18 PMC3658273

[21] Li C, Nagasaki M, Ueno K, Miyano S (2009) Simulation-based model checking approach to cell fate specification during Caenorhabditis elegans vulval development by hybrid functional Petri net with extension. BMC Systems Biology 3: 42. 19393101 10.1186/1752-0509-3-42 PMC2691733

[22] Jha SK, Clarke E, Langmead C, Legay A, Platzer A, et al.. (2009) A Bayesian approach to model checking biological systems. CMSB 09 Proceedings of the 7th Interational Conference on Computational Methods in Systems Biology 218–234.

[23] Kwiatkowska M, Norman G, Parker D (2008) Using probabilistic model checking in systems biology. ACM SIGMETRICS Performance Evaluation Review 35(4): 14–21.

[24] Karr JR, Sanghvi J, Macklin D, Gutschow M, Jacobs J, et al (2012) A whole-cell computational model predicts phenotype from genotype. Cell 150: 389–401. 22817898 10.1016/j.cell.2012.05.044 PMC3413483

[25] Dalchau N, Smith MJ, Martin S, Brown JR, Emmott S, et al (2012) Towards the rational design of synthetic cells with prescribed population dynamics. J. R. Soc. Interface. 9: 2883–2898. 10.1098/rsif.2012.0280 PMC3479904 22683525

[26] Pedersen M, Phillips A (2009) Towards programming languages for genetic engineering of living cells. J. R. Soc. Interface. 6: 437–450. 10.1098/rsif.2008.0516.focus PMC2843955 19369220

[27] Brass M, Bekkering H, Wohlschlager A, Prinz W (2000) Compatibility between observed and executed finger movements: comparing symbolic, spatial, and imitative cues. Brain and Cognition 44(2): 124–143. 11041986 10.1006/brcg.2000.1225

[28] Clarke BL (1992) General method for simplifying chemical networks while preserving overall stoichiometry in reduced mechanisms. J. Chem. Phys 97(6): 4066–4071.

[29] Gay S, Soliman S, Fages F (2010) A graphical method for reducing and relating models in systems biology. Bioinformatics 26(18): 575–581. 10.1093/bioinformatics/btq388 PMC2935413 20823324

[30] Petzold L, Zhu WJ (1999) Model reduction for chemical kinetics: An optimization approach. AIChE Journal 45(4): 869–886.

[31] Orth JD, Thiele I, Palsson B (2010) What is flux balance analysis? Nature Biotechnology 28: 245–248. 10.1038/nbt.1614 PMC3108565 20212490

[32] Iafolloa MA, Dong GQ, McMillen DR (2008) Increasing the efficiency of bacterial transcription simulations: When to exclude the genome without loss of accuracy. BMC Bioinformatics 9: 373. 18789148 10.1186/1471-2105-9-373 PMC2543029

