# Supplementary material for: Diagnostics for Stochastic Genome-Scale Modeling via Model Slicing and Debugging
Source: PLoS One. 2014 Nov 4;9(11):e110380. doi: 10.1371/journal.pone.0110380 (PMC4219680; doi:10.1371/journal.pone.0110380)
Supplement: File S1 — Diagnostic platform features, API documentation, and reaction listing of E. coli metabolic network. (DOC) [file pone.0110380.s001.doc]

# Supplementary Information

# Diagnostic Platform Features

| **Feature** | **Category** | **Description** | **Usage Scenario** |
| --- | --- | --- | --- |
| Model slice | Scalability, portability | A computationally calculated subset of the model using user-specified input | Can be used to investigate the production of a specific species or influencing reactions that have an effect on that species |
| Predictive weights | Behavior mechanics | Reaction kinetic law rates, weights for the probability of next occurring reaction, and upcoming probability calculated by a forward algorithm | Used to identify the likeliness of a model behavior and the impacting parameters that cause such behavior to occur |
| Multiple Simulation | Behavior mechanics | A simulation mode that runs several stochastic simulations in order to measure the stochastic fluctuation of the model | Stochastic fluctuation can be used to understand and prevent biological noise due to loose genetic design |
| Breakpoint / Conditional Breakpoint | Core model debugging | A user-specified criteria in which the simulation of a model will stop when met | Used to make detailed observations on the model state when a certain criteria is met or used to determine if the criteria is ever met at all |
| Deadlock Messaging | Core model debugging | A message notifying the user of a probable cause for a model deadlock | Information provided by deadlock messaging can be used to adjust the model to prevent future deadlocks |
| Instruction Skipping | Core model debugging | The ability to skip an instruction that would otherwise cause the model to be in an un-operable, deadlocked state | Rare occurrences of reactions or model changes that leave the simulation in a deadlocked situation can be skipped in order to view more common downstream behavior |
| Modification On-The-Fly | Core model debugging | Modifying a parameter, species, or reaction rate during the execution of a simulation | An observed species that is scarcely produced due to rare availability by default model parameters can have their downstream behavior analyzed with a modification to force production during availability |
| Network Graph | Visualization/ complementary | A network graph with animated representation of a simulation | Provides an alternate representation of the overall network and the areas of cell activity and traffic within the network |
| Concentration Plot | Visualization/ complementary | A concentration plot of population amounts from species selected in the network visualization | Measures population proportion between species and can identify the production of specific components |
| Reaction Call Stack | Visualization/ complementary | An ordered history of reactions called during the course of a simulation | Allows the reproduction of a specific model state for localized analysis of simulation behavior |

## Stochastic Fluctuation

For stochastic simulations, users can compare the population plots of multiple simulations for the same species to measure the scale of random fluctuation in a population plot overlay. This allows the user to get a better expectation of the range of fluctuation that can occur during implementation.

If implementation has already been performed and normalized expression data is available, the data can also be used to determine instances when the behavior of the organism falls outside the standard deviation of stochastic fluctuation. In such outlier cases, breakpoints will be set automatically and the user can review the closest simulation per time point of each breakpoint to evaluate possible differences between the simulation and experimentation. The algorithm for breakpoint assignment is described in Box 3.

- Simulate model *n* number of times to create simulation candidates and stochastic fluctuation
- Compare fluctuation with normalized gene expression data by the following:
  - For each species *s* in user defined expression data
    - For each time point *t* with data value *d* of species *s* in gene expression data
      - Compare value *d* with population average in simulation candidates at time *t* for species *s*
      - If *d* is out of the range of population standard deviation for all simulation candidates
        - Set a breakpoint at time *t*
        - Find simulation candidate *c* with closest population at *t* to value *d*
        - Evaluate *c* at time *t* to evaluate possible deviations in mechanics with expression data
      - Else
        - Continue to next time point

# Gene Circuit Studio Simulation Engine and Debugging Platform Documentation

## Software Implementation and Availability

The debugging platform, called Gene Circuit Studio, is primarily used as a model analysis tool and is open to any SBML model, but the library of SBML entities used to perform the model instantiation is an open framework that can be used to create new models. Software programmers can create models from scratch without prior SBML knowledge via the development support offered by the IDE and framework documentation similar to libSBML. The exception is that the package also offers implementation on stochastic SBML simulation, alleviating the developer of defining how the interactions in the model will execute. This is similar to libSBMLsim which offers built in deterministic simulation, except we offer both deterministic and stochastic simulation, the latter acknowledged as being much more realistic to actual cell behavior.

The platform was implemented in C# and the interface is implemented with an additional framework for rich internet applications called Microsoft Silverlight and a user interface framework called Infragistics NetAdvantage. Gene Circuit Studio has been tested with the simulation tests in the test suite for SBML available at SBML.org. Technical detail and documentation regarding the implementation and access specifics of the framework is available in the supplementary material. The platform is also compatible with several models from the BioModels database at the California Institute of Technology. Gene Circuit Studio is freely available for academic use and accessible at <http://gel.ym.edu.tw/gcs/>.

## Model Instantiation

In order to create a debugging-compatible mapping we implemented a software platform in C# that instantiates SBML into standardized classes of instruction objects. The object-oriented architecture allows the debugging methodology to be applied on all elements of SBML. For example, the assignment rules, events and reactions are all instantiated as mutable objects with their own properties and functions. During the simulation of the model, each object, or instruction, performs its behavior when called by the simulation engine, which uses a Gillespie Stochastic Simulation Algorithm (SSA) to determine the next instruction. The collection of rules, events and reactions are executed individually as opposed to reaction equations that can represent multiple reactions. The parameters, species and other components of the model can be observable and modifiable during the course of the simulation.

## Model Translation for Debugging Purposes

The core debugging features offered in the platform are similar to traditional debugging features found in software development environments such as Matlab and Visual Studio. Several translation efforts have been made to translate a standardized model representation format such as SBML into a script language for debugging in a more developed tool but there are often losses in data and incompatibilities that occur during such a translation that give an inaccurate representation of the model. The debugging platform is the first tool that allows direct debugging on a model representation format by mapping debugging features to an instantiation of a model. The platform also introduces several debugging and analysis features that are specific to a biological context and not commonly available in debugging platforms for engineering.

## Installation and Programmable Model Execution

1. To create a new model, create a new Silverlight project in Visual Studio
2. Add reference to “CrystalOmics Engine Models.dll” downloadable from the simulation section of the Gene Circuit Studio page at <http://gel.ym.edu.tw/gcs/>
3. Add reference to “Cell Machine Studio.dll” downloadable from the simulation section of the Gene Circuit Studio page at <http://gel.ym.edu.tw/gcs/>
4. Add reference to “System.ComponentModel.DataAnnotations.dll” which is already offered as part of the .NET framework
5. Dynamically create an instance of the Cell_Machine_Studio MainPage object and add it to the Silverlight layout.
6. Create a model object and add it to the MainPage with MainPage.CreateAndAddModel(string title, Model startModel)
7. Compile and run the application, the application will start with the programmed model already loaded
8. When the application is running, it can always load other models by File -> Load

## Public Members of the Model Class

- Rules
  - List of assignment and rate rules in the model
- Constraints
  - List of constraints in the model
- Events
  - List of events in the model
- InitialAssignments
  - List of initial assignments in the model
- Parameters
  - List of parameters in the model
- Compartments
  - List of compartments in the model
- Species
  - List of species in the model
- Reactions
  - List of reactions in the model
- CallHistory
  - List of executed instructions
- PassedTime
  - Current time
- ModelErrors
  - A list of errors from SBML model parsing
- ExecutedInstructionsAtCurrentTime
  - The assignments already assigned at the current time point
- DelayedEvents
  - A list of events that have been triggered by not yet executed due to delays in the events
- ExecutionStatus
  - An integer to determine the status of the most recent executed instruction
    - 0 = error free, 1 = negative species, 2 = missing variable

## Public Functions of the Model Class

- XMLToObjects(Stream sbmlXml)
  - Takes a file stream and returns a parsed model object with instuctions and data members
- InstructionIteration()
  - Executes an iteration of an instruction cycle, which includes executed the selected instruction if one exists and returning the next determined instruction
- DetermineNextInstruction()
  - Usually only called from within InstructionIteration(), this determines what the next instruction should be
- Execute()
  - Usually only called from within InstructionIteration(), this executes the current instruction if one exists
- FindAndAssignVariable(string variableName, double value, Instruction nextInstruction)
  - Assigns a generic variable’s value to a specific model object

## Other

- Documentation regarding SBML and properties implemented in the C# programmable model are available at sbml.org

# Reaction Listing in E.coli Metabolic Network

| R_DM_4CRSOL | Sink needed to allow p-Cresol to leave system |
| --- | --- |
| R_DM_5DRIB | Sink needed to allow 5'-deoxyribose to leave system |
| R_DM_AACALD | Sink needed to allow aminoacetaldehyde to leave system |
| R_DM_AMOB | Sink needed to allow S-Adenosyl-4-methylthio-2-oxobutanoate to leave system |
| R_DM_MTHTHF | Sink needed to allow (2R,4S)-2-methyl-2,3,3,4-tetrahydroxytetrahydrofuran to leave system |
| R_DM_OXAM | Sink needed to allow oxamate to leave system |
| R_Ec_biomass_iJO1366_WT_53p95M | E. coli biomass objective function (iJO1366) - WT - with 53.95 GAM estimate |
| R_Ec_biomass_iJO1366_core_53p95M | E. coli biomass objective function (iJO1366) - core - with 53.95 GAM estimate |
| R_EX_12ppd_DASH_R_LPAREN_e_RPAREN_ | (R)-Propane-1,2-diol exchange |
| R_EX_12ppd_DASH_S_LPAREN_e_RPAREN_ | (S)-Propane-1,2-diol exchange |
| R_EX_14glucan_LPAREN_e_RPAREN_ | 1,4-alpha-D-glucan exchange |
| R_EX_15dap_LPAREN_e_RPAREN_ | 1,5-Diaminopentane exchange |
| R_EX_23camp_LPAREN_e_RPAREN_ | 2',3'-Cyclic AMP exchange |
| R_EX_23ccmp_LPAREN_e_RPAREN_ | 2',3'-Cyclic CMP exchange |
| R_EX_23cgmp_LPAREN_e_RPAREN_ | 2',3'-Cyclic GMP exchange |
| R_EX_23cump_LPAREN_e_RPAREN_ | 2',3'-Cyclic UMP exchange |
| R_EX_23dappa_LPAREN_e_RPAREN_ | 2,3-diaminopropionate exchange |
| R_EX_26dap_DASH_M_LPAREN_e_RPAREN_ | meso-2,6-Diaminoheptanedioate exchange |
| R_EX_2ddglcn_LPAREN_e_RPAREN_ | 2-Dehydro-3-deoxy-D-gluconate exchange |
| R_EX_34dhpac_LPAREN_e_RPAREN_ | 3,4-Dihydroxyphenylacetaldehyde exchange |
| R_EX_3amp_LPAREN_e_RPAREN_ | 3'-AMP exchange |
| R_EX_3cmp_LPAREN_e_RPAREN_ | 3'-cmp exchange |
| R_EX_3gmp_LPAREN_e_RPAREN_ | 3'-GMP exchange |
| R_EX_3hcinnm_LPAREN_e_RPAREN_ | 3-hydroxycinnamic acid exchange |
| R_EX_3hpp_LPAREN_e_RPAREN_ | 3-Hydroxypropanoate exchange |
| R_EX_3hpppn_LPAREN_e_RPAREN_ | 3-(3-hydroxy-phenyl)propionate exchange |
| R_EX_3ump_LPAREN_e_RPAREN_ | 3'-UMP exchange |
| R_EX_4abut_LPAREN_e_RPAREN_ | 4-Aminobutanoate exchange |
| R_EX_4hoxpacd_LPAREN_e_RPAREN_ | 4-Hydroxyphenylacetaldehyde exchange |
| R_EX_5dglcn_LPAREN_e_RPAREN_ | 5-Dehydro-D-gluconate exchange |
| R_EX_5mtr_LPAREN_e_RPAREN_ | 5-Methylthio-D-ribose exchange |
| R_EX_LalaDglu_LPAREN_e_RPAREN_ | L-alanine-D-glutamate exchange |
| R_EX_LalaDgluMdap_LPAREN_e_RPAREN_ | L-alanine-D-glutamate-meso-2,6-diaminoheptanedioate exchange |
| R_EX_LalaDgluMdapDala_LPAREN_e_RPAREN_ | L-alanine-D-glutamate-meso-2,6-diaminoheptanedioate-D-alanine exchange |
| R_EX_LalaLglu_LPAREN_e_RPAREN_ | L-alanine-L-glutamate exchange |
| R_EX_ac_LPAREN_e_RPAREN_ | Acetate exchange |
| R_EX_acac_LPAREN_e_RPAREN_ | Acetoacetate exchange |
| R_EX_acald_LPAREN_e_RPAREN_ | Acetaldehyde exchange |
| R_EX_acgal_LPAREN_e_RPAREN_ | N-Acetyl-D-galactosamine exchange |
| R_EX_acgal1p_LPAREN_e_RPAREN_ | N-Acetyl-D-galactosamine 1-phosphate exchange |
| R_EX_acgam_LPAREN_e_RPAREN_ | N-Acetyl-D-glucosamine exchange |
| R_EX_acgam1p_LPAREN_e_RPAREN_ | N-Acetyl-D-glucosamine 1-phosphate exchange |
| R_EX_acmana_LPAREN_e_RPAREN_ | N-Acetyl-D-mannosamine exchange |
| R_EX_acmum_LPAREN_e_RPAREN_ | N-Acetylmuramate exchange |
| R_EX_acnam_LPAREN_e_RPAREN_ | N-Acetylneuraminate exchange |
| R_EX_acolipa_LPAREN_e_RPAREN_ | 4-Amino-4-deoxy-L-arabinose modified core oligosaccharide lipid A exchange |
| R_EX_acser_LPAREN_e_RPAREN_ | O-Acetyl-L-serine exchange |
| R_EX_ade_LPAREN_e_RPAREN_ | Adenine exchange |
| R_EX_adn_LPAREN_e_RPAREN_ | Adenosine exchange |
| R_EX_adocbl_LPAREN_e_RPAREN_ | Adenosylcobalamin exchange |
| R_EX_ag_LPAREN_e_RPAREN_ | silver exchange |
| R_EX_agm_LPAREN_e_RPAREN_ | Agmatine exchange |
| R_EX_akg_LPAREN_e_RPAREN_ | 2-Oxoglutarate exchange |
| R_EX_ala_DASH_B_LPAREN_e_RPAREN_ | beta-Alanine exchange |
| R_EX_ala_DASH_D_LPAREN_e_RPAREN_ | D-Alanine exchange |
| R_EX_ala_DASH_L_LPAREN_e_RPAREN_ | L-Alanine exchange |
| R_EX_alaala_LPAREN_e_RPAREN_ | D-Alanyl-D-alanine exchange |
| R_EX_all_DASH_D_LPAREN_e_RPAREN_ | D-Allose exchange |
| R_EX_alltn_LPAREN_e_RPAREN_ | Allantoin exchange |
| R_EX_amp_LPAREN_e_RPAREN_ | AMP exchange |
| R_EX_anhgm_LPAREN_e_RPAREN_ | N-Acetyl-D-glucosamine(anhydrous)N-Acetylmuramic acid exchange |
| R_EX_arab_DASH_L_LPAREN_e_RPAREN_ | L-Arabinose exchange |
| R_EX_arbt_LPAREN_e_RPAREN_ | Arbutin exchange |
| R_EX_arbtn_LPAREN_e_RPAREN_ | aerobactin minus Fe3 exchange |
| R_EX_arbtn_DASH_fe3_LPAREN_e_RPAREN_ | Aerobactin exchange |
| R_EX_arg_DASH_L_LPAREN_e_RPAREN_ | L-Arginine exchange |
| R_EX_ascb_DASH_L_LPAREN_e_RPAREN_ | L-Ascorbate exchange |
| R_EX_asn_DASH_L_LPAREN_e_RPAREN_ | L-Asparagine exchange |
| R_EX_aso3_LPAREN_e_RPAREN_ | arsenite exchange |
| R_EX_asp_DASH_L_LPAREN_e_RPAREN_ | L-Aspartate exchange |
| R_EX_btn_LPAREN_e_RPAREN_ | Biotin exchange |
| R_EX_but_LPAREN_e_RPAREN_ | Butyrate (n-C4:0) exchange |
| R_EX_butso3_LPAREN_e_RPAREN_ | butanesulfonate exchange |
| R_EX_ca2_LPAREN_e_RPAREN_ | Calcium exchange |
| R_EX_cbi_LPAREN_e_RPAREN_ | Cobinamide exchange |
| R_EX_cbl1_LPAREN_e_RPAREN_ | Cob(I)alamin exchange |
| R_EX_cd2_LPAREN_e_RPAREN_ | Cadmium exchange |
| R_EX_cgly_LPAREN_e_RPAREN_ | Cys-Gly exchange |
| R_EX_chol_LPAREN_e_RPAREN_ | Choline exchange |
| R_EX_chtbs_LPAREN_e_RPAREN_ | chitobiose exchange |
| R_EX_cit_LPAREN_e_RPAREN_ | Citrate exchange |
| R_EX_cl_LPAREN_e_RPAREN_ | Chloride exchange |
| R_EX_cm_LPAREN_e_RPAREN_ | Chloramphenicol exchange |
| R_EX_cmp_LPAREN_e_RPAREN_ | CMP exchange |
| R_EX_co2_LPAREN_e_RPAREN_ | CO2 exchange |
| R_EX_cobalt2_LPAREN_e_RPAREN_ | Co2+ exchange |
| R_EX_colipa_LPAREN_e_RPAREN_ | core oligosaccharide lipid A exchange |
| R_EX_colipap_LPAREN_e_RPAREN_ | core oligosaccharide lipid A diphosphate exchange |
| R_EX_cpgn_LPAREN_e_RPAREN_ | coprogen exchange |
| R_EX_cpgn_DASH_un_LPAREN_e_RPAREN_ | coprogen unloaded (no Fe(III)) exchange |
| R_EX_crn_LPAREN_e_RPAREN_ | L-Carnitine exchange |
| R_EX_crn_DASH_D_LPAREN_e_RPAREN_ | D-carnitine exchange |
| R_EX_csn_LPAREN_e_RPAREN_ | Cytosine exchange |
| R_EX_cu_LPAREN_e_RPAREN_ | Cu+ exchange |
| R_EX_cu2_LPAREN_e_RPAREN_ | Cu2+ exchange |
| R_EX_cyan_LPAREN_e_RPAREN_ | Hydrogen cyanide exchange |
| R_EX_cynt_LPAREN_e_RPAREN_ | Cyanate exchange |
| R_EX_cys_DASH_D_LPAREN_e_RPAREN_ | D-Cysteine exchange |
| R_EX_cys_DASH_L_LPAREN_e_RPAREN_ | L-Cysteine exchange |
| R_EX_cytd_LPAREN_e_RPAREN_ | Cytidine exchange |
| R_EX_dad_DASH_2_LPAREN_e_RPAREN_ | Deoxyadenosine exchange |
| R_EX_damp_LPAREN_e_RPAREN_ | dAMP exchange |
| R_EX_dca_LPAREN_e_RPAREN_ | Decanoate (n-C10:0) exchange |
| R_EX_dcmp_LPAREN_e_RPAREN_ | dCMP exchange |
| R_EX_dcyt_LPAREN_e_RPAREN_ | Deoxycytidine exchange |
| R_EX_ddca_LPAREN_e_RPAREN_ | Dodecanoate (n-C12:0) exchange |
| R_EX_dgmp_LPAREN_e_RPAREN_ | dGMP exchange |
| R_EX_dgsn_LPAREN_e_RPAREN_ | Deoxyguanosine exchange |
| R_EX_dha_LPAREN_e_RPAREN_ | Dihydroxyacetone exchange |
| R_EX_dimp_LPAREN_e_RPAREN_ | dIMP exchange |
| R_EX_din_LPAREN_e_RPAREN_ | Deoxyinosine exchange |
| R_EX_dms_LPAREN_e_RPAREN_ | Dimethyl sulfide exchange |
| R_EX_dmso_LPAREN_e_RPAREN_ | Dimethyl sulfoxide exchange |
| R_EX_dopa_LPAREN_e_RPAREN_ | Dopamine exchange |
| R_EX_doxrbcn_LPAREN_e_RPAREN_ | doxorubicin exchange |
| R_EX_dtmp_LPAREN_e_RPAREN_ | dTMP exchange |
| R_EX_dump_LPAREN_e_RPAREN_ | dUMP exchange |
| R_EX_duri_LPAREN_e_RPAREN_ | Deoxyuridine exchange |
| R_EX_eca4colipa_LPAREN_e_RPAREN_ | (enterobacterial common antigen)x4 core oligosaccharide lipid A exchange |
| R_EX_enlipa_LPAREN_e_RPAREN_ | phosphoethanolamine KDO(2)-lipid (A) exchange |
| R_EX_enter_LPAREN_e_RPAREN_ | Enterochelin exchange |
| R_EX_etha_LPAREN_e_RPAREN_ | Ethanolamine exchange |
| R_EX_ethso3_LPAREN_e_RPAREN_ | ethanesulfonate exchange |
| R_EX_etoh_LPAREN_e_RPAREN_ | Ethanol exchange |
| R_EX_f6p_LPAREN_e_RPAREN_ | D-fructose 6-phosphate exchange |
| R_EX_fald_LPAREN_e_RPAREN_ | Formaldehyde exchange |
| R_EX_fe2_LPAREN_e_RPAREN_ | Fe2+ exchange |
| R_EX_fe3_LPAREN_e_RPAREN_ | Fe3+ exchange |
| R_EX_fe3dcit_LPAREN_e_RPAREN_ | Fe(III)dicitrate exchange |
| R_EX_fe3dhbzs_LPAREN_e_RPAREN_ | ferric 2,3-dihydroxybenzoylserine exchange |
| R_EX_fe3hox_LPAREN_e_RPAREN_ | Fe(III)hydroxamate exchange |
| R_EX_fe3hox_DASH_un_LPAREN_e_RPAREN_ | Fe(III)hydoxamate, unloaded exchange |
| R_EX_fecrm_LPAREN_e_RPAREN_ | Ferrichrome exchange |
| R_EX_fecrm_DASH_un_LPAREN_e_RPAREN_ | Ferrichrome minus Fe(III) exchange |
| R_EX_feenter_LPAREN_e_RPAREN_ | Fe-enterobactin exchange |
| R_EX_feoxam_LPAREN_e_RPAREN_ | ferroxamine exchange |
| R_EX_feoxam_DASH_un_LPAREN_e_RPAREN_ | ferroxamine minus Fe(3) exchange |
| R_EX_for_LPAREN_e_RPAREN_ | Formate exchange |
| R_EX_fru_LPAREN_e_RPAREN_ | D-Fructose exchange |
| R_EX_frulys_LPAREN_e_RPAREN_ | fructoselysine exchange |
| R_EX_fruur_LPAREN_e_RPAREN_ | D-Fructuronate exchange |
| R_EX_fuc_DASH_L_LPAREN_e_RPAREN_ | L-Fucose exchange |
| R_EX_fum_LPAREN_e_RPAREN_ | Fumarate exchange |
| R_EX_fusa_LPAREN_e_RPAREN_ | fusidic acid exchange |
| R_EX_g1p_LPAREN_e_RPAREN_ | D-Glucose 1-phosphate exchange |
| R_EX_g3pc_LPAREN_e_RPAREN_ | sn-Glycero-3-phosphocholine exchange |
| R_EX_g3pe_LPAREN_e_RPAREN_ | sn-Glycero-3-phosphoethanolamine exchange |
| R_EX_g3pg_LPAREN_e_RPAREN_ | Glycerophosphoglycerol exchange |
| R_EX_g3pi_LPAREN_e_RPAREN_ | sn-Glycero-3-phospho-1-inositol exchange |
| R_EX_g3ps_LPAREN_e_RPAREN_ | Glycerophosphoserine exchange |
| R_EX_g6p_LPAREN_e_RPAREN_ | D-Glucose 6-phosphate exchange |
| R_EX_gal_LPAREN_e_RPAREN_ | D-Galactose exchange |
| R_EX_gal_DASH_bD_LPAREN_e_RPAREN_ | beta D-Galactose exchange |
| R_EX_gal1p_LPAREN_e_RPAREN_ | alpha-D-Galactose 1-phosphate exchange |
| R_EX_galct_DASH_D_LPAREN_e_RPAREN_ | D-Galactarate exchange |
| R_EX_galctn_DASH_D_LPAREN_e_RPAREN_ | D-Galactonate exchange |
| R_EX_galctn_DASH_L_LPAREN_e_RPAREN_ | L-Galactonate exchange |
| R_EX_galt_LPAREN_e_RPAREN_ | Galactitol exchange |
| R_EX_galur_LPAREN_e_RPAREN_ | D-Galacturonate exchange |
| R_EX_gam_LPAREN_e_RPAREN_ | D-Glucosamine exchange |
| R_EX_gam6p_LPAREN_e_RPAREN_ | D-Glucosamine 6-phosphate exchange |
| R_EX_gbbtn_LPAREN_e_RPAREN_ | gamma-butyrobetaine exchange |
| R_EX_gdp_LPAREN_e_RPAREN_ | GDP exchange |
| R_EX_glc_LPAREN_e_RPAREN_ | D-Glucose exchange |
| R_EX_glcn_LPAREN_e_RPAREN_ | D-Gluconate exchange |
| R_EX_glcr_LPAREN_e_RPAREN_ | D-Glucarate exchange |
| R_EX_glcur_LPAREN_e_RPAREN_ | D-Glucuronate exchange |
| R_EX_glcur1p_LPAREN_e_RPAREN_ | D-Glucuronate 1-phosphate exchange |
| R_EX_gln_DASH_L_LPAREN_e_RPAREN_ | L-Glutamine exchange |
| R_EX_glu_DASH_L_LPAREN_e_RPAREN_ | L-Glutamate exchange |
| R_EX_gly_LPAREN_e_RPAREN_ | Glycine exchange |
| R_EX_glyald_LPAREN_e_RPAREN_ | D-Glyceraldehyde exchange |
| R_EX_glyb_LPAREN_e_RPAREN_ | Glycine betaine exchange |
| R_EX_glyc_LPAREN_e_RPAREN_ | Glycerol exchange |
| R_EX_glyc_DASH_R_LPAREN_e_RPAREN_ | (R)-Glycerate exchange |
| R_EX_glyc2p_LPAREN_e_RPAREN_ | Glycerol 2-phosphate exchange |
| R_EX_glyc3p_LPAREN_e_RPAREN_ | Glycerol 3-phosphate exchange |
| R_EX_glyclt_LPAREN_e_RPAREN_ | Glycolate exchange |
| R_EX_gmp_LPAREN_e_RPAREN_ | GMP exchange |
| R_EX_gsn_LPAREN_e_RPAREN_ | Guanosine exchange |
| R_EX_gthox_LPAREN_e_RPAREN_ | Oxidized glutathione exchange |
| R_EX_gthrd_LPAREN_e_RPAREN_ | Reduced glutathione exchange |
| R_EX_gtp_LPAREN_e_RPAREN_ | GTP exchange |
| R_EX_gua_LPAREN_e_RPAREN_ | Guanine exchange |
| R_EX_h_LPAREN_e_RPAREN_ | H+ exchange |
| R_EX_h2_LPAREN_e_RPAREN_ | H2 exchange |
| R_EX_h2o_LPAREN_e_RPAREN_ | H2O exchange |
| R_EX_h2o2_LPAREN_e_RPAREN_ | Hydrogen peroxide exchange |
| R_EX_h2s_LPAREN_e_RPAREN_ | Hydrogen sulfide exchange |
| R_EX_hacolipa_LPAREN_e_RPAREN_ | hepta-acylated core oligosaccharide lipid A (E. coli) exchange |
| R_EX_halipa_LPAREN_e_RPAREN_ | hepta-acylated KDO(2)-lipid (A) exchange |
| R_EX_hdca_LPAREN_e_RPAREN_ | Hexadecanoate (n-C16:0) exchange |
| R_EX_hdcea_LPAREN_e_RPAREN_ | hexadecenoate (n-C16:1) exchange |
| R_EX_hg2_LPAREN_e_RPAREN_ | Hg2+ exchange |
| R_EX_his_DASH_L_LPAREN_e_RPAREN_ | L-Histidine exchange |
| R_EX_hom_DASH_L_LPAREN_e_RPAREN_ | L-Homoserine exchange |
| R_EX_hxa_LPAREN_e_RPAREN_ | Hexanoate (n-C6:0) exchange |
| R_EX_hxan_LPAREN_e_RPAREN_ | Hypoxanthine exchange |
| R_EX_idon_DASH_L_LPAREN_e_RPAREN_ | L-Idonate exchange |
| R_EX_ile_DASH_L_LPAREN_e_RPAREN_ | L-Isoleucine exchange |
| R_EX_imp_LPAREN_e_RPAREN_ | IMP exchange |
| R_EX_indole_LPAREN_e_RPAREN_ | Indole exchange |
| R_EX_inost_LPAREN_e_RPAREN_ | myo-Inositol exchange |
| R_EX_ins_LPAREN_e_RPAREN_ | Inosine exchange |
| R_EX_isetac_LPAREN_e_RPAREN_ | Isethionic acid exchange |
| R_EX_k_LPAREN_e_RPAREN_ | K+ exchange |
| R_EX_kdo2lipid4_LPAREN_e_RPAREN_ | KDO(2)-lipid IV(A) exchange |
| R_EX_lac_DASH_D_LPAREN_e_RPAREN_ | D-lactate exchange |
| R_EX_lac_DASH_L_LPAREN_e_RPAREN_ | L-Lactate exchange |
| R_EX_lcts_LPAREN_e_RPAREN_ | Lactose exchange |
| R_EX_leu_DASH_L_LPAREN_e_RPAREN_ | L-Leucine exchange |
| R_EX_lipa_LPAREN_e_RPAREN_ | KDO(2)-lipid (A) exchange |
| R_EX_lipa_cold_LPAREN_e_RPAREN_ | cold adapted KDO(2)-lipid (A) exchange |
| R_EX_lipoate_LPAREN_e_RPAREN_ | Lipoate exchange |
| R_EX_lys_DASH_L_LPAREN_e_RPAREN_ | L-Lysine exchange |
| R_EX_lyx_DASH_L_LPAREN_e_RPAREN_ | L-Lyxose exchange |
| R_EX_mal_DASH_D_LPAREN_e_RPAREN_ | D-Malate exchange |
| R_EX_mal_DASH_L_LPAREN_e_RPAREN_ | L-Malate exchange |
| R_EX_malt_LPAREN_e_RPAREN_ | Maltose exchange |
| R_EX_malthx_LPAREN_e_RPAREN_ | Maltohexaose exchange |
| R_EX_maltpt_LPAREN_e_RPAREN_ | Maltopentaose exchange |
| R_EX_malttr_LPAREN_e_RPAREN_ | Maltotriose exchange |
| R_EX_maltttr_LPAREN_e_RPAREN_ | Maltotetraose exchange |
| R_EX_man_LPAREN_e_RPAREN_ | D-Mannose exchange |
| R_EX_man6p_LPAREN_e_RPAREN_ | D-Mannose 6-phosphate exchange |
| R_EX_manglyc_LPAREN_e_RPAREN_ | 2(alpha-D-Mannosyl)-D-glycerate exchange |
| R_EX_melib_LPAREN_e_RPAREN_ | Melibiose exchange |
| R_EX_meoh_LPAREN_e_RPAREN_ | methanol exchange |
| R_EX_met_DASH_D_LPAREN_e_RPAREN_ | D-Methionine exchange |
| R_EX_met_DASH_L_LPAREN_e_RPAREN_ | L-Methionine exchange |
| R_EX_metsox_DASH_R_DASH_L_LPAREN_e_RPAREN_ | L-methionine-R-sulfoxide exchange |
| R_EX_metsox_DASH_S_DASH_L_LPAREN_e_RPAREN_ | L-Methionine S-oxide exchange |
| R_EX_mg2_LPAREN_e_RPAREN_ | Mg exchange |
| R_EX_mincyc_LPAREN_e_RPAREN_ | minocycline exchange |
| R_EX_minohp_LPAREN_e_RPAREN_ | myo-Inositol hexakisphosphate exchange |
| R_EX_mmet_LPAREN_e_RPAREN_ | S-Methyl-L-methionine exchange |
| R_EX_mn2_LPAREN_e_RPAREN_ | Mn2+ exchange |
| R_EX_mnl_LPAREN_e_RPAREN_ | D-Mannitol exchange |
| R_EX_mobd_LPAREN_e_RPAREN_ | Molybdate exchange |
| R_EX_mso3_LPAREN_e_RPAREN_ | methanesulfonate exchange |
| R_EX_n2o_LPAREN_e_RPAREN_ | Nitrous oxide exchange |
| R_EX_na1_LPAREN_e_RPAREN_ | Sodium exchange |
| R_EX_nac_LPAREN_e_RPAREN_ | Nicotinate exchange |
| R_EX_nh4_LPAREN_e_RPAREN_ | Ammonia exchange |
| R_EX_ni2_LPAREN_e_RPAREN_ | Ni2+ exchange |
| R_EX_nmn_LPAREN_e_RPAREN_ | NMN exchange |
| R_EX_no_LPAREN_e_RPAREN_ | Nitric oxide exchange |
| R_EX_no2_LPAREN_e_RPAREN_ | Nitrite exchange |
| R_EX_no3_LPAREN_e_RPAREN_ | Nitrate exchange |
| R_EX_novbcn_LPAREN_e_RPAREN_ | novobiocin exchange |
| R_EX_o16a4colipa_LPAREN_e_RPAREN_ | (O16 antigen)x4 core oligosaccharide lipid A exchange |
| R_EX_o2_LPAREN_e_RPAREN_ | O2 exchange |
| R_EX_o2s_LPAREN_e_RPAREN_ | Superoxide anion exchange |
| R_EX_ocdca_LPAREN_e_RPAREN_ | octadecanoate (n-C18:0) exchange |
| R_EX_ocdcea_LPAREN_e_RPAREN_ | octadecenoate (n-C18:1) exchange |
| R_EX_octa_LPAREN_e_RPAREN_ | octanoate (n-C8:0) exchange |
| R_EX_orn_LPAREN_e_RPAREN_ | Ornithine exchange |
| R_EX_orot_LPAREN_e_RPAREN_ | Orotate exchange |
| R_EX_pacald_LPAREN_e_RPAREN_ | Phenylacetaldehyde exchange |
| R_EX_peamn_LPAREN_e_RPAREN_ | Phenethylamine exchange |
| R_EX_phe_DASH_L_LPAREN_e_RPAREN_ | L-Phenylalanine exchange |
| R_EX_pheme_LPAREN_e_RPAREN_ | Protoheme exchange |
| R_EX_pi_LPAREN_e_RPAREN_ | Phosphate exchange |
| R_EX_pnto_DASH_R_LPAREN_e_RPAREN_ | (R)-Pantothenate exchange |
| R_EX_ppa_LPAREN_e_RPAREN_ | Propionate exchange |
| R_EX_ppal_LPAREN_e_RPAREN_ | Propanal exchange |
| R_EX_pppn_LPAREN_e_RPAREN_ | Phenylpropanoate exchange |
| R_EX_ppt_LPAREN_e_RPAREN_ | Phosphonate exchange |
| R_EX_pro_DASH_L_LPAREN_e_RPAREN_ | L-Proline exchange |
| R_EX_progly_LPAREN_e_RPAREN_ | L-Prolinylglycine exchange |
| R_EX_psclys_LPAREN_e_RPAREN_ | psicoselysine exchange |
| R_EX_pser_DASH_L_LPAREN_e_RPAREN_ | O-Phospho-L-serine exchange |
| R_EX_ptrc_LPAREN_e_RPAREN_ | Putrescine exchange |
| R_EX_pydam_LPAREN_e_RPAREN_ | Pyridoxamine exchange |
| R_EX_pydx_LPAREN_e_RPAREN_ | Pyridoxal exchange |
| R_EX_pydxn_LPAREN_e_RPAREN_ | Pyridoxine exchange |
| R_EX_pyr_LPAREN_e_RPAREN_ | Pyruvate exchange |
| R_EX_quin_LPAREN_e_RPAREN_ | Quinate exchange |
| R_EX_r5p_LPAREN_e_RPAREN_ | alpha-D-Ribose 5-phosphate exchange |
| R_EX_rfamp_LPAREN_e_RPAREN_ | rifampin exchange |
| R_EX_rib_DASH_D_LPAREN_e_RPAREN_ | D-Ribose exchange |
| R_EX_rmn_LPAREN_e_RPAREN_ | L-Rhamnose exchange |
| R_EX_sbt_DASH_D_LPAREN_e_RPAREN_ | D-Sorbitol exchange |
| R_EX_sel_LPAREN_e_RPAREN_ | Selenate exchange |
| R_EX_ser_DASH_D_LPAREN_e_RPAREN_ | D-Serine exchange |
| R_EX_ser_DASH_L_LPAREN_e_RPAREN_ | L-Serine exchange |
| R_EX_skm_LPAREN_e_RPAREN_ | Shikimate exchange |
| R_EX_slnt_LPAREN_e_RPAREN_ | selenite exchange |
| R_EX_so2_LPAREN_e_RPAREN_ | sulfur dioxide exchange |
| R_EX_so3_LPAREN_e_RPAREN_ | Sulfite exchange |
| R_EX_so4_LPAREN_e_RPAREN_ | Sulfate exchange |
| R_EX_spmd_LPAREN_e_RPAREN_ | Spermidine exchange |
| R_EX_succ_LPAREN_e_RPAREN_ | Succinate exchange |
| R_EX_sucr_LPAREN_e_RPAREN_ | Sucrose exchange |
| R_EX_sulfac_LPAREN_e_RPAREN_ | sulfoacetate exchange |
| R_EX_tartr_DASH_D_LPAREN_e_RPAREN_ | D-tartrate exchange |
| R_EX_tartr_DASH_L_LPAREN_e_RPAREN_ | L-tartrate exchange |
| R_EX_taur_LPAREN_e_RPAREN_ | Taurine exchange |
| R_EX_tcynt_LPAREN_e_RPAREN_ | Thiocyanate exchange |
| R_EX_thm_LPAREN_e_RPAREN_ | Thiamin exchange |
| R_EX_thr_DASH_L_LPAREN_e_RPAREN_ | L-Threonine exchange |
| R_EX_thrp_LPAREN_e_RPAREN_ | L-Threonine O-3-phosphate exchange |
| R_EX_thym_LPAREN_e_RPAREN_ | Thymine exchange |
| R_EX_thymd_LPAREN_e_RPAREN_ | Thymidine exchange |
| R_EX_tma_LPAREN_e_RPAREN_ | Trimethylamine exchange |
| R_EX_tmao_LPAREN_e_RPAREN_ | Trimethylamine N-oxide exchange |
| R_EX_tre_LPAREN_e_RPAREN_ | Trehalose exchange |
| R_EX_trp_DASH_L_LPAREN_e_RPAREN_ | L-Tryptophan exchange |
| R_EX_tsul_LPAREN_e_RPAREN_ | Thiosulfate exchange |
| R_EX_ttdca_LPAREN_e_RPAREN_ | tetradecanoate (n-C14:0) exchange |
| R_EX_ttdcea_LPAREN_e_RPAREN_ | tetradecenoate (n-C14:1) exchange |
| R_EX_ttrcyc_LPAREN_e_RPAREN_ | tetracycline exchange |
| R_EX_tungs_LPAREN_e_RPAREN_ | tungstate exchange |
| R_EX_tym_LPAREN_e_RPAREN_ | Tyramine exchange |
| R_EX_tyr_DASH_L_LPAREN_e_RPAREN_ | L-Tyrosine exchange |
| R_EX_tyrp_LPAREN_e_RPAREN_ | Phosphotyrosine exchange |
| R_EX_uacgam_LPAREN_e_RPAREN_ | UDP-N-acetyl-D-glucosamine exchange |
| R_EX_udpacgal_LPAREN_e_RPAREN_ | UDP-N-acetyl-D-galactosamine exchange |
| R_EX_udpg_LPAREN_e_RPAREN_ | UDPglucose exchange |
| R_EX_udpgal_LPAREN_e_RPAREN_ | UDPgalactose exchange |
| R_EX_udpglcur_LPAREN_e_RPAREN_ | UDP-D-glucuronate exchange |
| R_EX_ump_LPAREN_e_RPAREN_ | UMP exchange |
| R_EX_ura_LPAREN_e_RPAREN_ | Uracil exchange |
| R_EX_urea_LPAREN_e_RPAREN_ | Urea exchange |
| R_EX_uri_LPAREN_e_RPAREN_ | Uridine exchange |
| R_EX_val_DASH_L_LPAREN_e_RPAREN_ | L-Valine exchange |
| R_EX_xan_LPAREN_e_RPAREN_ | Xanthine exchange |
| R_EX_xmp_LPAREN_e_RPAREN_ | Xanthosine 5'-phosphate exchange |
| R_EX_xtsn_LPAREN_e_RPAREN_ | Xanthosine exchange |
| R_EX_xyl_DASH_D_LPAREN_e_RPAREN_ | D-Xylose exchange |
| R_EX_xylu_DASH_L_LPAREN_e_RPAREN_ | L-Xylulose exchange |
| R_EX_zn2_LPAREN_e_RPAREN_ | Zinc exchange |
| R_12DGR120tipp | 1,2 diacylglycerol transport via flipping (periplasm to cytoplasm, n-C12:0) |
| R_12DGR140tipp | 1,2 diacylglycerol transport via flipping (periplasm to cytoplasm, n-C14:0) |
| R_12DGR141tipp | 1,2 diacylglycerol transport via flipping (periplasm to cytoplasm, n-C14:1) |
| R_12DGR160tipp | 1,2 diacylglycerol transport via flipping (periplasm to cytoplasm, n-C16:0) |
| R_12DGR161tipp | 1,2 diacylglycerol transport via flipping (periplasm to cytoplasm, n-C16:1) |
| R_12DGR180tipp | 1,2 diacylglycerol transport via flipping (periplasm to cytoplasm, n-C18:0) |
| R_12DGR181tipp | 1,2 diacylglycerol transport via flipping (periplasm to cytoplasm, n-C18:1) |
| R_12PPDRtex | (R)-Propane-1,2-diol transport via diffusion (extracellular to periplasm) |
| R_12PPDRtpp | (R)-Propane-1,2-diol facilitated transport (periplasm) |
| R_12PPDStex | (S)-Propane-1,2-diol transport via diffusion (extracellular to periplasm) |
| R_12PPDStpp | (S)-Propane-1,2-diol facilitated transport (periplasm) |
| R_14GLUCANabcpp | 1,4-alpha-D-glucan transport via ABC system (periplasm) |
| R_14GLUCANtexi | 1,4-alpha-D-glucan transport via diffusion (extracellular to periplasm) irreversible |
| R_23CAMPtex | 23cAMP transport via diffusion (extracellular to periplasm) |
| R_23CCMPtex | 23cCMP transport via diffusion (extracellular to periplasm) |
| R_23CGMPtex | 23cGMP transport via diffusion (extracellular to periplasm) |
| R_23CUMPtex | 23cUMP transport via diffusion (extracellular to periplasm) |
| R_23DAPPAt2pp | 2,3-diaminopropionate transport in via proton symport |
| R_23DAPPAtex | 2,3-diaminopropionate transport via diffusion |
| R_23PDE2pp | 2',3'-cyclic-nucleotide phosphodiesterase (UMP) (periplasm) |
| R_23PDE4pp | 2',3'-cyclic-nucleotide phosphodiesterase (CMP) (periplasm) |
| R_23PDE7pp | 2',3'-cyclic-nucleotide phosphodiesterase (AMP) (periplasm) |
| R_23PDE9pp | 2',3'-cyclic-nucleotide phosphodiesterase (GMP) (periplasm) |
| R_26DAHtex | meso-2,6-Diaminoheptanedioate transport via diffusion (extracellular to periplasm) |
| R_2AGPA120tipp | 2-Acyl-sn-glycero-3-phosphatidate (n-C12:0) transporter via facilitated diffusion (periplasm) |
| R_2AGPA140tipp | 2-Acyl-sn-glycero-3-phosphatidate (n-C14:0) transporter via facilitated diffusion (periplasm) |
| R_2AGPA141tipp | 2-Acyl-sn-glycero-3-phosphatidate (n-C14:1) transporter via facilitated diffusion (periplasm) |
| R_2AGPA160tipp | 2-Acyl-sn-glycero-3-phosphatidate (n-C16:0) transporter via facilitated diffusion (periplasm) |
| R_2AGPA161tipp | 2-Acyl-sn-glycero-3-phosphatidate (n-C16:1) transporter via facilitated diffusion (periplasm) |
| R_2AGPA180tipp | 2-Acyl-sn-glycero-3-phosphatidate (n-C18:0) transporter via facilitated diffusion (periplasm) |
| R_2AGPA181tipp | 2-Acyl-sn-glycero-3-phosphatidate (n-C18:1) transporter via facilitated diffusion (periplasm) |
| R_2AGPE120tipp | 2-Acyl-sn-glycero-3-phosphoethanolamine (n-C12:0) transporter via facilitated diffusion (periplasm) |
| R_2AGPE140tipp | 2-Acyl-sn-glycero-3-phosphoethanolamine (n-C14:0) transporter via facilitated diffusion (periplasm) |
| R_2AGPE141tipp | 2-Acyl-sn-glycero-3-phosphoethanolamine (n-C14:1) transporter via facilitated diffusion (periplasm) |
| R_2AGPE160tipp | 2-Acyl-sn-glycero-3-phosphoethanolamine (n-C16:0) transporter via facilitated diffusion (periplasm) |
| R_2AGPE161tipp | 2-Acyl-sn-glycero-3-phosphoethanolamine (n-C16:1) transporter via facilitated diffusion (periplasm) |
| R_2AGPE180tipp | 2-Acyl-sn-glycero-3-phosphoethanolamine (n-C18:0) transporter via facilitated diffusion (periplasm) |
| R_2AGPE181tipp | 2-Acyl-sn-glycero-3-phosphoethanolamine (n-C18:1) transporter via facilitated diffusion (periplasm) |
| R_2AGPEAT120 | 2-acyl-glycerophospho-ethanolamine acyltransferase (n-C12:0) |
| R_2AGPEAT140 | 2-acyl-glycerophospho-ethanolamine acyltransferase (n-C14:0) |
| R_2AGPEAT141 | 2-acyl-glycerophospho-ethanolamine acyltransferase (n-C14:1) |
| R_2AGPEAT160 | 2-acyl-glycerophospho-ethanolamine acyltransferase (n-C16:0) |
| R_2AGPEAT161 | 2-acyl-glycerophospho-ethanolamine acyltransferase (n-C16:1) |
| R_2AGPEAT180 | 2-acyl-glycerophospho-ethanolamine acyltransferase (n-C18:0) |
| R_2AGPEAT181 | 2-acyl-glycerophospho-ethanolamine acyltransferase (n-C18:1) |
| R_2AGPG120tipp | 2-Acyl-sn-glycero-3-phosphoglycerol (n-C12:0) transporter via facilitated diffusion (periplasm) |
| R_2AGPG140tipp | 2-Acyl-sn-glycero-3-phosphoglycerol (n-C14:0) transporter via facilitated diffusion (periplasm) |
| R_2AGPG141tipp | 2-Acyl-sn-glycero-3-phosphoglycerol (n-C14:1) transporter via facilitated diffusion (periplasm) |
| R_2AGPG160tipp | 2-Acyl-sn-glycero-3-phosphoglycerol (n-C16:0) transporter via facilitated diffusion (periplasm) |
| R_2AGPG161tipp | 2-Acyl-sn-glycero-3-phosphoglycerol (n-C16:1) transporter via facilitated diffusion (periplasm) |
| R_2AGPG180tipp | 2-Acyl-sn-glycero-3-phosphoglycerol (n-C18:0) transporter via facilitated diffusion (periplasm) |
| R_2AGPG181tipp | 2-Acyl-sn-glycero-3-phosphoglycerol (n-C18:1) transporter via facilitated diffusion (periplasm) |
| R_2AGPGAT120 | 2-acyl-glycerophospho-glycerol acyltransferase (n-C12:0) |
| R_2AGPGAT140 | 2-acyl-glycerophospho-glycerol acyltransferase (n-C14:0) |
| R_2AGPGAT141 | 2-acyl-glycerophospho-glycerol acyltransferase (n-C14:1) |
| R_2AGPGAT160 | 2-acyl-glycerophospho-glycerol acyltransferase (n-C16:0) |
| R_2AGPGAT161 | 2-acyl-glycerophospho-glycerol acyltransferase (n-C16:1) |
| R_2AGPGAT180 | 2-acyl-glycerophospho-glycerol acyltransferase (n-C18:0) |
| R_2AGPGAT181 | 2-acyl-glycerophospho-glycerol acyltransferase (n-C18:1) |
| R_2DGULRGx | 2-Dehydro-L-gulonate reductase to gluconate (NADH) |
| R_2DGULRGy | 2-Dehydro-L-gulonate reductase to gluconate (NADPH) |
| R_2DGULRx | 2-dehydro-L-gulonate reductase (NADH) |
| R_2DGULRy | 2-dehydro-L-gulonate reductase (NADPH) |
| R_2MAHMP | 2-Methyl-4-amino-5-hydroxymethylpyrimidine diphosphatase |
| R_34dhpactex | dihydroxyphenylacetaldehyde transport via diffusion (extracellular to periplasm) |
| R_3AMACHYD | 3-aminoacrylate hydrolase |
| R_3AMPtex | 3AMP transport via diffusion (extracellular to periplasm) |
| R_3CMPtex | 3CMP transport via diffusion (extracellular to periplasm) |
| R_3GMPtex | 3GMP transport via diffusion (extracellular to periplasm) |
| R_3HAD100 | 3-hydroxyacyl-[acyl-carrier-protein] dehydratase (n-C10:0) |
| R_3HAD120 | 3-hydroxyacyl-[acyl-carrier-protein] dehydratase (n-C12:0) |
| R_3HAD121 | 3-hydroxyacyl-[acyl-carrier-protein] dehydratase (n-C12:1) |
| R_3HAD140 | 3-hydroxyacyl-[acyl-carrier-protein] dehydratase (n-C14:0) |
| R_3HAD141 | 3-hydroxyacyl-[acyl-carrier-protein] dehydratase (n-C14:1) |
| R_3HAD160 | 3-hydroxyacyl-[acyl-carrier-protein] dehydratase (n-C16:0) |
| R_3HAD161 | 3-hydroxyacyl-[acyl-carrier-protein] dehydratase (n-C16:1) |
| R_3HAD180 | 3-hydroxyacyl-[acyl-carrier-protein] dehydratase (n-C18:0) |
| R_3HAD181 | 3-hydroxyacyl-[acyl-carrier-protein] dehydratase (n-C18:1) |
| R_3HAD40 | 3-hydroxyacyl-[acyl-carrier-protein] dehydratase (n-C4:0) |
| R_3HAD60 | 3-hydroxyacyl-[acyl-carrier-protein] dehydratase (n-C6:0) |
| R_3HAD80 | 3-hydroxyacyl-[acyl-carrier-protein] dehydratase (n-C8:0) |
| R_3HCINNMH | 3-hydroxycinnamate hydroxylase |
| R_3HPPPNH | 3-(3-hydroxy-phenyl)propionate hydroxylase |
| R_3HPPtex | 3-hydroxypropionate transport via diffusion (extracellular to periplasm) |
| R_3HPPtpp | 3-hydroxypropionate transport via proton symport (periplasm) |
| R_3KGK | 3-keto-L-gulonate kinase |
| R_3NTD2pp | 3'-nucleotidase (UMP) (periplasm) |
| R_3NTD4pp | 3'-nucleotidase (CMP) (periplasm) |
| R_3NTD7pp | 3'-nucleotidase (AMP) (periplasm) |
| R_3NTD9pp | 3'-nucleotidase (GMP) (periplasm) |
| R_3OAR100 | 3-oxoacyl-[acyl-carrier-protein] reductase (n-C10:0) |
| R_3OAR120 | 3-oxoacyl-[acyl-carrier-protein] reductase (n-C12:0) |
| R_3OAR121 | 3-oxoacyl-[acyl-carrier-protein] reductase (n-C12:1) |
| R_3OAR140 | 3-oxoacyl-[acyl-carrier-protein] reductase (n-C14:0) |
| R_3OAR141 | 3-oxoacyl-[acyl-carrier-protein] reductase (n-C14:1) |
| R_3OAR160 | 3-oxoacyl-[acyl-carrier-protein] reductase (n-C16:0) |
| R_3OAR161 | 3-oxoacyl-[acyl-carrier-protein] reductase (n-C16:1) |
| R_3OAR180 | 3-oxoacyl-[acyl-carrier-protein] reductase (n-C18:0) |
| R_3OAR181 | 3-oxoacyl-[acyl-carrier-protein] reductase (n-C18:1) |
| R_3OAR40 | 3-oxoacyl-[acyl-carrier-protein] reductase (n-C4:0) |
| R_3OAR60 | 3-oxoacyl-[acyl-carrier-protein] reductase (n-C6:0) |
| R_3OAR80 | 3-oxoacyl-[acyl-carrier-protein] reductase (n-C8:0) |
| R_3OAS100 | 3-oxoacyl-[acyl-carrier-protein] synthase (n-C10:0) |
| R_3OAS120 | 3-oxoacyl-[acyl-carrier-protein] synthase (n-C12:0) |
| R_3OAS121 | 3-oxoacyl-[acyl-carrier-protein] synthase (n-C12:1) |
| R_3OAS140 | 3-oxoacyl-[acyl-carrier-protein] synthase (n-C14:0) |
| R_3OAS141 | 3-oxoacyl-[acyl-carrier-protein] synthase (n-C14:1) |
| R_3OAS160 | 3-oxoacyl-[acyl-carrier-protein] synthase (n-C16:0) |
| R_3OAS161 | 3-oxoacyl-[acyl-carrier-protein] synthase (n-C16:1) |
| R_3OAS180 | 3-oxoacyl-[acyl-carrier-protein] synthase (n-C18:0) |
| R_3OAS181 | 3-oxoacyl-[acyl-carrier-protein] synthase (n-C18:1) |
| R_3OAS60 | 3-oxoacyl-[acyl-carrier-protein] synthase (n-C6:0) |
| R_3OAS80 | 3-oxoacyl-[acyl-carrier-protein] synthase (n-C8:0) |
| R_3OXCOAT | 3-oxoadipyl-CoA thiolase |
| R_3PEPTabcpp | tripeptide (LalaDgluMdap) transport via ABC system (periplasm) |
| R_3PEPTtex | LalaDgluMdap (tripeptide) transport via diffusion (extracellular to periplasm) |
| R_3UMPtex | 3UMP transport via diffusion (extracellular to periplasm) |
| R_42A12BOOXpp | 4-(2-Aminoethyl)-1,2-benzenediol:oxygen oxidoreductase(deaminating)(flavin-containing) |
| R_4HOXPACDtex | 4-hydroxyphenylacetaldehyde transport via diffusion (extracellular to periplasm) |
| R_4HTHRS | 4-Hydroxy-L-threonine synthase |
| R_4PCP | tetrapeptide L,D-carboxypeptidase |
| R_4PCPpp | tetrapeptide L,D-carboxypeptidase (periplasm) |
| R_4PEPTabcpp | tetrapeptide (LalaDgluMdapDala) transport via ABC system (periplasm) |
| R_4PEPTtex | LalaDgluMdapDala (pentapeptide) transport via diffusion (extracellular to periplasm) |
| R_5DGLCNR | 5-dehydro-D-gluconate reductase |
| R_5DGLCNt2rpp | 5-Dehydro-D-gluconate transport via proton symport, reversible (periplasm) |
| R_5DGLCNtex | 5-Dehydro-D-gluconate transport via diffusion (extracellular to periplasm) |
| R_5DOAN | 5'-deoxyadenosine nuclosidase |
| R_5MTRtex | 5-Methylthio-D-ribose transport via proton diffusion (extracellular to periplasm) |
| R_5MTRtpp | 5-Methylthio-D-ribose transport via proton symport (periplasm) |
| R_A5PISO | arabinose-5-phosphate isomerase |
| R_AACPS1 | acyl-[acyl-carrier-protein] synthetase (n-C14:0) |
| R_AACPS2 | acyl-[acyl-carrier-protein] synthetase (n-C14:1) |
| R_AACPS3 | acyl-[acyl-carrier-protein] synthetase (n-C16:0) |
| R_AACPS4 | acyl-[acyl-carrier-protein] synthetase (n-C16:1) |
| R_AACPS5 | acyl-[acyl-carrier-protein] synthetase (n-C18:1) |
| R_AACPS6 | acyl-[acyl-carrier-protein] synthetase (n-C18:0) |
| R_AACPS7 | acyl-[acyl-carrier-protein] synthetase (n-C12:0) |
| R_AACPS8 | acyl-[acyl-carrier-protein] synthetase (n-C10:0) |
| R_AACPS9 | acyl-[acyl-carrier-protein] synthetase (n-C8:0) |
| R_AACTOOR | Aminoacetone:oxygen oxidoreductase(deaminating)(flavin-containing) |
| R_AADDGT | dTDP-N-4-acetamido-4,6-dideoxy-D-galactose transferase |
| R_AAMYL | alpha-amylase |
| R_AAMYLpp | alpha-amylase (periplasm) |
| R_AB6PGH | Arbutin 6-phosphate glucohydrolase |
| R_ABTA | 4-aminobutyrate transaminase |
| R_ABUTD | Aminobutyraldehyde dehydrogenase |
| R_ABUTt2pp | 4-aminobutyrate transport in via proton symport (periplasm) |
| R_ABUTtex | 4-aminobutyrate transport via diffusion (extracellular to periplasm) |
| R_ACACCT | acetyl-CoA:acetoacetyl-CoA transferase |
| R_ACACT1r | acetyl-CoA C-acetyltransferase |
| R_ACACT2r | acetyl-CoA C-acyltransferase (butanoyl-CoA) (r) |
| R_ACACT3r | acetyl-CoA C-acyltransferase (hexanoyl-CoA) (r) |
| R_ACACT4r | acetyl-CoA C-acyltransferase (octanoyl-CoA) (r) |
| R_ACACT5r | acetyl-CoA C-acyltransferase (decanoyl-CoA) (r) |
| R_ACACT6r | acetyl-CoA C-acyltransferase (dodecanoyl-CoA) (r) |
| R_ACACT7r | acetyl-CoA C-acyltransferase (tetradecanoyl-CoA) (r) |
| R_ACACT8r | acetyl-CoA acyltransferase (hexadecanoyl-CoA), (r) |
| R_ACACt2pp | acetoacetate transport via proton symport (periplasm) |
| R_ACACtex | acetoacetate transport via diffusion (extracellular to periplasm) |
| R_ACALD | acetaldehyde dehydrogenase (acetylating) |
| R_ACALDtex | acetaldehyde transport via diffusion (extracellular to periplasm) |
| R_ACALDtpp | acetaldehyde reversible transport (periplasm) |
| R_ACANTHAT | acetyl-CoA:anthranilate acetyltransferase |
| R_ACBIPGT | Adenosyl cobinamide phosphate guanyltransferase |
| R_ACCOAC | acetyl-CoA carboxylase |
| R_ACCOAL | acetate-CoA ligase (ADP-forming) |
| R_ACGAL1PPpp | N-acetyl-D-galactosamine 1-phosphatase (periplasm) |
| R_ACGAL1Ptex | N-acetyl-D-galactosamine 1-phosphate transport via diffusion (extracellular to periplasm) |
| R_ACGALtex | N-acetyl-D-galactosamine transport via diffusion (extracellular to periplasm) |
| R_ACGAM1PPpp | N-acetyl-D-glucosamine 1-phosphatase (periplasm) |
| R_ACGAM1Ptex | N-acetyl-D-glucosamine 1-phosphate transport via diffusion (extracellular to periplasm) |
| R_ACGAMK | N-acetylglucosamine kinase |
| R_ACGAMT | UDP-N-acetylglucosamine:undecaprenylphosphate N-acetylglucosamine -1-phosphate transferase |
| R_ACGAptspp | N-Acetyl-D-glucosamine transport via PEP:Pyr PTS (periplasm) |
| R_ACGAtex | N-Acetyl-D-glucosamine transport via diffusion (extracellular to periplasm) |
| R_ACGK | acetylglutamate kinase |
| R_ACGS | N-acetylglutamate synthase |
| R_ACHBS | 2-aceto-2-hydroxybutanoate synthase |
| R_ACKr | acetate kinase |
| R_ACLS | acetolactate synthase |
| R_ACM6PH | N-acetylmuramate 6-phosphate hydrolase |
| R_ACMAMUT | UDP-N-acetyl-D-mannosaminuronic acid transferase |
| R_ACMANAptspp | N-acetyl-D-mannosamine transport via PTS (periplasm) |
| R_ACMANAtex | N-acetyl-D-mannosamine transport via diffusion (extracellular to periplasm) |
| R_ACMUMptspp | N-acetylmuramate transport via PEP:Pyr PTS (periplasm) |
| R_ACMUMtex | N-acetylmuramate transport via diffusion (extracellular to periplasm) |
| R_ACNAMt2pp | N-acetylneuraminate proton symport (periplasm) |
| R_ACNAMtex | N-acetylneuraminate transport via diffusion (extracellular to periplasm) |
| R_ACNML | N-Acetylneuraminate lyase |
| R_ACOAD1f | acyl-CoA dehydrogenase (butanoyl-CoA) |
| R_ACOAD2f | acyl-CoA dehydrogenase (hexanoyl-CoA) |
| R_ACOAD3f | acyl-CoA dehydrogenase (octanoyl-CoA) |
| R_ACOAD4f | acyl-CoA dehydrogenase (decanoyl-CoA) |
| R_ACOAD5f | acyl-CoA dehydrogenase (dodecanoyl-CoA) |
| R_ACOAD6f | acyl-CoA dehydrogenase (tetradecanoyl-CoA) |
| R_ACOAD7f | acyl-CoA dehydrogenase (hexadecanoyl-CoA) |
| R_ACOAD8f | acyl-CoA dehydrogenase (octadecanoyl-CoA) |
| R_ACOATA | Acetyl-CoA ACP transacylase |
| R_ACODA | acetylornithine deacetylase |
| R_ACOLIPAabctex | arabinose modified core oligosaccharide lipid A transport via ABC system (periplasm to extracellular) |
| R_ACONIs | aconitate isomerase (spontaneous) |
| R_ACONMT | Trans-aconitate methyltransferase |
| R_ACONTa | aconitase (half-reaction A, Citrate hydro-lyase) |
| R_ACONTb | aconitase (half-reaction B, Isocitrate hydro-lyase) |
| R_ACOTA | acetylornithine transaminase |
| R_ACPPAT120 | acyl-(acyl carrier protein):phosphate acyltransferase (C12:0) |
| R_ACPPAT140 | acyl-(acyl carrier protein):phosphate acyltransferase (C14:0) |
| R_ACPPAT141 | acyl-(acyl carrier protein):phosphate acyltransferase (C14:1) |
| R_ACPPAT160 | acyl-(acyl carrier protein):phosphate acyltransferase (C16:0) |
| R_ACPPAT161 | acyl-(acyl carrier protein):phosphate acyltransferase (C16:1) |
| R_ACPPAT180 | acyl-(acyl carrier protein):phosphate acyltransferase (C18:0) |
| R_ACPPAT181 | acyl-(acyl carrier protein):phosphate acyltransferase (C18:1) |
| R_ACPS1 | acyl-carrier protein synthase |
| R_ACS | acetyl-CoA synthetase |
| R_ACSERtex | O-Acetyl-L-serine transport via diffusion (extracellular to periplasm) |
| R_ACSERtpp | O-Acetyl-L-serine export via facilitated transport |
| R_ACt2rpp | acetate reversible transport via proton symport (periplasm) |
| R_ACt4pp | Na+/Acetate symport (periplasm) |
| R_ACtex | Acetate transport via diffusion (extracellular to periplasm) |
| R_ADA | Adenosine deaminase |
| R_ADCL | 4-aminobenzoate synthase |
| R_ADCS | 4-amino-4-deoxychorismate synthase |
| R_ADD | adenine deaminase |
| R_ADEt2rpp | adenine transport via proton symport (reversible) (periplasm) |
| R_ADEtex | adenine transport via diffusion (extracellular to periplasm) |
| R_ADK1 | adenylate kinase |
| R_ADK3 | adentylate kinase (GTP) |
| R_ADK4 | adentylate kinase (ITP) |
| R_ADMDC | adenosylmethionine decarboxylase |
| R_ADNCYC | adenylate cyclase |
| R_ADNK1 | adenosine kinase |
| R_ADNUC | adenosine hydrolase |
| R_ADNt2pp | adenosine transport in via proton symport (periplasm) |
| R_ADNt2rpp | adenosine transport in via proton symport, reversible (periplasm) |
| R_ADNtex | adenosine transport via diffusion (extracellular to periplasm) |
| R_ADOCBIK | Adenosyl cobinamide kinase |
| R_ADOCBLS | Adenosylcobalamin 5'-phosphate synthase |
| R_ADOCBLabcpp | Adenosylcobalamin transport via ABC system (periplasm) |
| R_ADOCBLtonex | Adenosylcobalimin transport via ton system (extermal) |
| R_ADPRDP | ADPribose diphosphatase |
| R_ADPT | adenine phosphoribosyltransferase |
| R_ADSK | adenylyl-sulfate kinase |
| R_ADSL1r | adenylsuccinate lyase |
| R_ADSL2r | adenylosuccinate lyase |
| R_ADSS | adenylosuccinate synthase |
| R_AGDC | N-acetylglucosamine-6-phosphate deacetylase |
| R_AGM3PA | N-Acetyl-D-glucosamine(anhydrous)N-Acetylmuramyl-tripeptide amidase |
| R_AGM3PApp | N-Acetyl-D-glucosamine(anhydrous)N-Acetylmuramyl-tripeptide amidase (periplasm) |
| R_AGM3PH | N-Acetyl-D-glucosamine(anhydrous)N-Acetylmuramyl-tripeptide beta -1,4-N-acetylglucosaminidase |
| R_AGM3Pt2pp | GlcNAc-anhMurNAc tripeptide transport in via proton symport (periplasm) |
| R_AGM4PA | N-Acetyl-D-glucosamine(anhydrous)N-Acetylmuramyl-tetrapeptide amidase |
| R_AGM4PApp | N-Acetyl-D-glucosamine(anhydrous)N-Acetylmuramyl-tetrapeptide amidase (periplasm) |
| R_AGM4PCP | N-Acetyl-D-glucosamine(anhydrous)N-Acetylmuramyl-tetrapeptide L,D-carboxypeptidase |
| R_AGM4PCPpp | N-Acetyl-D-glucosamine(anhydrous)N-Acetylmuramyl-tetrapeptide L,D-carboxypeptidase (periplasmic) |
| R_AGM4PH | N-Acetyl-D-glucosamine(anhydrous)N-Acetylmuramyl-tetrapeptide beta -1,4-N-acetylglucosaminidase |
| R_AGM4Pt2pp | GlcNAc-anhMurNAc tetrapeptide transport in via proton symport (periplasm) |
| R_AGMH | N-Acetyl-D-glucosamine(anhydrous)N-Acetylmuramyl beta -1,4-N-acetylglucosaminidase |
| R_AGMHE | ADP-D-glycero-D-manno-heptose epimerase |
| R_AGMT | agmatinase |
| R_AGMt2pp | GlcNAc-anhMurNAc transport in via proton symport (periplasm) |
| R_AGMtex | agmatine transport via diffusion (extracellular to periplasm) |
| R_AGPAT120 | 1-tetradecanoyl-sn-glycerol 3-phosphate O-acyltransferase (n-C12:0) |
| R_AGPAT140 | 1-tetradecanoyl-sn-glycerol 3-phosphate O-acyltransferase (n-C14:0) |
| R_AGPAT141 | 1-tetradec-7-enoyl-sn-glycerol 3-phosphate O-acyltransferase (n-C14:1) |
| R_AGPAT160 | 1-hexadecanoyl-sn-glycerol 3-phosphate O-acyltransferase (n-C16:0) |
| R_AGPAT161 | 1-hexadec-7-enoyl-sn-glycerol 3-phosphate O-acyltransferase (n-C16:1) |
| R_AGPAT180 | 1-octadecanoyl-sn-glycerol 3-phosphate O-acyltransferase (n-C18:0) |
| R_AGPAT181 | 1-octadec-7-enoyl-sn-glycerol 3-phosphate O-acyltransferase (n-C18:1) |
| R_AGPR | N-acetyl-g-glutamyl-phosphate reductase |
| R_AGt3 | silver transport out via proton antiport |
| R_AHCYSNS | S-adenosylhomocysteine nucleosidase |
| R_AICART | phosphoribosylaminoimidazolecarboxamide formyltransferase |
| R_AIRC2 | phosphoribosylaminoimidazole carboxylase |
| R_AIRC3 | phosphoribosylaminoimidazole carboxylase (mutase rxn) |
| R_AKGDH | 2-Oxogluterate dehydrogenase |
| R_AKGt2rpp | 2-oxoglutarate reversible transport via symport (periplasm) |
| R_AKGtex | alpha-ketoglutarate transport via diffusion (extracellular to periplasm) |
| R_ALAALAD | D-alanine-D-alanine dipeptidase |
| R_ALAALAabcpp | D-alanyl-D-alanine (DalaDala) transport via ABC system (periplasm) |
| R_ALAALAr | D-alanine-D-alanine ligase (reversible) |
| R_ALAALAtex | D-alanyl-D-alanine (DalaDala) transport via diffusion (extracellular to periplasm) |
| R_ALAGLUE | L-alanyl-gamma-glutamate epimerase |
| R_ALAR | alanine racemase |
| R_ALATA_D2 | D-alanine transaminase |
| R_ALATA_L | L-alanine transaminase |
| R_ALATA_L2 | alanine transaminase |
| R_ALATRS | Alanyl-tRNA synthetase |
| R_ALAabcpp | L-alanine transport via ABC system (periplasm) |
| R_ALAt2pp | L-alanine transport in via proton symport (periplasm) |
| R_ALAt2rpp | L-alanine reversible transport via proton symport (periplasm) |
| R_ALAt4pp | L-alanine transport in via sodium symport (periplasm) |
| R_ALAtex | L-alanine transport via diffusion (extracellular to periplasm) |
| R_ALCD19 | alcohol dehydrogenase (glycerol) |
| R_ALCD2x | alcohol dehydrogenase (ethanol) |
| R_ALDD19xr | aldehyde dehydrogenase (phenylacetaldehyde, NAD) |
| R_ALDD2x | aldehyde dehydrogenase (acetaldehyde, NAD) |
| R_ALDD2y | aldehyde dehydrogenase (acetaldehyde, NADP) |
| R_ALDD3y | aldehyde dehydrogenase (propanal, NADP) |
| R_ALDD4 | aldehyde dehydrogenase (butanal, NAD) |
| R_ALLK | Allose kinase |
| R_ALLPI | Allose 6-phosphate isomerase |
| R_ALLTAMH | allantoate amidohydrolase |
| R_ALLTN | allantoinase |
| R_ALLTNt2rpp | allantoin transport in via proton symport (periplasm) |
| R_ALLTNtex | allantoin transport via diffusion (extracellular to periplasm) |
| R_ALLULPE | Allulose 6-phosphate epimerase |
| R_ALLabcpp | D-allose transport via ABC system (periplasm) |
| R_ALLtex | Allose transport via diffusion (extracellular to periplasm) |
| R_ALPATE160pp | apolipoprotein N-acyltransferase (phosphatidylethanolamine, periplasm) |
| R_ALPATG160pp | apolipoprotein N-acyltransferase (phosphatidylglycerol, periplasm) |
| R_ALR2 | aldose reductase (methylglyoxal) |
| R_ALR4x | aldose reductase (acetol) |
| R_ALTRH | altronate hydrolase |
| R_AM3PA | anhydrous-N-Acetylmuramyl-tripeptide amidase |
| R_AM4PA | anhydrous-N-Acetylmuramyl-tetrapeptide amidase |
| R_AM4PCP | anhydrous-N-Acetylmuramyl-tetrapeptide L,D-carboxypeptidase |
| R_AMALT1 | Amylomaltase (maltotriose) |
| R_AMALT2 | Amylomaltase (maltotetraose) |
| R_AMALT3 | Amylomaltase (maltopentaose) |
| R_AMALT4 | Amylomaltase (maltohexaose) |
| R_AMANAPEr | N-acetylmannosamine 6-phosphate epimerase |
| R_AMANK | N-acetyl-D-mannosamine kinase |
| R_AMAOTr | adenosylmethionine-8-amino-7-oxononanoate transaminase |
| R_AMMQLT8 | S-adenosylmethione:2-demthylmenaquinole methyltransferase (menaquinone 8) |
| R_AMPMS2 | 4-amino-2-methyl-5-phosphomethylpyrimidine synthetase |
| R_AMPN | AMP nucleosidase |
| R_AMPTASECG | alanyl aminopeptidase (cys-gly) |
| R_AMPTASEPG | aminopeptidase (pro-gly) |
| R_AMPtex | AMP transport via diffusion (extracellular to periplasm) |
| R_ANHGMtex | GlcNAc-anhMurNAc transport via diffusion (extracellular to periplasm) |
| R_ANHMK | 1,6-anhydrous-N-Acetylmuramate kinase |
| R_ANPRT | anthranilate phosphoribosyltransferase |
| R_ANS | anthranilate synthase |
| R_AOBUTDs | L-2-amino-3-oxobutanoate decarboxylation (spontaneous) |
| R_AOXSr2 | 8-amino-7-oxononanoate synthase |
| R_AP4AH | Ap4A hydrolase |
| R_AP4AS | Ap4A synthetase |
| R_AP5AH | Ap5A hydrolase |
| R_APCS | aminopropylcadaverine synthase |
| R_APG3PAT120 | acyl-phosphate:glycerol-3-phosphate acyltransferase (C12:0) |
| R_APG3PAT140 | acyl-phosphate:glycerol-3-phosphate acyltransferase (C14:0) |
| R_APG3PAT141 | acyl-phosphate:glycerol-3-phosphate acyltransferase (C14:1) |
| R_APG3PAT160 | acyl-phosphate:glycerol-3-phosphate acyltransferase (C16:0) |
| R_APG3PAT161 | acyl-phosphate:glycerol-3-phosphate acyltransferase (C16:1) |
| R_APG3PAT180 | acyl-phosphate:glycerol-3-phosphate acyltransferase (C18:0) |
| R_APG3PAT181 | acyl-phosphate:glycerol-3-phosphate acyltransferase (C18:1) |
| R_APH120 | acylphosphatase (C12:0) |
| R_APH140 | acylphosphatase (C14:0) |
| R_APH141 | acylphosphatase (C14:1) |
| R_APH160 | acylphosphatase (C16:0) |
| R_APH161 | acylphosphatase (C16:1) |
| R_APH180 | acylphosphatase (C18:0) |
| R_APH181 | acylphosphatase (C18:1) |
| R_APPLDHr | aminopropanol dehydrogenase reversible |
| R_APRAUR | 5-amino-6-(5-phosphoribosylamino)uracil reductase |
| R_ARAI | L-arabinose isomerase |
| R_ARBTNR1 | aerobactin reductase |
| R_ARBTNR2 | aerobactin reductase |
| R_ARBTNR3 | aerobactin reductase |
| R_ARBTNabcpp | aerobactin transport via ABC system (periplasm) |
| R_ARBTNexs | aerobactin Fe-loading reaction (spontaneous) |
| R_ARBTNtex | aerobactin secretion (to extracellular) |
| R_ARBTNtonex | aerobactin transport via ton system (extracellular) |
| R_ARBTNtpp | aerobactin secretion (to periplasm) |
| R_ARBTptspp | arbutin transport via PEP:Pyr PTS (periplasm) |
| R_ARBTtex | arbutin transport via diffusion (extracellular to periplasm) |
| R_ARBabcpp | L-arabinose transport via ABC system (periplasm) |
| R_ARBt2rpp | L-arabinose transport via proton symport (periplasm) |
| R_ARBt3ipp | L-arabinose transport via proton antiport (periplasm) |
| R_ARBtex | L-arabinose transport via diffusion (extracellular to periplasm) |
| R_ARGAGMt7pp | Arginine/agmatine antiport (periplasm) |
| R_ARGDC | arginine decarboxylase |
| R_ARGDCpp | arginine decarboxylase |
| R_ARGORNt7pp | arginine/ornithine antiporter (periplasm) |
| R_ARGSL | argininosuccinate lyase |
| R_ARGSS | argininosuccinate synthase |
| R_ARGTRS | Arginyl-tRNA synthetase |
| R_ARGabcpp | L-arginine transport via ABC system (periplasm) |
| R_ARGt3pp | L-arginine transport out via proton antiport (cytoplasm to periplasm) |
| R_ARGtex | L-arginine transport via diffusion (extracellular to periplasm) |
| R_ASAD | aspartate-semialdehyde dehydrogenase |
| R_ASCBPL | L-ascorbate 6-phosphate lactonase |
| R_ASCBptspp | L-ascorbate transport via PEP:Pyr PTS (periplasm) |
| R_ASCBtex | L-ascorbate transport via diffusion (extracellular to periplasm) |
| R_ASNN | L-asparaginase |
| R_ASNNpp | L-asparaginase |
| R_ASNS1 | asparagine synthase (glutamine-hydrolysing) |
| R_ASNS2 | asparagine synthetase |
| R_ASNTRS | Asparaginyl-tRNA synthetase |
| R_ASNabcpp | L-asparagine transport via ABC system (periplasm) |
| R_ASNt2rpp | L-asparagine reversible transport via proton symport (periplasm) |
| R_ASNtex | L-asparagine transport via diffusion (extracellular to periplasm) |
| R_ASO3t8pp | arsenite efflux via ATP hydrolysis (periplasm) |
| R_ASO3tex | arsenite transport via diffusion (extracellular to periplasm) |
| R_ASP1DC | aspartate 1-decarboxylase |
| R_ASPCT | aspartate carbamoyltransferase |
| R_ASPK | aspartate kinase |
| R_ASPO3 | L-aspartate oxidase |
| R_ASPO4 | L-aspartate oxidase |
| R_ASPO5 | L-aspartate oxidase |
| R_ASPO6 | L-aspartate oxidase |
| R_ASPT | L-aspartase |
| R_ASPTA | aspartate transaminase |
| R_ASPTRS | Aspartyl-tRNA synthetase |
| R_ASPabcpp | L-aspartate transport via ABC system (periplasm) |
| R_ASPt2_2pp | Aspartate transport via proton symport (2 H) (periplasm) |
| R_ASPt2_3pp | L-asparate transport via proton symport (3 H) (periplasm) |
| R_ASPt2pp | L-aspartate transport in via proton symport (periplasm) |
| R_ASPt2rpp | L-aspartate transport in via proton symport (periplasm) reversible |
| R_ASPtex | L-aspartate transport via diffusion (extracellular to periplasm) |
| R_ASR | arsenate reductase |
| R_AST | Arginine succinyltransferase |
| R_ATHRDHr | L-allo-threonine dehydrogenase |
| R_ATPHs | ATP amine hydrolysis (spontaneous) |
| R_ATPM | ATP maintenance requirement |
| R_ATPPRT | ATP phosphoribosyltransferase |
| R_ATPS4rpp | ATP synthase (four protons for one ATP) (periplasm) |
| R_BALAt2pp | beta-alanine transport in via proton symport (periplasm) |
| R_BALAtex | beta-alanine transport via diffusion (extracellular to periplasm) |
| R_BETALDHx | betaine-aldehyde dehydrogenase |
| R_BETALDHy | betaine-aldehyde dehydrogenase |
| R_BMOCOS | bis-molybdenum cofactor synthase |
| R_BMOGDS1 | bis-molybdopterin guanine dinucleotide synthase (single GDP) |
| R_BMOGDS2 | bis-molybdopterin guanine dinucleotide synthase |
| R_BPNT | 3',5'-bisphosphate nucleotidase |
| R_BSORx | Biotin sulfoxide reductase |
| R_BSORy | Biotin sulfoxide reductase |
| R_BTNt2ipp | Biotin transport via proton symport (periplasm) |
| R_BTNtex | Biotin transport via diffusion (extracellular to periplasm) |
| R_BTS5 | Biotin synthase |
| R_BUTCT | Acetyl-CoA:butyrate-CoA transferase |
| R_BUTSO3abcpp | butanesulfonate transport via ABC system (periplasm) |
| R_BUTSO3tex | butanesulfonate transport via diffusion (extracellular to periplasm) |
| R_BUTt2rpp | Butyrate transport via proton symport, reversible (periplasm) |
| R_BUTtex | Butyrate transport via diffusion (extracellular to periplasm) |
| R_BWCOGDS1 | tungsten bispterin guanine dinucleotide synthase (single GDP) |
| R_BWCOGDS2 | tungsten bispterin guanine dinucleotide synthase |
| R_BWCOS | tungsten bispterin cofactor synthase |
| R_CA2t3pp | calcium (Ca+2) transport out via proton antiport (periplasm) |
| R_CA2tex | calcium (Ca+2) transport via diffusion (extracellular to periplasm) |
| R_CADVtpp | Lysine/Cadaverine antiporter (periplasm) |
| R_CAT | catalase |
| R_CAt6pp | calcium / sodium antiporter (1:1) |
| R_CBIAT | Cobinamide adenyltransferase |
| R_CBItonex | Cobinamide transport via ton system (extermal) |
| R_CBIuabcpp | Cobinamide transport via ABC system (uptake, periplasm) |
| R_CBL1abcpp | Cob(1)alamin transport via ABC system (periplasm) |
| R_CBL1tonex | Cob(1)alamin transport via ton system (extermal) |
| R_CBLAT | cob(I)alamin adenosyltransferase |
| R_CBMD | carbamate deaminase |
| R_CBMKr | Carbamate kinase |
| R_CBPS | carbamoyl-phosphate synthase (glutamine-hydrolysing) |
| R_CCGS | 7-cyano-7-carbaguanine synthase |
| R_CD2abcpp | Cadmium (Cd+2) ABC transporter (periplasm) |
| R_CD2t3pp | cadmium (Cd+2) transport out via proton antiport (periplasm) |
| R_CD2tex | cadmium (Cd+2) transport via diffusion (extracellular to periplasm) |
| R_CD2tpp | cadmium (+2) transport in via permease (no H+) |
| R_CDAPPA120 | CDP-Diacylglycerol pyrophostatase (n-C12:0) |
| R_CDAPPA140 | CDP-Diacylglycerol pyrophostatase (n-C14:0) |
| R_CDAPPA141 | CDP-Diacylglycerol pyrophostatase (n-C14:1) |
| R_CDAPPA160 | CDP-Diacylglycerol pyrophostatase (n-C16:0) |
| R_CDAPPA161 | CDP-Diacylglycerol pyrophostatase (n-C16:1) |
| R_CDAPPA180 | CDP-Diacylglycerol pyrophostatase (n-C18:0) |
| R_CDAPPA181 | CDP-Diacylglycerol pyrophostatase (n-C18:1) |
| R_CDGR | 7-cyano-7-deazaguanine reductase |
| R_CDGS | 7-deaza-7-carboxyguanine synthase |
| R_CDPMEK | 4-(cytidine 5'-diphospho)-2-C-methyl-D-erythritol kinase |
| R_CFAS160E | cyclopropane fatty acid synthase (Phosphatidylethanolamine, n-C16:0) |
| R_CFAS160G | cyclopropane fatty acid synthase (Phosphatidylglycerol, n-C16:0) |
| R_CFAS180E | cyclopropane fatty acid synthase (Phosphatidylethanolamine, n-C18:0) |
| R_CFAS180G | cyclopropane fatty acid synthase (Phosphatidylglycerol, n-C18:0) |
| R_CGLYabcpp | L-Cysteinylglycine (Cys-Gly) transport via ABC system (periplasm) |
| R_CGLYtex | L-Cysteinylglycine transport via diffusion (extracellular to periplasm) |
| R_CHLabcpp | choline transport via ABC system (periplasm) |
| R_CHLt2pp | choline transport via proton symport (periplasm) |
| R_CHLtex | choline transport via diffusion (extracellular to periplasm) |
| R_CHOLD | choline dehydrogenase |
| R_CHORM | chorismate mutase |
| R_CHORS | chorismate synthase |
| R_CHRPL | Chorismate pyruvate lyase |
| R_CHTBSptspp | chitobiose transport via PEP:Pyr PTS (periplasm) |
| R_CHTBStex | chitobiose transport via diffusion (extracellular to periplasm) |
| R_CINNDO | Cinnamate dioxygenase |
| R_CITL | Citrate lyase |
| R_CITt3pp | citrate transport out via proton antiport (periplasm) |
| R_CITt7pp | Citrate transport via succinate antiport (periplasm) |
| R_CITtex | citrate transport via diffusion (extracellular to periplasm) |
| R_CLIPAabctex | cold lipid A transport via ABC system (periplasm to extracellular) |
| R_CLPNH120pp | cardiolipin hydrolase (periplasm, n-C12:0) |
| R_CLPNH140pp | cardiolipin hydrolase (periplasm, n-C14:0) |
| R_CLPNH141pp | cardiolipin hydrolase (periplasm, n-C14:1) |
| R_CLPNH160pp | cardiolipin hydrolase (periplasm, n-C16:0) |
| R_CLPNH161pp | cardiolipin hydrolase (periplasm, n-C16:1) |
| R_CLPNH180pp | cardiolipin hydrolase (periplasm, n-C18:0) |
| R_CLPNH181pp | cardiolipin hydrolase (periplasm, n-C18:1) |
| R_CLPNS120pp | cardiolipin synthase (periplasmic, n-C12:0) |
| R_CLPNS140pp | cardiolipin synthase (periplasmic, n-C14:0) |
| R_CLPNS141pp | cardiolipin synthase (periplasmic, n-C14:1) |
| R_CLPNS160pp | cardiolipin synthase (periplasmic, n-C16:0) |
| R_CLPNS161pp | cardiolipin synthase (periplasmic, n-C16:1) |
| R_CLPNS180pp | cardiolipin synthase (periplasmic, n-C18:0) |
| R_CLPNS181pp | cardiolipin synthase (periplasmic, n-C18:1) |
| R_CLt3_2pp | chloride transport out via proton antiport (2:1) (periplasm) |
| R_CLtex | chloride (Cl-1) transport via diffusion (extracellular to periplasm) |
| R_CMPN | CMP nucleosidase |
| R_CMPtex | CMP transport via diffusion (extracellular to periplasm) |
| R_CMtex | Chloramphenicol transport via diffusion (extracellular to periplasm) |
| R_CMtpp | Chloramphenicol transport via TolC system |
| R_CO2tex | CO2 transport via diffusion (extracellular to periplasm) |
| R_CO2tpp | CO2 transporter via diffusion (periplasm) |
| R_COBALT2abcpp | Cobalt (Co+2) ABC transporter (periplasm) |
| R_COBALT2t3pp | cobalt (Co+2) transport out via proton antiport (periplasm) |
| R_COBALT2tex | cobalt (Co+2) transport via diffusion (extracellular to periplasm) |
| R_COBALT2tpp | cobalt transport in via permease (no H+) |
| R_COLIPAKpp | Lipid A core kinase (periplasm) |
| R_COLIPAPabctex | core oligosaccharide lipid A diphosphate transport via ABC system (periplasm to extracellular) |
| R_COLIPAabcpp | core oligosaccharide lipid A transport via ABC system (periplasm) |
| R_COLIPAabctex | core oligosaccharide lipid A transport via ABC system (periplasm to extracellular) |
| R_CPGNR1 | coprogen(Fe(III)) reductase |
| R_CPGNR2 | coprogen(Fe(III)) reductase |
| R_CPGNR3 | coprogen(Fe(III)) reductase |
| R_CPGNUtex | coprogen unloaded secretion (extracellular) |
| R_CPGNUtpp | coprogen unloaded secretion |
| R_CPGNabcpp | coprogen transport via ABC system (periplasm) |
| R_CPGNexs | coprogen Fe-loading reaction (spontaneaous) |
| R_CPGNtonex | Coprogen transport via ton system (extracellular) |
| R_CPH4S | 6-carboxy-5,6,7,8-tetrahydropterin synthase |
| R_CPMPS | cyclic pyranopterin monophosphate synthase |
| R_CPPPGO | coproporphyrinogen oxidase (O2 required) |
| R_CPPPGO2 | Oxygen Independent coproporphyrinogen-III oxidase |
| R_CRNBTCT | gamma-butyrobetainyl-CoA: carnitine CoA transferase |
| R_CRNCAL2 | Carnitine-CoA Ligase |
| R_CRNCAR | carnitine-CoA racemase |
| R_CRNCBCT | crotonobetainyl-CoA: carnitine CoA transferase |
| R_CRNCDH | Carnityl-CoA dehydratse |
| R_CRNDCAL2 | D-Carnitine-CoA Ligase |
| R_CRNDabcpp | D-carnitine transport via ABC system (periplasm) |
| R_CRNDt2rpp | D-carnitine outward transport (H+ antiport) |
| R_CRNDtex | D-carnitine transport via diffusion (extracellular to periplasm) |
| R_CRNabcpp | L-carnitine transport via ABC system (periplasm) |
| R_CRNt2rpp | L-carnitine outward transport (H+ antiport) |
| R_CRNt7pp | Carnitine/butyrobetaine antiporter (periplasm) |
| R_CRNt8pp | L-carnitine/D-carnitine antiporter (periplasm) |
| R_CRNtex | L-carnitine transport via diffusion (extracellular to periplasm) |
| R_CS | citrate synthase |
| R_CSND | Cytosine deaminase |
| R_CSNt2pp | cytosine transport in via proton symport (periplasm) |
| R_CSNtex | cytosine transport via diffusion (extracellular to periplasm) |
| R_CTBTCAL2 | Crotonobetaine-CoA Ligase |
| R_CTBTabcpp | crotonobetaine transport via ABC system (periplasm) |
| R_CTBTt2rpp | cronobetaine outward transport (H+ antiport) |
| R_CTECOAI6 | 3-cis-2-trans-enoyl-CoA isomerase |
| R_CTECOAI7 | 3-cis-2-trans-enoyl-CoA isomerase |
| R_CTECOAI8 | 3-cis-2-trans-enoyl-CoA isomerase |
| R_CTPS2 | CTP synthase (glutamine) |
| R_CU1Opp | Cuprous Oxidase (Cu+1) |
| R_CU1abcpp | Copper (Cu +1) ABC transporter (periplasm) |
| R_CU2abcpp | Copper (Cu+2) ABC transporter (periplasm) |
| R_CU2tex | copper (Cu+2) transport via diffusion (extracellular to periplasm) |
| R_CU2tpp | copper transport in via permease (no H+) |
| R_CUt3 | copper transport out via proton antiport |
| R_CUtex | copper (Cu+1) transport via diffusion (extracellular to periplasm) |
| R_CYANST | Cyanide sulfurtransferase |
| R_CYANSTpp | Cyanide sulfurtransferase (periplasmic) |
| R_CYANtex | Cyanide transport via diffusion (extracellular to periplasm) |
| R_CYNTAH | Cyanate aminohydrolase |
| R_CYNTt2pp | Cyanate transport via proton symport (periplasm) |
| R_CYNTtex | Cyanate transport via diffusion (extracellular to periplasm) |
| R_CYSDDS | D-cysteine desulfhydrase |
| R_CYSDS | Cysteine Desulfhydrase |
| R_CYSDabcpp | D-cysteine uptake via ABC system (periplasm) |
| R_CYSDtex | D-cysteine transport via diffusion (extracellular to periplasm) |
| R_CYSS | cysteine synthase |
| R_CYSSADS | L-cysteine sulfinic acid desulfurase |
| R_CYSTL | cystathionine b-lyase |
| R_CYSTRS | Cysteinyl-tRNA synthetase |
| R_CYSabc2pp | L-cysteine export via ABC system (cytoplasm to periplasm) |
| R_CYSabcpp | L-cysteine uptake via ABC system (periplasm) |
| R_CYStex | L-cysteine transport via diffusion (extracellular to periplasm) |
| R_CYStpp | L-cysteine export via facilitated transport |
| R_CYTBD2pp | cytochrome oxidase bd (menaquinol-8: 2 protons) (periplasm) |
| R_CYTBDpp | cytochrome oxidase bd (ubiquinol-8: 2 protons) (periplasm) |
| R_CYTBO3_4pp | cytochrome oxidase bo3 (ubiquinol-8: 4 protons) (periplasm) |
| R_CYTD | cytidine deaminase |
| R_CYTDH | Cytidine hydrolase |
| R_CYTDK2 | cytidine kinase (GTP) |
| R_CYTDt2pp | cytidine transport in via proton symport (periplasm) |
| R_CYTDt2rpp | cytidine transport in via proton symport, reversible (periplasm) |
| R_CYTDtex | cytidine transport via diffusion (extracellular to periplasm) |
| R_CYTK1 | cytidylate kinase (CMP) |
| R_CYTK2 | cytidylate kinase (dCMP) |
| R_D_DASH_LACt2pp | D-lactate transport via proton symport (periplasm) |
| R_D_DASH_LACtex | D-lactate transport via diffusion (extracellular to periplasm) |
| R_DAAD | D-Amino acid dehydrogenase |
| R_DADA | Deoxyadenosine deaminase |
| R_DADK | deoxyadenylate kinase |
| R_DADNt2pp | deoxyadenosine transport in via proton symport (periplasm) |
| R_DADNtex | deoxyadenosine transport via diffusion (extracellular to periplasm) |
| R_DAGK120 | diacylglycerol kinase (n-C12:0) |
| R_DAGK140 | diacylglycerol kinase (n-C14:0) |
| R_DAGK141 | diacylglycerol kinase (n-C14:1) |
| R_DAGK160 | diacylglycerol kinase (n-C16:0) |
| R_DAGK161 | diacylglycerol kinase (n-C16:1) |
| R_DAGK180 | diacylglycerol kinase (n-C18:0) |
| R_DAGK181 | diacylglycerol kinase (n-C18:1) |
| R_DALAt2pp | D-alanine transport in via proton symport (periplasm) |
| R_DALAtex | D-Alanine transport via diffusion (extracellular to periplasm) |
| R_DAMPtex | dAMP transport via diffusion (extracellular to periplasm) |
| R_DAPAL | 2,3-diaminopropionate amonnia lyase |
| R_DAPDC | diaminopimelate decarboxylase |
| R_DAPE | diaminopimelate epimerase |
| R_DAPabcpp | M-diaminopimelic acid ABC transport (periplasm) |
| R_DAPtex | 1,5-Diaminopentane transport via diffusion (extracellular to periplasm) |
| R_DASYN120 | CDP-diacylglycerol synthetase (n-C12:0) |
| R_DASYN140 | CDP-diacylglycerol synthetase (n-C14:0) |
| R_DASYN141 | CDP-diacylglycerol synthetase (n-C14:1) |
| R_DASYN160 | CDP-diacylglycerol synthetase (n-C16:0) |
| R_DASYN161 | CDP-diacylglycerol synthetase (n-C16:1) |
| R_DASYN180 | CDP-diacylglycerol synthetase (n-C18:0) |
| R_DASYN181 | CDP-diacylglycerol synthetase (n-C18:1) |
| R_DATPHs | dATP amine hydrolysis (spontaneous) |
| R_DB4PS | 3,4-Dihydroxy-2-butanone-4-phosphate synthase |
| R_DBTS | dethiobiotin synthase |
| R_DC6PH | diacetylchitobiose-6-phosphate hydrolase |
| R_DCAtex | Decanoate transport via diffusion (extracellular to periplasm) |
| R_DCMPtex | dCMP transport via diffusion (extracellular to periplasm) |
| R_DCTPD | dCTP deaminase |
| R_DCYTD | deoxycytidine deaminase |
| R_DCYTt2pp | deoxycytidine transport in via proton symport (periplasm) |
| R_DCYTtex | deoxycytidine transport via diffusion (extracellular to periplasm) |
| R_DDCAtexi | Fatty acid (dodecanoate) transport via facilitated irreversible diffusion (extracellular to periplasm) |
| R_DDGALK | 2-dehydro-3-deoxygalactonokinase |
| R_DDGLCNt2rpp | 2-dehydro-3-deoxy-D-gluconate transport via proton symport, reversible (periplasm) |
| R_DDGLCNtex | 2-dehydro-3-deoxy-D-gluconate transport via diffusion (extracellular to periplasm) |
| R_DDGLK | 2-dehydro-3-deoxygluconokinase |
| R_DDPA | 3-deoxy-D-arabino-heptulosonate 7-phosphate synthetase |
| R_DDPGALA | 2-dehydro-3-deoxy-6-phosphogalactonate aldolase |
| R_DGK1 | deoxyguanylate kinase (dGMP:ATP) |
| R_DGMPtex | dGMP transport via diffusion (extracellular to periplasm) |
| R_DGSNt2pp | deoxyguanosine transport in via proton symport (periplasm) |
| R_DGSNtex | deoxyguanosine transport via diffusion (extracellular to periplasm) |
| R_DHACOAH | 2,3-dehydroadipyl-CoA hydratase |
| R_DHAD1 | dihydroxy-acid dehydratase (2,3-dihydroxy-3-methylbutanoate) |
| R_DHAD2 | Dihydroxy-acid dehydratase (2,3-dihydroxy-3-methylpentanoate) |
| R_DHAPT | Dihydroxyacetone phosphotransferase |
| R_DHAtex | Dihydroxyacetone transport via diffusion (extracellular to periplasm) |
| R_DHAtpp | Dihydroxyacetone transport via facilitated diffusion (periplasm) |
| R_DHBD | 2,3-dihydro-2,3-dihydroxybenzoate dehydrogenase |
| R_DHBS | 2,3-dihydroxybenzoate adenylate synthase |
| R_DHBSH | 2,3-dihydroxybenzoylserine hydrolase |
| R_DHCIND | 2,3-dihydroxycinnamate dehydrogenase |
| R_DHCINDO | 2,3-dihydroxycinnamate 1,2-dioxygenase |
| R_DHDPRy | dihydrodipicolinate reductase (NADPH) |
| R_DHDPS | dihydrodipicolinate synthase |
| R_DHFR | dihydrofolate reductase |
| R_DHFS | dihydrofolate synthase |
| R_DHMPTR | Dihydromonapterin reductase |
| R_DHNAOT4 | 1,4-dihydroxy-2-naphthoate octaprenyltransferase |
| R_DHNCOAS | 1,4-dihydroxy-2-napthoyl-CoA synthase |
| R_DHNCOAT | 1,4-dihydroxy-2-napthoyl-CoA thioesterase |
| R_DHNPA2r | dihydroneopterin aldolase reversible |
| R_DHNPTE | Dihydroneopterin epimerase |
| R_DHORD2 | dihydoorotic acid dehydrogenase (quinone8) |
| R_DHORD5 | dihydroorotic acid (menaquinone-8) |
| R_DHORDfum | Fumarate dependent DHORD |
| R_DHORTS | dihydroorotase |
| R_DHPPD | 2,3-dihydroxyphenylpropionate dehydrogenase |
| R_DHPPDA2 | diaminohydroxyphosphoribosylaminopryrimidine deaminase (25drapp) |
| R_DHPS2 | dihydropteroate synthase |
| R_DHPTDCs2 | 4,5-dihydroxy-2,3-pentanedione cyclization (spontaneous) |
| R_DHPTDNR | dihydropteridine reductase |
| R_DHPTDNRN | dihydropteridine reductase (NADH) |
| R_DHPTPE | dihydroneopterin triphosphate 2'-epimerase |
| R_DHQS | 3-dehydroquinate synthase |
| R_DHQTi | 3-dehydroquinate dehydratase, irreversible |
| R_DIMPtex | dIMP transport via diffusion (extracellular to periplasm) |
| R_DINSt2pp | deoxyinosine transport in via proton symport (periplasm) |
| R_DINStex | deoxyinosine transport via diffusion (extracellular to periplasm) |
| R_DKGLCNR1 | 2,5-diketo-D-gluconate reductase |
| R_DKGLCNR2x | 2,5-diketo-D-gluconate reductase (NADH) |
| R_DKGLCNR2y | 2,5-diketo-D-gluconate reductase (NADPH) |
| R_DMATT | dimethylallyltranstransferase |
| R_DMPPS | 1-hydroxy-2-methyl-2-(E)-butenyl 4-diphosphate reductase (dmpp) |
| R_DMQMT | 3-Dimethylubiquinonol 3-methyltransferase |
| R_DMSOR1 | Dimethyl sulfoxide reductase (Menaquinol 8) |
| R_DMSOR1pp | Dimethyl sulfoxide reductase (Menaquinol 8) (periplasm) |
| R_DMSOR2 | Dimethyl sulfoxide reductase (Demethylmenaquinol 8) |
| R_DMSOR2pp | Dimethyl sulfoxide reductase (Demethylmenaquinol 8) (periplasm) |
| R_DMSOtex | Dimethyl sulfoxide transport via diffusion (extracellular to periplasm) |
| R_DMSOtpp | Dimethyl sulfoxide transport via diffusion (periplasm) |
| R_DMStex | Dimethyl sulfide transport via diffusion (extracellular to periplasm) |
| R_DNMPPA | Dihydroneopterin monophosphate dephosphorylase |
| R_DNTPPA | Dihydroneopterin triphosphate pyrophosphatase |
| R_DOGULNR | 2,3 dioxo-L-gulonate reductase |
| R_DOPAtex | dopamine transport via diffusion (extracellular to periplasm) |
| R_DOXRBCNtex | Doxorubicin transport via diffusion (extracellular to periplasm) |
| R_DOXRBCNtpp | Doxorubicin transport via TolC system |
| R_DPCOAK | dephospho-CoA kinase |
| R_DPR | 2-dehydropantoate 2-reductase |
| R_DRPA | deoxyribose-phosphate aldolase |
| R_DSBAO1 | DsbA protein reoxidation reaction (aerobic) |
| R_DSBAO2 | DsbA protein reoxidation reaction (anaerobic) |
| R_DSBCGT | DsbC:glutathione thiotransferase |
| R_DSBDR | DsbD reductase |
| R_DSBGGT | DsbG:glutathione thiotransferase |
| R_DSERDHr | D-serine dehydrogenase |
| R_DSERt2pp | D-serine transport in via proton symport (periplasm) |
| R_DSERtex | D-serine transport via diffusion (extracellular to periplasm) |
| R_DTARTD | D(-)-tartrate dehydratase |
| R_DTMPK | dTMP kinase |
| R_DTMPtex | dTMP transport via diffusion (extracellular to periplasm) |
| R_DUMPtex | dUMP transport via diffusion (extracellular to periplasm) |
| R_DURADx | dihydrouracil dehydrogenase (NAD) |
| R_DURIK1 | deoxyuridine kinase (ATP:Deoxyuridine) |
| R_DURIPP | deoxyuridine phosphorylase |
| R_DURIt2pp | deoxyuridine transport in via proton symport (periplasm) |
| R_DURItex | deoxyuridine transport via diffusion (extracellular to periplasm) |
| R_DUTPDP | dUTP diphosphatase |
| R_DXPRIi | 1-deoxy-D-xylulose reductoisomerase |
| R_DXPS | 1-deoxy-D-xylulose 5-phosphate synthase |
| R_DXYLK | 1-Deoxy-D-xylulose kinase |
| R_E4PD | Erythrose 4-phosphate dehydrogenase |
| R_EAR100x | enoyl-[acyl-carrier-protein] reductase (NADH) (n-C10:0) |
| R_EAR100y | enoyl-[acyl-carrier-protein] reductase (NADPH) (n-C10:0) |
| R_EAR120x | enoyl-[acyl-carrier-protein] reductase (NADH) (n-C12:0) |
| R_EAR120y | enoyl-[acyl-carrier-protein] reductase (NADPH) (n-C12:0) |
| R_EAR121x | enoyl-[acyl-carrier-protein] reductase (NADH) (n-C12:1) |
| R_EAR121y | enoyl-[acyl-carrier-protein] reductase (NADPH) (n-C12:1) |
| R_EAR140x | enoyl-[acyl-carrier-protein] reductase (NADH) (n-C14:0) |
| R_EAR140y | enoyl-[acyl-carrier-protein] reductase (NADPH) (n-C14:0) |
| R_EAR141x | enoyl-[acyl-carrier-protein] reductase (NADH) (n-C14:1) |
| R_EAR141y | enoyl-[acyl-carrier-protein] reductase (NADPH) (n-C14:1) |
| R_EAR160x | enoyl-[acyl-carrier-protein] reductase (NADH) (n-C16:0) |
| R_EAR160y | enoyl-[acyl-carrier-protein] reductase (NADPH) (n-C16:0) |
| R_EAR161x | enoyl-[acyl-carrier-protein] reductase (NADH) (n-C16:1) |
| R_EAR161y | enoyl-[acyl-carrier-protein] reductase (NADPH) (n-C16:1) |
| R_EAR180x | enoyl-[acyl-carrier-protein] reductase (NADH) (n-C18:0) |
| R_EAR180y | enoyl-[acyl-carrier-protein] reductase (NADPH) (n-C18:0) |
| R_EAR181x | enoyl-[acyl-carrier-protein] reductase (NADH) (n-C18:1) |
| R_EAR181y | enoyl-[acyl-carrier-protein] reductase (NADPH) (n-C18:1) |
| R_EAR40x | enoyl-[acyl-carrier-protein] reductase (NADH) (n-C4:0) |
| R_EAR40y | enoyl-[acyl-carrier-protein] reductase (NADPH) (n-C4:0) |
| R_EAR60x | enoyl-[acyl-carrier-protein] reductase (NADH) (n-C6:0) |
| R_EAR60y | enoyl-[acyl-carrier-protein] reductase (NADPH) (n-C6:0) |
| R_EAR80x | enoyl-[acyl-carrier-protein] reductase (NADH) (n-C8:0) |
| R_EAR80y | enoyl-[acyl-carrier-protein] reductase (NADPH) (n-C8:0) |
| R_ECA4COLIPAabctex | enterobacterial common antigen (x4) core oligosaccharide lipid A transport via ABC system (periplasm to extracellular) |
| R_ECA4OALpp | enterobacterial common antigen (x4) O-antigen ligase (periplasm) |
| R_ECAP1pp | enterobacterial common antigen polymerase (periplasm) |
| R_ECAP2pp | enterobacterial common antigen polymerase (periplasm) |
| R_ECAP3pp | enterobacterial common antigen polymerase (periplasm) |
| R_ECAtpp | enterobacterial common antigen transferase (flippase, cytoplasm to periplasm) |
| R_ECOAH1 | 3-hydroxyacyl-CoA dehydratase (3-hydroxybutanoyl-CoA) |
| R_ECOAH2 | 3-hydroxyacyl-CoA dehydratase (3-hydroxyhexanoyl-CoA) |
| R_ECOAH3 | 3-hydroxyacyl-CoA dehydratase (3-hydroxyoctanoyl-CoA) |
| R_ECOAH4 | 3-hydroxyacyl-CoA dehydratase (3-hydroxydecanoyl-CoA) |
| R_ECOAH5 | 3-hydroxyacyl-CoA dehydratase (3-hydroxydodecanoyl-CoA) |
| R_ECOAH6 | 3-hydroxyacyl-CoA dehydratase (3-hydroxytetradecanoyl-CoA) |
| R_ECOAH7 | 3-hydroxyacyl-CoA dehydratase (3-hydroxyhexadecanoyl-CoA) |
| R_ECOAH8 | 3-hydroxyacyl-CoA dehydratase (3-hydroxyoctadecanoyl-CoA) |
| R_EDA | 2-dehydro-3-deoxy-phosphogluconate aldolase |
| R_EDD | 6-phosphogluconate dehydratase |
| R_EDTXS1 | Endotoxin Synthesis (lauroyl transferase) |
| R_EDTXS2 | Endotoxin Synthesis (myristoyl transferase) |
| R_EDTXS3 | Endotoxin Synthesis (palmitoleoyl ACP) |
| R_EDTXS4 | Endotoxin Synthesis (myristoyl transferase) |
| R_EGMEACPR | Enoylglutaryl-[ACP] methyl ester reductase |
| R_ENLIPAabctex | phosphoethanolamine lipid A transport via ABC system (periplasm to extracellular) |
| R_ENO | enolase |
| R_ENTCS | enterochelin synthase |
| R_ENTERES | Enterochelin Esterase |
| R_ENTERES2 | Enterochelin Esterase (Fe containing) |
| R_EPMEACPR | Enoylpimeloyl-[ACP] methyl ester reductase |
| R_ETHAAL | Ethanolamine ammonia-lyase |
| R_ETHAt2pp | ethanolamine transport in via proton symport |
| R_ETHAtex | ethanolamine transport via diffusion (extracellular) |
| R_ETHSO3abcpp | ethanesulfonate transport via ABC system (periplasm) |
| R_ETHSO3tex | ethanesulfonate transport via diffusion (extracellular to periplasm) |
| R_ETOHtex | ethanol transport via diffusion (extracellular to periplasm) |
| R_ETOHtrpp | ethanol reversible transport via diffusion (periplasm) |
| R_F6PA | fructose 6-phosphate aldolase |
| R_F6PP | D-fructose 6-phosphate phosphatase |
| R_F6Pt6_2pp | Fructose-6-phosphate transport via phosphate antiport (periplasm) |
| R_F6Ptex | fructose 6-phosphate transport via diffusion (extracellular to periplasm) |
| R_FA100ACPHi | fatty-acyl-ACP hydrolase |
| R_FA120ACPHi | fatty-acyl-ACP hydrolase |
| R_FA140ACPHi | fatty-acyl-ACP hydrolase |
| R_FA141ACPHi | fatty-acyl-ACP hydrolase |
| R_FA160ACPHi | fatty-acyl-ACP hydrolase |
| R_FA161ACPHi | fatty-acyl-ACP hydrolase |
| R_FA80ACPHi | fatty-acyl-ACP hydrolase |
| R_FACOAE100 | fatty-acid-CoA thioesterase (decanoate) |
| R_FACOAE120 | fatty-acid-CoA thioesterase (dodecanoate) |
| R_FACOAE140 | fatty-acid-CoA thioesterase (tetradecanoate) |
| R_FACOAE141 | fatty-acid-CoA thioesterase (tetradecenoate) |
| R_FACOAE160 | fatty-acid-CoA thioesterase (hexadecanoate) |
| R_FACOAE161 | fatty-acid-CoA thioesterase (hexadecenoate) |
| R_FACOAE180 | fatty-acid-CoA thioesterase (octadecanoate) |
| R_FACOAE181 | fatty-acid-CoA thioesterase (octadecenoate) |
| R_FACOAE60 | fatty-acid-CoA thioesterase (hexanoate) |
| R_FACOAE80 | fatty-acid-CoA thioesterase (octanoate) |
| R_FACOAL100t2pp | fatty-acid-CoA ligase (decanoate transport via vectoral Co-A coupling) |
| R_FACOAL120t2pp | fatty-acid-CoA ligase (dodecanoate transport via vectoral Co-A coupling) |
| R_FACOAL140t2pp | fatty-acid-CoA ligase (tetradecanoate transport via vectoral Co-A coupling) |
| R_FACOAL141t2pp | fatty-acid-CoA ligase (tetradecenoate transport via vectoral Co-A coupling) |
| R_FACOAL160t2pp | fatty-acid-CoA ligase (hexadecanoate transport via vectoral Co-A coupling) |
| R_FACOAL161t2pp | fatty-acid-CoA ligase (hexadecenoate transport via vectoral Co-A coupling) |
| R_FACOAL180t2pp | fatty-acid-CoA ligase (octadecanoate transport via vectoral Co-A coupling) |
| R_FACOAL181t2pp | fatty-acid-CoA ligase (octadecenoate transport via vectoral Co-A coupling) |
| R_FACOAL60t2pp | fatty-acid-CoA ligase (hexanoate transport via vectoral Co-A coupling) |
| R_FACOAL80t2pp | fatty-acid-CoA ligase (octanoate transport via vectoral Co-A coupling) |
| R_FADRx | FAD reductase |
| R_FADRx2 | FAD reductase |
| R_FALDH2 | formaldehyde dehydrogenase |
| R_FALDtex | formaldehyde transport via diffusion (extracellular to periplasm) |
| R_FALDtpp | formaldehyde transport via diffusion (periplasm) |
| R_FALGTHLs | formaldehyde glutathione ligase (spontaneous) |
| R_FBA | fructose-bisphosphate aldolase |
| R_FBA3 | Sedoheptulose 1,7-bisphosphate D-glyceraldehyde-3-phosphate-lyase |
| R_FBP | fructose-bisphosphatase |
| R_FCI | L-fucose isomerase |
| R_FCLK | L-fuculokinase |
| R_FCLPA | L-fuculose 1-phosphate aldolase |
| R_FCLT | Ferrochelatase |
| R_FDH4pp | formate dehydrogenase (quinone-8) (periplasm) |
| R_FDH5pp | Formate Dehydrogenase (menaquinone-8) (periplasm) |
| R_FDMO | FMNH2-dependent monooxygenase |
| R_FDMO2 | FMNH2-dependent monooxygenase (methanesulfonate) |
| R_FDMO3 | FMNH2-dependent monooxygenase (ethanesulfonate) |
| R_FDMO4 | FMNH2-dependent monooxygenase (butanesulfonate) |
| R_FDMO6 | FMNH2-dependent monooxygenase (sulfoacetate) |
| R_FE2abcpp | iron (II) transport via ABC system (periplasm) |
| R_FE2t2pp | iron (II) transport in via proton symport (periplasm) |
| R_FE2t3pp | iron (Fe+2) transport out via proton antiport (periplasm) |
| R_FE2tex | iron (II) transport via diffusion (extracellular to periplasm) |
| R_FE2tpp | iron (+2) transport in via permease (no H+) |
| R_FE3DCITabcpp | iron transport from ferric-dicitrate via ABC system (periplasm) |
| R_FE3DCITtonex | ferric-dicitrate transport via ton system (extracellular) |
| R_FE3DHBZR | release of Fe(III) from ferric 2,3-dihydroxybenzoylserine |
| R_FE3DHBZSabcpp | ferric 2,3-dihydroxybenzoylserine transport via ABC system (periplasm) |
| R_FE3DHBZStonex | ferric 2,3-dihydroxybenzoylserine transport via ton system (extracellular) |
| R_FE3HOXR1 | Fe(III)hydroxamate reductase |
| R_FE3HOXR2 | Fe(III)hydroxamate reductase |
| R_FE3HOXR3 | Fe(III)hydroxamate reductase |
| R_FE3HOXUtex | Fe(III)hydroxamate unloaded secretion |
| R_FE3HOXUtpp | Fe(III)hydroxamate unloaded secretion (extracellular) |
| R_FE3HOXabcpp | ferric-dicitrate transport via ABC system (periplasm) |
| R_FE3HOXexs | Fe(III) hydroxamate Fe-loading reaction (spontaneaous) |
| R_FE3HOXtonex | Fe(III)hydroxamine transport via ton system (extracellular) |
| R_FE3Ri | Fe(III) reduction |
| R_FE3abcpp | iron (III) transport via ABC system (periplasm to cytoplasm) |
| R_FE3tex | iron (III) transport via diffusion (extracellular to periplasm) |
| R_FECRMR1 | Ferrichrome reductase |
| R_FECRMR2 | Ferrichrome reductase |
| R_FECRMR3 | Ferrichrome reductase |
| R_FECRMUtex | ferrichrome (minus Fe) secretion (to extracellular) |
| R_FECRMUtpp | ferrichrome (minus Fe) secretion (to periplasm) |
| R_FECRMabcpp | ferrichrome transport via ABC system (periplasm) |
| R_FECRMexs | ferrichrome Fe(III)-loading reaction (spontaneous) |
| R_FECRMtonex | ferrichrome transport via ton system (extracellular) |
| R_FEENTERR1 | Fe-enterobactin reduction (Fe(III)-unloading) |
| R_FEENTERR2 | Fe-enterobactin reduction (Fe(III)-unloading) |
| R_FEENTERR3 | Fe-enterobactin reduction (Fe(III)-unloading) |
| R_FEENTERabcpp | Fe-enterobactin transport via ABC system (periplasm) |
| R_FEENTERexs | enterobactin Fe(III) binding (spontaneous) |
| R_FEENTERtex | enterochelin transport (secretion periplasm) |
| R_FEENTERtonex | Fe-enterobactin transport via ton system (extracellular) |
| R_FEENTERtpp | enterochelin transport (secretion) |
| R_FEOXAMR1 | ferroxamine reductase |
| R_FEOXAMR2 | ferroxamine reductase |
| R_FEOXAMR3 | ferroxamine reductase |
| R_FEOXAMUtex | ferroxamine (minus Fe3) secretion (to extracellular) |
| R_FEOXAMUtpp | ferroxamine (minus Fe3) secretion (to periplasm) |
| R_FEOXAMabcpp | ferroxamine transport via ABC system (periplasm) |
| R_FEOXAMexs | ferroxamine Fe3-loading reaction (spontaneous) |
| R_FEOXAMtonex | ferroxamine transport via ton system (extracellular) |
| R_FEROpp | ferroxidase |
| R_FESD1s | iron-sulfur cluster damage (peroxide, spontaneous) |
| R_FESD2s | iron-sulfur cluster damage (nitrous oxide, spontaneous) |
| R_FESR | iron-sulfur cluster repair |
| R_FFSD | beta-fructofuranosidase |
| R_FHL | Formate-hydrogen lyase |
| R_FLDR2 | flavodoxin reductase (NADPH) |
| R_FLVR | flavin reductase |
| R_FLVRx | flavin reductase (NAD) |
| R_FMETTRS | Methionyl-tRNA formyltransferase |
| R_FMNAT | FMN adenylyltransferase |
| R_FMNRx | FMN reductase |
| R_FMNRx2 | FMN reductase |
| R_FOMETRi | Aminomethyltransferase |
| R_FORCT | Formyl-CoA Transferase |
| R_FORt2pp | formate transport via proton symport (uptake only, periplasm) |
| R_FORtex | formate transport via diffusion (extracellular to periplasm) |
| R_FORtppi | formate transport via diffusion (cytoplasm to periplasm) |
| R_FRD2 | fumarate reductase |
| R_FRD3 | fumarate reductase |
| R_FRUK | fructose-1-phosphate kinase |
| R_FRULYSDG | Fructoselysine phosphate deglycase |
| R_FRULYSE | fructoselysine 3-epimerase |
| R_FRULYSK | Fructoselysine Kinase |
| R_FRULYSt2pp | Fructoselysine transport via proton symport (periplasm) |
| R_FRULYStex | fructoselysine transporter via diffusion (extracellular) |
| R_FRUURt2rpp | D-fructuronate transport via proton symport, reversible (periplasm) |
| R_FRUURtex | D-fructuronate transport via diffusion (extracellular) |
| R_FRUpts2pp | Fructose transport via PEP:Pyr PTS (f6p generating) (periplasm) |
| R_FRUptspp | D-fructose transport via PEP:Pyr PTS (periplasm) |
| R_FRUtex | D-fructose transport via diffusion (extracellular to periplasm) |
| R_FTHFD | formyltetrahydrofolate deformylase |
| R_FTHFLi | formate-tetrahydrofolate ligase |
| R_FUCtex | L-fucose transport via diffusion (extracellular to periplasm) |
| R_FUCtpp | L-fucose transport via proton symport (periplasm) |
| R_FUM | fumarase |
| R_FUMt2_2pp | Fumarate transport via proton symport (2 H) (periplasm) |
| R_FUMt2_3pp | Fumarate transport via proton symport (3 H) (periplasm) |
| R_FUMtex | Fumarate transport via diffusion (extracellular to periplasm) |
| R_FUSAtex | Fusidic acid transport via diffusion (extracellular to periplasm) |
| R_FUSAtpp | Fusidic acid transport via TolC system |
| R_G1PACT | glucosamine-1-phosphate N-acetyltransferase |
| R_G1PPpp | Glucose-1-phosphatase |
| R_G1PTT | glucose-1-phosphate thymidylyltransferase |
| R_G1Ptex | D-glucose 1-phosphate transport via diffusion |
| R_G1SAT | glutamate-1-semialdehyde aminotransferase |
| R_G2PP | glycerol-2-phosphate phosphatase |
| R_G2PPpp | glycerol 2-phosphate phosphatase (periplasmic) |
| R_G3PAT120 | glycerol-3-phosphate acyltransferase (C12:0) |
| R_G3PAT140 | glycerol-3-phosphate acyltransferase (C14:0) |
| R_G3PAT141 | glycerol-3-phosphate acyltransferase (C14:1) |
| R_G3PAT160 | glycerol-3-phosphate acyltransferase (C16:0) |
| R_G3PAT161 | glycerol-3-phosphate acyltransferase (C16:1) |
| R_G3PAT180 | glycerol-3-phosphate acyltransferase (C18:0) |
| R_G3PAT181 | glycerol-3-phosphate acyltransferase (C18:1) |
| R_G3PCabcpp | sn-glycerol-3-phosphocholine transport via ABC system (periplasm) |
| R_G3PCtex | glycero-3-phosphocholine transport via diffusion (extracellular to periplasm) |
| R_G3PD2 | glycerol-3-phosphate dehydrogenase (NADP) |
| R_G3PD5 | glycerol-3-phosphate dehydrogenase (ubiquinone-8) |
| R_G3PD6 | glycerol-3-phosphate dehydrogenase (menaquinone-8) |
| R_G3PD7 | glycerol-3-phosphate dehydrogenase (demethylmenaquinone-8) |
| R_G3PEabcpp | sn-glycerol-3-phosphoethanolamine transport via ABC system (periplasm) |
| R_G3PEtex | glycero-3-phosphoethanolamine transport via diffusion (extracellular to periplasm) |
| R_G3PGabcpp | sn-glycerol-3-phosphoglycerol transport via ABC system (periplasm) |
| R_G3PGtex | glycerophoglycerol transport via diffusion (extracellular to periplasm) |
| R_G3PIabcpp | sn-glycerol-3-phosphoethanolamine transport via ABC system (periplasm) |
| R_G3PItex | glycero-3-phospho-1-inositol transport via diffusion (extracellular to periplasm) |
| R_G3PSabcpp | sn-glycerol-3-phosphoserine transport via ABC system (periplasm) |
| R_G3PStex | glycerophosphserine transport via diffusion (extracellular to periplasm) |
| R_G3PT | glycerol-3-phosphatase |
| R_G5SADs | L-glutamate 5-semialdehyde dehydratase (spontaneous) |
| R_G5SD | glutamate-5-semialdehyde dehydrogenase |
| R_G6PDA | glucosamine-6-phosphate deaminase |
| R_G6PDH2r | glucose 6-phosphate dehydrogenase |
| R_G6PP | glucose-6-phosphate phosphatase |
| R_G6Pt6_2pp | Glucose-6-phosphate transport via phosphate antiport (periplasm) |
| R_G6Ptex | glucose 6-phosphate transport via diffusion (extracellular to periplasm) |
| R_GAL1PPpp | D-galactose 1-phosphatase |
| R_GAL1Ptex | D-galactose 1-phosphate transport via diffusion (extracellular to periplasm) |
| R_GALBDtex | beta D-galactose transport via diffusion (extracellular to periplasm) |
| R_GALCTD | galactarate dehydratase |
| R_GALCTLO | L-galactonate oxidoreductase |
| R_GALCTND | galactonate dehydratase |
| R_GALCTNLt2pp | L-galactonate transport via proton symport (periplasm) |
| R_GALCTNLtex | L-galactonate transport via diffusion (extracellular to periplasm) |
| R_GALCTNt2pp | D-galactonate transport via proton symport (periplasm) |
| R_GALCTNtex | D-galactonate transport via diffusion (extracellular to periplasm) |
| R_GALCTt2rpp | D-galactarte transport via proton symport, reversible (periplasm) |
| R_GALCTtex | D-galactarte transport via diffusion (extracellular to periplasm) |
| R_GALKr | galactokinase |
| R_GALM2pp | aldose-1-epimerase |
| R_GALS3 | a-galactosidase (melibiose) |
| R_GALT1 | galactosyltransferase I (LPS core synthesis) |
| R_GALTptspp | Galactitol transport via PEP:Pyr PTS (periplasm) |
| R_GALTtex | Galactitol transport via diffusion (extracellular to periplasm) |
| R_GALURt2rpp | D-galacturonate transport via proton symport, reversible (periplasm) |
| R_GALURtex | D-galacturonate transport via diffusion (extracellular to periplasm) |
| R_GALUi | UTP-glucose-1-phosphate uridylyltransferase (irreversible) |
| R_GALabcpp | D-galactose transport via ABC system (periplasm) |
| R_GALt2pp | D-galactose transport in via proton symport (periplasm) |
| R_GALtex | D-galactose transport via diffusion (extracellular to periplasm) |
| R_GAM6Pt6_2pp | D-Glucosamine 6-phosphate transport via phosphate antiport (periplasm) |
| R_GAMAN6Ptex | D-glucosamine 6-phosphate transport via diffusion (extracellular to periplasm) |
| R_GAMptspp | D-glucosamine transport via PEP:Pyr PTS (periplasm) |
| R_GAMtex | D-glucosamine transport via diffusion (extracellular to periplasm) |
| R_GAPD | glyceraldehyde-3-phosphate dehydrogenase |
| R_GARFT | phosphoribosylglycinamide formyltransferase |
| R_GART | GAR transformylase-T |
| R_GBBTNtex | gamma-butyrobetaine transport via diffusion (extracellular to periplasm) |
| R_GCALDD | Glycolaldehyde dehydrogenase |
| R_GDMANE | GDP-4-dehydro-6-deoxy-D-mannose epimerase |
| R_GDPDPK | GDP diphosphokinase |
| R_GDPMNH | GDP-mannose mannosyl hydrolase |
| R_GDPMNP | GDP-mannose phyrophosphatase |
| R_GDPTPDP | guanosine 3'-diphosphate 5'-triphosphate 3'-diphosphatase |
| R_GDPtex | GDP transport via diffusion (extracellular to periplasm) |
| R_GF6PTA | glutamine-fructose-6-phosphate transaminase |
| R_GGGABADr | gamma-glutamyl-gamma aminobutyric acid dehydrogenase |
| R_GGGABAH | gamma-glutamyl-gamma-aminobutyric acid hydrolase |
| R_GGPTRCO | gamma glutamyl putrescine oxidase |
| R_GGPTRCS | gamma glutamyl putrescine synthase |
| R_GHBDHx | gamma-hydroxybutyrate dehydrogenase (NADH) |
| R_GHMT2r | glycine hydroxymethyltransferase, reversible |
| R_GK1 | guanylate kinase (GMP:ATP) |
| R_GLBRAN2 | 1,4-alpha-glucan branching enzyme (glycogen -> bglycogen) |
| R_GLCATr | D-glucose O-acetyltransferase |
| R_GLCDpp | Glucose dehydrogenase (ubiquinone-8 as acceptor) (periplasm) |
| R_GLCNt2rpp | D-gluconate transport via proton symport, reversible (periplasm) |
| R_GLCNtex | D-gluconate transport via diffusion (extracellular to periplasm) |
| R_GLCP | glycogen phosphorylase |
| R_GLCP2 | glycogen phosphorylase |
| R_GLCRAL | 5-dehydro-4-deoxyglucarate aldolase |
| R_GLCRD | glucarate dehydratase |
| R_GLCRt2rpp | D-glucarate transport via proton symport, reversible (periplasm) |
| R_GLCRtex | D-glucarate transport via diffusion (extracellular to periplasm) |
| R_GLCS1 | glycogen synthase (ADPGlc) |
| R_GLCTR1 | glucosyltransferase I (LPS core synthesis) |
| R_GLCTR2 | glucosyltransferase II (LPS core synthesis) |
| R_GLCTR3 | glucosyltransferase III (LPS core synthesis) |
| R_GLCUR1Ptex | D-glucuronate 1-phosphate transport via diffusion (extracellular to periplasm) |
| R_GLCURt2rpp | D-glucuronate transport via proton symport, reversible (periplasm) |
| R_GLCURtex | D-glucuronat transport via diffusion (extracellular to periplasm) |
| R_GLCabcpp | D-glucose transport via ABC system (periplasm) |
| R_GLCptspp | D-glucose transport via PEP:Pyr PTS (periplasm) |
| R_GLCt2pp | D-glucose transport in via proton symport (periplasm) |
| R_GLCtex | glucose transport via diffusion (extracellular to periplasm) |
| R_GLCtexi | D-glucose transport via diffusion (extracellular to periplasm) irreversible |
| R_GLDBRAN2 | glycogen debranching enzyme (bglycogen -> glycogen) |
| R_GLGC | glucose-1-phosphate adenylyltransferase |
| R_GLNS | glutamine synthetase |
| R_GLNTRS | Glutaminyl-tRNA synthetase |
| R_GLNabcpp | L-glutamine transport via ABC system (periplasm) |
| R_GLNtex | L-glutamine transport via diffusion (extracellular to periplasm) |
| R_GLTPD | Galactitol-1-phosphate dehydrogenase |
| R_GLU5K | glutamate 5-kinase |
| R_GLUABUTt7pp | 4-aminobutyrate/glutamate antiport (periplasm) |
| R_GLUCYS | gamma-glutamylcysteine synthetase |
| R_GLUDC | Glutamate Decarboxylase |
| R_GLUDy | glutamate dehydrogenase (NADP) |
| R_GLUN | glutaminase |
| R_GLUNpp | glutaminase |
| R_GLUPRT | glutamine phosphoribosyldiphosphate amidotransferase |
| R_GLUR | glutamate racemase |
| R_GLUSy | glutamate synthase (NADPH) |
| R_GLUTRR | glutamyl-tRNA reductase |
| R_GLUTRS | Glutamyl-tRNA synthetase |
| R_GLUabcpp | L-glutamate transport via ABC system (periplasm) |
| R_GLUt2rpp | L-glutamate transport via proton symport, reversible (periplasm) |
| R_GLUt4pp | Na+/glutamate symport (periplasm) |
| R_GLUtex | L-glutamate transport via diffusion (extracellular to periplasm) |
| R_GLXCL | glyoxalate carboligase |
| R_GLYALDtex | Glyceraldehyde transport via diffusion (extracellular to periplasm) |
| R_GLYALDtpp | Glyceraldehyde facilitated diffusion (periplasm) |
| R_GLYAT | glycine C-acetyltransferase |
| R_GLYBabcpp | Glycine betaine transport via ABC system (periplasm) |
| R_GLYBt2pp | Glycine betaine transport via proton symport (periplasm) |
| R_GLYBtex | Glycine betaine transport via diffusion (extracellular to periplasm) |
| R_GLYC2Pabcpp | sn-Glycerol 2-phosphate transport via ABC system (periplasm) |
| R_GLYC2Ptex | Glycerol-2-phosphate transport via diffusion (extracellular to periplasm) |
| R_GLYC3Pabcpp | sn-Glycerol 3-phosphate transport via ABC system (periplasm) |
| R_GLYC3Pt6pp | Glycerol-3-phosphate : phosphate antiporter (periplasm) |
| R_GLYC3Ptex | Glycerol-3-phosphate transport via diffusion (extracellular to periplasm) |
| R_GLYCAt2rpp | D-glycerate transport via proton symport, reversible (periplasm) |
| R_GLYCAtex | D-glycerate transport via diffusion (extracellular to periplasm) |
| R_GLYCDx | Glycerol dehydrogenase |
| R_GLYCK | glycerate kinase |
| R_GLYCK2 | glycerate kinase |
| R_GLYCL | Glycine Cleavage System |
| R_GLYCLTDx | Glycolate dehydrogenase (NAD) |
| R_GLYCLTDy | Glycolate dehydrogenase (NADP) |
| R_GLYCLTt2rpp | glycolate transport via proton symport, reversible (periplasm) |
| R_GLYCLTt4pp | glycolate transport via sodium symport (periplasm) |
| R_GLYCLTtex | glycolate transport via diffusion (extracellular to periplasm) |
| R_GLYCTO2 | Glycolate oxidase |
| R_GLYCTO3 | Glycolate oxidase |
| R_GLYCTO4 | Glycolate oxidase |
| R_GLYCtex | glycerol transport via diffusion (extracellular to periplasm) |
| R_GLYCtpp | glycerol transport via channel (periplasm) |
| R_GLYK | glycerol kinase |
| R_GLYOX | hydroxyacylglutathione hydrolase |
| R_GLYOX3 | glyoxalase III |
| R_GLYTRS | Glycyl-tRNA synthetase |
| R_GLYt2pp | glycine transport in via proton symport (periplasm) |
| R_GLYt2rpp | glycine reversible transport via proton symport (periplasm) |
| R_GLYt4pp | glycine transport in via sodium symport (periplasm) |
| R_GLYtex | Glycine transport via diffusion (extracellular to periplasm) |
| R_GMAND | GDP-D-mannose dehydratase |
| R_GMHEPAT | D-glycero-D-manno-hepose 1-phosphate adenyltransferase |
| R_GMHEPK | D-glycero-D-manno-heptose 7-phosphate kinase |
| R_GMHEPPA | D-glycero-D-manno-heptose 1,7-bisphosphate phosphatase |
| R_GMPR | GMP reductase |
| R_GMPS2 | GMP synthase |
| R_GMPtex | GMP transport via diffusion (extracellular to periplasm) |
| R_GND | phosphogluconate dehydrogenase |
| R_GNK | gluconokinase |
| R_GOFUCR | GDP-4-oxo-L-fucose reductase |
| R_GP4GH | Gp4G hydrolase |
| R_GPDDA1 | Glycerophosphodiester phosphodiesterase (Glycerophosphocholine) |
| R_GPDDA1pp | Glycerophosphodiester phosphodiesterase (Glycerophosphocholine) |
| R_GPDDA2 | Glycerophosphodiester phosphodiesterase (Glycerophosphoethanolamine) |
| R_GPDDA2pp | Glycerophosphodiester phosphodiesterase (Glycerophosphoethanolamine) |
| R_GPDDA3 | Glycerophosphodiester phosphodiesterase (Glycerophosphoserine) |
| R_GPDDA3pp | Glycerophosphodiester phosphodiesterase (Glycerophosphoserine) |
| R_GPDDA4 | Glycerophosphodiester phosphodiesterase (Glycerophosphoglycerol) |
| R_GPDDA4pp | Glycerophosphodiester phosphodiesterase (Glycerophosphoglycerol) |
| R_GPDDA5 | Glycerophosphodiester phosphodiesterase (Glycerophosphoinositol) |
| R_GPDDA5pp | Glycerophosphodiester phosphodiesterase (Glycerophosphoinositol) |
| R_GRTT | geranyltranstransferase |
| R_GRXR | glutaredoxin reductase |
| R_GSNK | guanosine kinase |
| R_GSNt2pp | guanosine transport in via proton symport (periplasm) |
| R_GSNtex | guanosine transport via diffusion (extracellular to periplasm) |
| R_GSPMDA | Glutathionylspermidine amidase |
| R_GSPMDS | Glutathionylspermidine synthetase |
| R_GTHOXtex | glutathione (ox) transport via diffusion (extracellular to periplasm) |
| R_GTHOr | glutathione oxidoreductase |
| R_GTHPi | glutathione peridoxase |
| R_GTHRDHpp | glutathione hydralase (periplasmic) |
| R_GTHRDabc2pp | glutathione export via ABC system (cytoplasm to periplasm) |
| R_GTHRDabcpp | Reduced glutathione via ABC system (periplasm) |
| R_GTHRDtex | glutathione transport via diffusion (extracellular to periplasm) |
| R_GTHS | glutathione synthetase |
| R_GTPCI | GTP cyclohydrolase I |
| R_GTPCII2 | GTP cyclohydrolase II (25drapp) |
| R_GTPDPDP | guanosine-5'-triphosphate,3'-diphosphate diphosphatase |
| R_GTPDPK | GTP diphosphokinase |
| R_GTPHs | GTP amine hydrolysis (spontaneous) |
| R_GTPtex | GTP transport via diffusion (extracellular to periplasm) |
| R_GUACYC | guanylate cyclase |
| R_GUAD | guanine deaminase |
| R_GUAPRT | guanine phosphoribosyltransferase |
| R_GUAt2pp | guanine transport in via proton symport (periplasm) |
| R_GUAtex | Guanine transport via diffusion (extracellular to periplasm) |
| R_GUAtpp | Guanine transport via diffusion (periplasm) |
| R_GUI1 | glucuronate isomerase (D-glucuronate) |
| R_GUI2 | glucuronate isomerase (D-galacturonate) |
| R_GUR1PPpp | Glucuronate 1-phosphate phosphatase (periplasm) |
| R_H2O2tex | hydrogen peroxide transport via diffusion (external) |
| R_H2Otex | H2O transport via diffusion (extracellular to periplasm) |
| R_H2Otpp | H2O transport via diffusion (periplasm) |
| R_H2SO | Hydrogen sulfide oxidation |
| R_H2St1pp | h2s transport (periplasm) |
| R_H2Stex | h2s transport via diffusion (extracellular to periplasm) |
| R_H2tex | hydrogen transport via diffusion (extracellular to periplasm) |
| R_H2tpp | hydrogen transport diffusion (periplasm) |
| R_HACD1 | 3-hydroxyacyl-CoA dehydrogenase (acetoacetyl-CoA) |
| R_HACD2 | 3-hydroxyacyl-CoA dehydrogenase (3-oxohexanoyl-CoA) |
| R_HACD3 | 3-hydroxyacyl-CoA dehydrogenase (3-oxooctanoyl-CoA) |
| R_HACD4 | 3-hydroxyacyl-CoA dehydrogenase (3-oxodecanoyl-CoA) |
| R_HACD5 | 3-hydroxyacyl-CoA dehydrogenase (3-oxododecanoyl-CoA) |
| R_HACD6 | 3-hydroxyacyl-CoA dehydrogenase (3-oxotetradecanoyl-CoA) |
| R_HACD7 | 3-hydroxyacyl-CoA dehydrogenase (3-oxohexadecanoyl-CoA) |
| R_HACD8 | 3-hydroxyacyl-CoA dehydrogenase (3-oxooctadecanoyl-CoA), peroxisomal |
| R_HADPCOADH3 | 3-hydroxyadipyl-CoA dehydrogenase (NAD+) |
| R_HBZOPT | Hydroxybenzoate octaprenyltransferase |
| R_HCINNMt2rpp | 3-hydroxycinnamic acid transport via proton symport, reversible (periplasm) |
| R_HCINNMtex | 3-hydroxycinnamic acid transport via diffusion (extracellular to periplasm) |
| R_HCO3E | HCO3 equilibration reaction |
| R_HCYSMT | homocysteine S-methyltransferase |
| R_HCYSMT2 | Homocysteine Methyltransferase |
| R_HDCAtexi | Hexadecanoate transport via facilitated irreversible diffusion (extracellular to periplasm) |
| R_HDCEAtexi | Hexadecenoate transport via facilitated irreversible diffusion (extracellular to periplasm) |
| R_HEMEOS | Heme O synthase |
| R_HEPK1 | LPS heptose kinase I (LPS core synthesis) |
| R_HEPK2 | LPS heptose kinase II (LPS core synthesis) |
| R_HEPT1 | heptosyltransferase I (LPS core synthesis) |
| R_HEPT2 | heptosyltransferase II (LPS core synthesis) |
| R_HEPT3 | heptosyltransferase III (LPS core synthesis) |
| R_HEPT4 | heptosyltransferase IV (LPS core synthesis) |
| R_HETZK | hydroxyethylthiazole kinase |
| R_HEX1 | hexokinase (D-glucose:ATP) |
| R_HEX4 | hexokinase (D-mannose:ATP) |
| R_HEX7 | hexokinase (D-fructose:ATP) |
| R_HEXt2rpp | hexanoate transport via proton symport, reversible (periplasm) |
| R_HG2abcpp | Mercury (Hg+2) ABC transporter (periplasm) |
| R_HG2t3pp | mercury (Hg+2) transport out via proton antiport (periplasm) |
| R_HG2tex | mercury (Hg+2) transport via diffusion (extracellular to periplasm) |
| R_HISTD | histidinol dehydrogenase |
| R_HISTP | histidinol-phosphatase |
| R_HISTRS | Histidyl-tRNA synthetase |
| R_HISabcpp | L-histidine transport via ABC system (periplasm) |
| R_HISt2rpp | L-histidine reversible transport via proton symport (periplasm) |
| R_HIStex | L-histidine transport via diffusion (extracellular to periplasm) |
| R_HKNDDH | 2-hydroxy-6-ketonona-2,4-dienedioic acid hydrolase |
| R_HKNTDH | 2-hydroxy-6-ketononotrienedioate hydrolase |
| R_HMBS | hydroxymethylbilane synthase |
| R_HMPK1 | hydroxymethylpyrimidine kinase (ATP) |
| R_HOMt2pp | L-homoserineserine efflux via proton symport |
| R_HOMtex | L-homoserine transport via diffusion (extracellular to periplasm) |
| R_HOPNTAL | 4-hydroxy-2-oxopentanoate aldolase |
| R_HPPK2 | 6-hydroxymethyl-dihydropterin pyrophosphokinase |
| R_HPPPNDO | 2,3-dihydroxypheylpropionate 1,2-dioxygenase |
| R_HPPPNt2rpp | 3-(3-hydroxyphenyl)propionate transport via proton symport, reversible (periplasm) |
| R_HPPPNtex | 3-(3-hydroxyphenyl)propionate transport via diffusion (extracellular to periplasm) |
| R_HPYRI | hydroxypyruvate isomerase |
| R_HPYRRx | Hydroxypyruvate reductase (NADH) |
| R_HPYRRy | Hydroxypyruvate reductase (NADPH) |
| R_HSDy | homoserine dehydrogenase (NADPH) |
| R_HSK | homoserine kinase |
| R_HSST | homoserine O-succinyltransferase |
| R_HSTPT | histidinol-phosphate transaminase |
| R_HXAND | hypoxanthine dehydrogenase |
| R_HXAtex | Hexanoate transport via diffusion (extracellular to periplasm) |
| R_HXCT | Acetyl-CoA:hexanoate-CoA transferase |
| R_HXPRT | hypoxanthine phosphoribosyltransferase (Hypoxanthine) |
| R_HYD1pp | hydrogenase (ubiquinone-8: 2 protons) (periplasm) |
| R_HYD2pp | Hydrogenase (menaquinone8: 2 protons) (periplasm) |
| R_HYD3pp | Hydrogenase (Demethylmenaquinone-8: 2 protons) (periplasm) |
| R_HYPOE | hypothetical enyme |
| R_HYXNtex | Hypoxanthine transport via diffusion (extracellular to periplasm) |
| R_HYXNtpp | Hypoxanthine transport (periplasm) |
| R_Htex | proton transport via diffusion (extracellular to periplasm) |
| R_I2FE2SR | ISC [2Fe-2S] regeneration |
| R_I2FE2SS | ISC [2Fe-2S] Synthesis |
| R_I2FE2SS2 | ISC [2Fe-2S] Synthesis II |
| R_I2FE2ST | ISC [2Fe-2S] Transfer |
| R_I4FE4SR | ISC [4Fe-4S] Reduction |
| R_I4FE4ST | ISC [4Fe-4S] Transfer |
| R_ICDHyr | isocitrate dehydrogenase (NADP) |
| R_ICHORS | isochorismate synthase |
| R_ICHORSi | Isochorismate Synthase |
| R_ICHORT | isochorismatase |
| R_ICL | Isocitrate lyase |
| R_ICYSDS | ISC Cysteine desulfuration |
| R_IDOND | L-idonate 5-dehydrogenase |
| R_IDOND2 | L-indonate 5-dehydrogenase (NADP) |
| R_IDONt2rpp | L-idonate transport via proton symport, reversible (periplasm) |
| R_IDONtex | L-idonate transport via diffusion (extracellular to periplasm) |
| R_IG3PS | Imidazole-glycerol-3-phosphate synthase |
| R_IGPDH | imidazoleglycerol-phosphate dehydratase |
| R_IGPS | indole-3-glycerol-phosphate synthase |
| R_ILETA | isoleucine transaminase |
| R_ILETRS | Isoleucyl-tRNA synthetase |
| R_ILEabcpp | L-isoleucine transport via ABC system (periplasm) |
| R_ILEt2rpp | L-isoleucine reversible transport via proton symport (periplasm) |
| R_ILEtex | L-isoleucine transport via diffusion (extracellular to periplasm) |
| R_IMPC | IMP cyclohydrolase |
| R_IMPD | IMP dehydrogenase |
| R_IMPtex | IMP transport via diffusion (extracellular to periplasm) |
| R_INDOLEt2pp | Indole transport via proton symport, irreversible (periplasm) |
| R_INDOLEt2rpp | Indole transport via proton symport, reversible (periplasm) |
| R_INDOLEtex | Indole transport via diffusion (extracellular to periplasm) |
| R_INOSTt4pp | Na+/myo-inositol symporter (periplasm) |
| R_INSH | Inosine hydrolase |
| R_INSK | insosine kinase |
| R_INSTtex | inositol transport via diffusion (extracellular to periplasm) |
| R_INSt2pp | inosine transport in via proton symport (periplasm) |
| R_INSt2rpp | inosine transport in via proton symport, reversible (periplasm) |
| R_INStex | inosine transport via diffusion (extracellular to periplasm) |
| R_IPDDI | isopentenyl-diphosphate D-isomerase |
| R_IPDPS | 1-hydroxy-2-methyl-2-(E)-butenyl 4-diphosphate reductase (ipdp) |
| R_IPMD | 3-isopropylmalate dehydrogenase |
| R_IPPMIa | 3-isopropylmalate dehydratase |
| R_IPPMIb | 2-isopropylmalate hydratase |
| R_IPPS | 2-isopropylmalate synthase |
| R_ISETACabcpp | isethionate transport via ABC system (periplasm) |
| R_ISETACtex | isethionate transport via diffusion (extracellular to periplasm) |
| R_K2L4Aabcpp | KDO(2)-lipid IV A transport via ABC system (periplasm) |
| R_K2L4Aabctex | KDO(2)-lipid IV A transport via ABC system (periplasm to extracellular) |
| R_KARA1 | ketol-acid reductoisomerase (2,3-dihydroxy-3-methylbutanoate) |
| R_KARA2 | ketol-acid reductoisomerase (2-Acetolactate) |
| R_KAS14 | beta-ketoacyl-ACP synthase |
| R_KAS15 | beta-ketoacyl-ACP synthase (2) |
| R_KDOCT2 | 3-deoxy-manno-octulosonate cytidylyltransferase |
| R_KDOPP | 3-deoxy-manno-octulosonate-8-phosphatase |
| R_KDOPS | 3-deoxy -D-manno-octulosonic -acid 8-phosphate synthase |
| R_KG6PDC | 3-keto-L-gulonate 6-phosphate decarboxylase |
| R_Kabcpp | Potassium ABC transporter (periplasm) |
| R_Kt2pp | potassium transport in via proton symport (periplasm) |
| R_Kt3pp | potassium transport out via proton antiport (periplasm) |
| R_Ktex | potassium transport via diffusion (extracellular to periplasm) |
| R_L_DASH_LACD2 | L-Lactate dehydrogenase (ubiquinone) |
| R_L_DASH_LACD3 | L-Lactate dehydrogenase (menaquinone) |
| R_L_DASH_LACt2rpp | L-lactate reversible transport via proton symport (periplasm) |
| R_L_DASH_LACtex | L-lactate transport via diffusion (extracellular to periplasm) |
| R_LA4NTpp | 4-amino-4-deoxy-L-arabinotransferase (LPS lipid A modification, periplasmic face of membrane)) |
| R_LACZ | b-galactosidase |
| R_LACZpp | b-galactosidase |
| R_LADGMDH | L-alanyl-gamma-D-glutamyl-meso-diaminopimelate hydrolase |
| R_LALADGLUtex | L-alanyl-D-glutamate transport via diffusion (extracellular to periplasm) |
| R_LALADGLUtpp | L-alanyl-D-glutamate transport in via proton symport (periplasm) |
| R_LALALGLUtex | L-alanyl-L-glutamate transport via diffusion (extracellular to periplasm) |
| R_LALALGLUtpp | L-alanyl-L-glutamate transport in via proton symport (periplasm) |
| R_LALDO2x | D-Lactaldehyde:NAD+ 1-oxidoreductase |
| R_LALDO3 | L-Lactaldehyde:NADP+ 1-oxidoreductase |
| R_LALGP | L-alanyl-gamma-L-glutamate peptidase |
| R_LCADi | lactaldehyde dehydrogenase |
| R_LCARR | lacaldehyde reductase (R-propane-1,2-diol forming) |
| R_LCARS | lacaldehyde reductase (S-propane-1,2-diol forming) |
| R_LCTSt3ipp | Lactose transport via proton aniport (periplasm) |
| R_LCTStex | Lactose transport via diffusion (extracellular to periplasm) |
| R_LCTStpp | Lactose transport via proton symport (periplasm) |
| R_LDH_D | D-lactate dehydrogenase |
| R_LDH_D2 | D-lactate dehydrogenase |
| R_LEUTAi | leucine transaminase (irreversible) |
| R_LEUTRS | Leucyl-tRNA synthetase |
| R_LEUabcpp | L-leucine transport via ABC system (periplasm) |
| R_LEUt2rpp | L-leucine reversible transport via proton symport (periplasm) |
| R_LEUtex | L-leucine transport via diffusion (extracellular to periplasm) |
| R_LGTHL | lactoylglutathione lyase |
| R_LIPACabcpp | lipid (cold) A transport via ABC system (periplasm) |
| R_LIPAHT2ex | core oligosaccharide lipid A:hexadecanoate transferase (n-C16:0) (extracellular membrane) |
| R_LIPAHTex | Lipid A:hexadecanoate transferase (n-C16:0) (extracellular membrane) |
| R_LIPAMPL | Lipoyl-adenylate protein ligase |
| R_LIPATPT | Lipoate-ATP adenylate transferase |
| R_LIPAabcpp | lipid A transport via ABC system (periplasm) |
| R_LIPAabctex | lipid A transport via ABC system (periplasm to extracellular) |
| R_LIPOCT | Lipoyl(octanoyl) transferase |
| R_LIPOS | Lipoate synthase |
| R_LIPOt2pp | Lipoate transport via proton symport (periplasm) |
| R_LIPOtex | lipoate transport via diffusion (extracellular to periplasm) |
| R_LPADSS | Lipid A disaccaride synthase |
| R_LPLIPAL1A120pp | Lysophospholipase L1 (2-acylglycerophosphotidate, n-C12:0) (periplasm) |
| R_LPLIPAL1A140pp | Lysophospholipase L1 (2-acylglycerophosphotidate, n-C14:0) (periplasm) |
| R_LPLIPAL1A141pp | Lysophospholipase L1 (2-acylglycerophosphotidate, n-C14:1) (periplasm) |
| R_LPLIPAL1A160pp | Lysophospholipase L1 (2-acylglycerophosphotidate, n-C16:0) (periplasm) |
| R_LPLIPAL1A161pp | Lysophospholipase L1 (2-acylglycerophosphotidate, n-C16:1) (periplasm) |
| R_LPLIPAL1A180pp | Lysophospholipase L1 (2-acylglycerophosphotidate, n-C18:0) (periplasm) |
| R_LPLIPAL1A181pp | Lysophospholipase L1 (2-acylglycerophosphotidate, n-C18:1) (periplasm) |
| R_LPLIPAL1E120pp | Lysophospholipase L1 (2-acylglycerophosphoethanolamine, n-C12:0) (periplasm) |
| R_LPLIPAL1E140pp | Lysophospholipase L1 (2-acylglycerophosphoethanolamine, n-C14:0) (periplasm) |
| R_LPLIPAL1E141pp | Lysophospholipase L1 (2-acylglycerophosphoethanolamine, n-C14:1) (periplasm) |
| R_LPLIPAL1E160pp | Lysophospholipase L1 (2-acylglycerophosphoethanolamine, n-C16:0) (periplasm) |
| R_LPLIPAL1E161pp | Lysophospholipase L1 (2-acylglycerophosphoethanolamine, n-C16:1) (periplasm) |
| R_LPLIPAL1E180pp | Lysophospholipase L1 (2-acylglycerophosphoethanolamine, n-C18:0) (periplasm) |
| R_LPLIPAL1E181pp | Lysophospholipase L1 (2-acylglycerophosphoethanolamine, n-C18:1) (periplasm) |
| R_LPLIPAL1G120pp | Lysophospholipase L1 (2-acylglycerophosphoglycerol, n-C12:0) (periplasm) |
| R_LPLIPAL1G140pp | Lysophospholipase L1 (2-acylglycerophosphoglycerol, n-C14:0) (periplasm) |
| R_LPLIPAL1G141pp | Lysophospholipase L1 (2-acylglycerophosphoglycerol, n-C14:1) (periplasm) |
| R_LPLIPAL1G160pp | Lysophospholipase L1 (2-acylglycerophosphoglycerol, n-C16:0) (periplasm) |
| R_LPLIPAL1G161pp | Lysophospholipase L1 (2-acylglycerophosphoglycerol, n-C16:1) (periplasm) |
| R_LPLIPAL1G180pp | Lysophospholipase L1 (2-acylglycerophosphoglycerol, n-C18:0) (periplasm) |
| R_LPLIPAL1G181pp | Lysophospholipase L1 (2-acylglycerophosphoglycerol, n-C18:1) (periplasm) |
| R_LPLIPAL2A120 | Lysophospholipase L2 (2-acylglycerophosphotidate, n-C12:0) |
| R_LPLIPAL2A140 | Lysophospholipase L2 (2-acylglycerophosphotidate, n-C14:0) |
| R_LPLIPAL2A141 | Lysophospholipase L2 (2-acylglycerophosphotidate, n-C14:1) |
| R_LPLIPAL2A160 | Lysophospholipase L2 (2-acylglycerophosphotidate, n-C16:0) |
| R_LPLIPAL2A161 | Lysophospholipase L2 (2-acylglycerophosphotidate, n-C16:1) |
| R_LPLIPAL2A180 | Lysophospholipase L2 (2-acylglycerophosphotidate, n-C18:0) |
| R_LPLIPAL2A181 | Lysophospholipase L2 (2-acylglycerophosphotidate, n-C18:1) |
| R_LPLIPAL2ATE120 | Lysophospholipase L2 (acyltransferase, 2-acyl-glycerophosphoethanolamine, n-C12:0) |
| R_LPLIPAL2ATE140 | Lysophospholipase L2 (acyltransferase, 2-acyl-glycerophosphoethanolamine, n-C14:0) |
| R_LPLIPAL2ATE141 | Lysophospholipase L2 (acyltransferase, 2-acyl-glycerophosphoethanolamine, n-C14:1) |
| R_LPLIPAL2ATE160 | Lysophospholipase L2 (acyltransferase, 2-acyl-glycerophosphoethanolamine, n-C16:0) |
| R_LPLIPAL2ATE161 | Lysophospholipase L2 (acyltransferase, 2-acyl-glycerophosphoethanolamine, n-C16:1) |
| R_LPLIPAL2ATE180 | Lysophospholipase L2 (acyltransferase, 2-acyl-glycerophosphoethanolamine, n-C18:0) |
| R_LPLIPAL2ATE181 | Lysophospholipase L2 (acyltransferase, 2-acyl-glycerophosphoethanolamine, n-C18:1) |
| R_LPLIPAL2ATG120 | Lysophospholipase L2 (acyltransferase, 2-acyl-glycerophosphoglycerol, n-C12:0) |
| R_LPLIPAL2ATG140 | Lysophospholipase L2 (acyltransferase, 2-acyl-glycerophosphoglycerol, n-C14:0) |
| R_LPLIPAL2ATG141 | Lysophospholipase L2 (acyltransferase, 2-acyl-glycerophosphoglycerol, n-C14:1) |
| R_LPLIPAL2ATG160 | Lysophospholipase L2 (acyltransferase, 2-acyl-glycerophosphoglycerol, n-C16:0) |
| R_LPLIPAL2ATG161 | Lysophospholipase L2 (acyltransferase, 2-acyl-glycerophosphoglycerol, n-C16:1) |
| R_LPLIPAL2ATG180 | Lysophospholipase L2 (acyltransferase, 2-acyl-glycerophosphoglycerol, n-C18:0) |
| R_LPLIPAL2ATG181 | Lysophospholipase L2 (acyltransferase, 2-acyl-glycerophosphoglycerol, n-C18:1) |
| R_LPLIPAL2E120 | Lysophospholipase L2 (2-acylglycerophosphoethanolamine, n-C12:0) |
| R_LPLIPAL2E140 | Lysophospholipase L2 (2-acylglycerophosphoethanolamine, n-C14:0) |
| R_LPLIPAL2E141 | Lysophospholipase L2 (2-acylglycerophosphoethanolamine, n-C14:1) |
| R_LPLIPAL2E160 | Lysophospholipase L2 (2-acylglycerophosphoethanolamine, n-C16:0) |
| R_LPLIPAL2E161 | Lysophospholipase L2 (2-acylglycerophosphoethanolamine, n-C16:1) |
| R_LPLIPAL2E180 | Lysophospholipase L2 (2-acylglycerophosphoethanolamine, n-C18:0) |
| R_LPLIPAL2E181 | Lysophospholipase L2 (2-acylglycerophosphoethanolamine, n-C18:1) |
| R_LPLIPAL2G120 | Lysophospholipase L2 (2-acylglycerophosphoglycerol, n-C12:0) |
| R_LPLIPAL2G140 | Lysophospholipase L2 (2-acylglycerophosphoglycerol, n-C14:0) |
| R_LPLIPAL2G141 | Lysophospholipase L2 (2-acylglycerophosphoglycerol, n-C14:1) |
| R_LPLIPAL2G160 | Lysophospholipase L2 (2-acylglycerophosphoglycerol, n-C16:0) |
| R_LPLIPAL2G161 | Lysophospholipase L2 (2-acylglycerophosphoglycerol, n-C16:1) |
| R_LPLIPAL2G180 | Lysophospholipase L2 (2-acylglycerophosphoglycerol, n-C18:0) |
| R_LPLIPAL2G181 | Lysophospholipase L2 (2-acylglycerophosphoglycerol, n-C18:1) |
| R_LSERDHr | L-serine dehydrogenase |
| R_LYSDC | lysine decarboxylase |
| R_LYSTRS | Lysyl-tRNA synthetase |
| R_LYSabcpp | L-lysine transport via ABC system (periplasm) |
| R_LYSt2pp | L-lysine transport in via proton symport (periplasm) |
| R_LYSt3pp | L-lysine transport out via proton antiport (cytoplasm to periplasm) |
| R_LYStex | L-lysine transport via diffusion (extracellular to periplasm) |
| R_LYXI | Lyxose isomerase |
| R_LYXt2pp | L-Lyxose transport via proton symport (periplasm) |
| R_LYXtex | L-Lyxose transport via diffusion (extracellular to periplasm) |
| R_M1PD | mannitol-1-phosphate 5-dehydrogenase |
| R_MACPD | Malonyl-ACP decarboxylase |
| R_MALCOAMT | Malonyl-CoA methyltransferase |
| R_MALDDH | malate decarboxylating oxidoreductase (decarboxylating) |
| R_MALDt2_2pp | D-Malate transport via proton symport (2 H) (periplasm) |
| R_MALDtex | D-Malate transport via diffusion (extracellular to periplasm) |
| R_MALS | malate synthase |
| R_MALTATr | maltose O-acetyltransferase |
| R_MALTHXabcpp | maltohexaose transport via ABC system (periplasm) |
| R_MALTHXtexi | maltohexaose transport via diffusion (extracellular to periplasm) irreversible |
| R_MALTPTabcpp | maltopentaose transport via ABC system (periplasm) |
| R_MALTPTtexi | maltopentaoseMaltotriose transport via diffusion (extracellular to periplasm) irreversible |
| R_MALTTRabcpp | Maltotriose transport via ABC system (periplasm) |
| R_MALTTRtexi | Maltotriose transport via diffusion (extracellular to periplasm) irreversible |
| R_MALTTTRabcpp | maltotetraose transport via ABC system (periplasm) |
| R_MALTTTRtexi | maltotetraoseMaltotriose transport via diffusion (extracellular to periplasm) irreversible |
| R_MALTabcpp | maltose transport via ABC system (periplasm) |
| R_MALTptspp | maltose transport via PEP:Pyr PTS (periplasm) |
| R_MALTtexi | maltoseMaltotriose transport via diffusion (extracellular to periplasm) irreversible |
| R_MALt2_2pp | Malate transport via proton symport (2 H) (periplasm) |
| R_MALt2_3pp | Malate transport via proton symport (3 H) (periplasm) |
| R_MALt3pp | L-malate transport out via proton antiport (periplasm) |
| R_MALtex | Malate transport via diffusion (extracellular to periplasm) |
| R_MAN1PT2 | mannose-1-phosphate guanylyltransferase (GDP) |
| R_MAN6PI | mannose-6-phosphate isomerase |
| R_MAN6Pt6_2pp | Mannose-6-phosphate transport via phosphate antiport (periplasm) |
| R_MAN6Ptex | Mannose 6-phosphate transport via diffusion (extracellular to periplasm) |
| R_MANAO | Mannonate oxidoreductase |
| R_MANGLYCptspp | 2-O-alpha-mannosyl-D-glycerate transport via PEP:Pyr PTS (periplasm) |
| R_MANGLYCtex | 2-O-alpha-mannosyl-D-glycerate transport via diffusion (extracellular to periplasm) |
| R_MANPGH | 2-O-alpha-mannosyl-6-phosphate-D-glycerate hydrolase |
| R_MANptspp | D-mannose transport via PEP:Pyr PTS (periplasm) |
| R_MANtex | D-mannose transport via diffusion (extracellular to periplasm) |
| R_MCITD | 2-methylcitrate dehydratase |
| R_MCITL2 | methylisocitrate lyase |
| R_MCITS | 2-methylcitrate synthase |
| R_MCOATA | Malonyl-CoA-ACP transacylase |
| R_MCPST | 3-mercaptopyruvate sulfurtransferase |
| R_MCTP1App | murein crosslinking transpeptidase 1A:(A2pm->D-ala) (periplasm) |
| R_MCTP1Bpp | murein crosslinking transpeptidase 1B:(A2pm->A2pm) (periplasm) |
| R_MCTP2App | murein crosslinking transpeptidase 1A:(A2pm->D-ala) (periplasm) |
| R_MDDCP1pp | murein D,D-carboxypeptidase (murein5px4p) (periplasm) |
| R_MDDCP2pp | murein D,D-carboxypeptidase (murein5px4px4p) (periplasm) |
| R_MDDCP3pp | murein D,D-carboxypeptidase (murein5p5p) (periplasm) |
| R_MDDCP4pp | murein D,D-carboxypeptidase (murein5p4p) (periplasm) |
| R_MDDCP5pp | murein D,D-carboxypeptidase (murein5p3p) (periplasm) |
| R_MDDEP1pp | murein D,D-endopeptidase (murein4px4p) (periplasm) |
| R_MDDEP2pp | murein D,D-endopeptidase (murein3px4p) (periplasm) |
| R_MDDEP3pp | murein D,D-endopeptidase (murein5px4p) (periplasm) |
| R_MDDEP4pp | murein D,D-endopeptidase (murein4px4px4p) (periplasm) |
| R_MDH | malate dehydrogenase |
| R_MDH2 | Malate dehydrogenase (ubiquinone 8 as acceptor) |
| R_MDH3 | Malate dehydrogenase (menaquinone 8 as acceptor) |
| R_ME1 | malic enzyme (NAD) |
| R_ME2 | malic enzyme (NADP) |
| R_MECDPDH5 | 2C-methyl-D-erythritol 2,4 cyclodiphosphate dehydratase |
| R_MECDPS | 2-C-methyl-D-erythritol 2,4-cyclodiphosphate synthase |
| R_MELIBt2pp | melibiose transport in via symport (periplasm) |
| R_MELIBt3ipp | melibiose transport in via antiport (periplasm) |
| R_MELIBtex | melibiose transport via diffusion (extracellular to periplasm) |
| R_MEOHtex | Methanol transport via diffusion (extracellular to periplasm) |
| R_MEOHtrpp | Methanol reversible transport via diffusion (periplasm) |
| R_MEPCT | 2-C-methyl-D-erythritol 4-phosphate cytidylyltransferase |
| R_METAT | methionine adenosyltransferase |
| R_METDabcpp | D-methionine transport via ABC system (periplasm) |
| R_METDtex | D-methionine transport via diffusion (extracellular to periplasm) |
| R_METOX1s | methionine oxidation (spontaneous) |
| R_METOX2s | methionine oxidation 2 (spontaneous) |
| R_METS | methionine synthase |
| R_METSOX1abcpp | L-methionine S-oxide transport via ABC system (periplasm) |
| R_METSOX1tex | L-methionine S-oxide diffusion (extracellular) |
| R_METSOX2abcpp | L-methionine R-oxide transport via ABC system (periplasm) |
| R_METSOX2tex | L-methionine R-oxide diffusion (extracellular) |
| R_METSOXR1 | L-methionine-S-oxide reductase |
| R_METSOXR2 | L-methionine-R-sulfoxide reductase |
| R_METTRS | Methionyl-tRNA synthetase |
| R_METabcpp | L-methionine transport via ABC system (periplasm) |
| R_METtex | L-methionine transport via diffusion (extracellular to periplasm) |
| R_MG2t3_2pp | magnesium (Mg+2) transport in/out via proton antiport (periplasm) |
| R_MG2tex | magnesium (Mg+2) transport via diffusion (extracellular to periplasm) |
| R_MG2tpp | magnesium (+2) transport in via diffusion |
| R_MG2uabcpp | Magnesium (Mg+2) ABC transporter (ubtake, periplasm) |
| R_MGSA | methylglyoxal synthase |
| R_MI1PP | myo-inositol 1-phosphatase |
| R_MICITDr | 2-methylisocitrate dehydratase |
| R_MINCYCtex | Minocycline transport via diffusion (extracellular to periplasm) |
| R_MINCYCtpp | Minocycline transport via TolC system |
| R_MINOHPtexi | myo-inositol phosphate transport via diffusion (extracellular to periplasm) |
| R_MLDCP1App | murein L,D-carboxypeptidase (murein5px4p) (periplasm) |
| R_MLDCP1Bpp | murein L,D-carboxypeptidase (murein4p4p) (periplasm) |
| R_MLDCP2App | murein L,D-carboxypeptidase (murein5p5p) (periplasm) |
| R_MLDCP2Bpp | murein L,D-carboxypeptidase (murein4p3p) (periplasm) |
| R_MLDCP3App | murein L,D-carboxypeptidase (murein5px3p) (periplasm) |
| R_MLDEP1pp | murein L,D-endopeptidase (murein3px3p) (periplasm) |
| R_MLDEP2pp | murein L,D-endopeptidase (murein5px3p) (periplasm) |
| R_MLTG1 | Maltodextrin glucosidase (maltotriose) |
| R_MLTG2 | Maltodextrin glucosidase (maltotetraose) |
| R_MLTG3 | Maltodextrin glucosidase (maltopentaose) |
| R_MLTG4 | Maltodextrin glucosidase (maltohexaose) |
| R_MLTG5 | Maltodextrin glucosidase (maltoheptaose) |
| R_MLTGY1pp | murein lytic transglycosylase (murein4p4p) (periplasm) |
| R_MLTGY2pp | murein lytic transglycosylase (murein4p3p) (periplasm) |
| R_MLTGY3pp | murein lytic transglycosylase (murein3p3p) (periplasm) |
| R_MLTGY4pp | murein lytic transglycosylase (murein4px4p4p) (periplasm) |
| R_MLTP1 | Maltodextrin phosphorylase (maltopentaose) |
| R_MLTP2 | Maltodextrin phosphorylase (maltohexaose) |
| R_MLTP3 | Maltodextrin phosphorylase (maltoheptaose) |
| R_MMCD | Methylmalonyl-CoA decarboxylase |
| R_MMETt2pp | S-methylmethionine permease (periplasm) |
| R_MMETtex | S-methyl-L-methionine transport via diffusion (extracellular to periplasm) |
| R_MMM | Methylmalonyl-CoA mutase |
| R_MN2t3pp | manganese (Mn+2) transport out via proton antiport (periplasm) |
| R_MN2tpp | manganese transport in via permease (no H+) |
| R_MN6PP | mannose 6-phosphate phosphatase |
| R_MNLptspp | mannitol transport via PEP:Pyr PTS (periplasm) |
| R_MNLtex | mannitol transport via diffusion (extracellular to periplasm) |
| R_MNNH | D-mannonate hydrolyase |
| R_MNt2pp | manganese (Mn+2) transport in via proton symport (periplasm) |
| R_MNtex | Manganese (Mn+2) transport via diffusion (extracellular to periplasm) |
| R_MOADSUx | MoaD sulfuration (nadh, assumed) |
| R_MOAT | 3-deoxy-D-manno-octulosonic acid transferase |
| R_MOAT2 | 3-deoxy-D-manno-octulosonic acid transferase |
| R_MOAT3C | 3-deoxy-D-manno-octulosonic acid transferase III (LPS core biosynthesis) |
| R_MOBDabcpp | molybdate transport via ABC system (periplasm) |
| R_MOBDtex | molybdate transport via diffusion (extracellular to periplasm) |
| R_MOCDS | molybdopterin cytidine dinucleotide synthase |
| R_MOCOS | molybdenum cofactor synthase |
| R_MOGDS | molybdopterin guanine dinucleotide synthase |
| R_MOHMT | 3-methyl-2-oxobutanoate hydroxymethyltransferase |
| R_MOX | malate oxidase |
| R_MPTAT | molybdopterin adenylyltransferase |
| R_MPTG | murein polymerizing transglycosylase |
| R_MPTG2 | murein polymerizing transglycosylase 2 (three linked units) |
| R_MPTS | molybdopterin synthase |
| R_MPTSS | molybdopterin synthase sulfurylase |
| R_MSAR | malonic semialdehyde reductase |
| R_MSO3abcpp | methanesulfonate transport via ABC system (periplasm) |
| R_MSO3tex | methanesulfonate transport via diffusion (extracellular to periplasm) |
| R_MTAN | methylthioadenosine nucleosidase |
| R_MTHFC | methenyltetrahydrofolate cyclohydrolase |
| R_MTHFD | methylenetetrahydrofolate dehydrogenase (NADP) |
| R_MTHFR2 | 5,10-methylenetetrahydrofolate reductase (NADH) |
| R_MTHTHFSs | (2R,4S)-2-methyl-2,3,3,4-tetrahydroxytetrahydrofuran synthesis (spontaneous) |
| R_MTRPOX | N-methyltryptophan oxidase |
| R_N2Otex | nitrious oxide transport via diffusion (extracellular to periplasm) |
| R_N2Otpp | nitrious oxide transport (diffusion) |
| R_NACODA | N-acetylornithine deacetylase |
| R_NACtex | Nicotinic acid transport via diffusion (extracellular to periplasm) |
| R_NACtpp | Nicotinic acid uptake (periplasm) |
| R_NADDP | NAD diphosphatase |
| R_NADH10 | NADH dehydrogenase (menaquinone-8 & 0 protons) |
| R_NADH16pp | NADH dehydrogenase (ubiquinone-8 & 3 protons) (periplasm) |
| R_NADH17pp | NADH dehydrogenase (menaquinone-8 & 3 protons) (periplasm) |
| R_NADH18pp | NADH dehydrogenase (demethylmenaquinone-8 & 3 protons) (periplasm) |
| R_NADH5 | NADH dehydrogenase (ubiquinone-8 ) |
| R_NADH9 | NADH dehydrogenase (demethylmenaquinone-8 & 0 protons) |
| R_NADK | NAD kinase |
| R_NADN | NAD nucleosidase |
| R_NADPHQR2 | NADPH Quinone Reductase (Ubiquinone-8) |
| R_NADPHQR3 | NADPH Quinone Reductase (Menaquinone-8) |
| R_NADPHQR4 | NADPH Quinone Reductase (2-Demethylmenaquinone-8) |
| R_NADPPPS | NADP phosphatase |
| R_NADS1 | NAD synthase (nh3) |
| R_NADTRHD | NAD transhydrogenase |
| R_NAMNPP | nicotinic acid mononucleotide pyrophosphorylase |
| R_NAt3_1p5pp | sodium proton antiporter (H:NA is 1.5) (periplasm) |
| R_NAt3_2pp | sodium proton antiporter (H:NA is 2) (periplasm) |
| R_NAt3pp | sodium transport out via proton antiport (cytoplasm to periplasm) |
| R_NAtex | sodium transport via diffusion (extracellular to periplasm) |
| R_NDPK1 | nucleoside-diphosphate kinase (ATP:GDP) |
| R_NDPK2 | nucleoside-diphosphate kinase (ATP:UDP) |
| R_NDPK3 | nucleoside-diphosphate kinase (ATP:CDP) |
| R_NDPK4 | nucleoside-diphosphate kinase (ATP:dTDP) |
| R_NDPK5 | nucleoside-diphosphate kinase (ATP:dGDP) |
| R_NDPK6 | nucleoside-diphosphate kinase (ATP:dUDP) |
| R_NDPK7 | nucleoside-diphosphate kinase (ATP:dCDP) |
| R_NDPK8 | nucleoside-diphosphate kinase (ATP:dADP) |
| R_NH4tex | ammonia transport via diffusion (extracellular to periplasm) |
| R_NH4tpp | ammonia reversible transport (periplasm) |
| R_NHFRBO | NADH:flavorubredoxin oxidoreductase |
| R_NI2abcpp | Nickle (Ni+2) ABC transporter (periplasm) |
| R_NI2t3pp | nickle (Ni+2) transport out via proton antiport (periplasm) |
| R_NI2tex | nickel transport via diffusion (extracellular to periplasm) |
| R_NI2tpp | nickel (+2) transport in via permease (no H+) |
| R_NI2uabcpp | nickel transport via ABC system (uptake, periplasm) |
| R_NMNAT | nicotinamide-nucleotide adenylyltransferase |
| R_NMNDA | nicotinamide-nucleotide amidase |
| R_NMNN | NMN nucleosidase |
| R_NMNPtpp | NMN permease (periplasm) |
| R_NMNt7pp | NMN transport via NMN glycohydrolase (periplasm) |
| R_NMNtex | NMN transport via diffusion (extracellular to periplasm) |
| R_NNAM | nicotinamidase |
| R_NNATr | nicotinate-nucleotide adenylyltransferase |
| R_NNDMBRT | Nicotinate-nucleotide dimethylbenzimidazole phosphoribosyltransferase |
| R_NNDPR | nicotinate-nucleotide diphosphorylase (carboxylating) |
| R_NO2t2rpp | nitrite transport in via proton symport, reversible (periplasm) |
| R_NO2tex | nitrite transport via diffusion (extracellular to periplasm) |
| R_NO3R1bpp | Nitrate reductase (Ubiquinol-8) |
| R_NO3R1pp | Nitrate reductase (Ubiquinol-8) (periplasm) |
| R_NO3R2bpp | Nitrate reductase (Menaquinol-8) (periplasm) |
| R_NO3R2pp | Nitrate reductase (Menaquinol-8) (periplasm) |
| R_NO3t7pp | nitrate transport in via nitrite antiport (periplasm) |
| R_NO3tex | nitrate transport via diffusion (extracellular to periplasm) |
| R_NODOx | nitric oxide dioxygenase |
| R_NODOy | nitric oxide dioxygenase |
| R_NOVBCNtex | Novobiocin transport via diffusion (extracellular to periplasm) |
| R_NOVBCNtpp | Novobiocin transport via TolC system |
| R_NOtex | nitric oxide transport via diffusion (extracellular to periplasm) |
| R_NOtpp | NO transport (diffusion) |
| R_NTD1 | 5'-nucleotidase (dUMP) |
| R_NTD10 | 5'-nucleotidase (XMP) |
| R_NTD10pp | 5'-nucleotidase (XMP) |
| R_NTD11 | 5'-nucleotidase (IMP) |
| R_NTD11pp | 5'-nucleotidase (IMP) |
| R_NTD12 | 5'-nucleotidase (dIMP) |
| R_NTD12pp | 5'-nucleotidase (dIMP) (periplasm) |
| R_NTD1pp | 5'-nucleotidase (dUMP) |
| R_NTD2 | 5'-nucleotidase (UMP) |
| R_NTD2pp | 5'-nucleotidase (UMP) |
| R_NTD3 | 5'-nucleotidase (dCMP) |
| R_NTD3pp | 5'-nucleotidase (dCMP) |
| R_NTD4 | 5'-nucleotidase (CMP) |
| R_NTD4pp | 5'-nucleotidase (CMP) |
| R_NTD5 | 5'-nucleotidase (dTMP) |
| R_NTD5pp | 5'-nucleotidase (dTMP) |
| R_NTD6 | 5'-nucleotidase (dAMP) |
| R_NTD6pp | 5'-nucleotidase (dAMP) |
| R_NTD7 | 5'-nucleotidase (AMP) |
| R_NTD7pp | 5'-nucleotidase (AMP) |
| R_NTD8 | 5'-nucleotidase (dGMP) |
| R_NTD8pp | 5'-nucleotidase (dGMP) |
| R_NTD9 | 5'-nucleotidase (GMP) |
| R_NTD9pp | 5'-nucleotidase (GMP) |
| R_NTP1 | nucleoside-triphosphatase (ATP) |
| R_NTP10 | nucleoside-triphosphatase (ITP) |
| R_NTP11 | nucleoside-triphosphatase (dITP) |
| R_NTP12 | nucleoside-triphosphatase (XTP) |
| R_NTP3 | nucleoside-triphosphatase (GTP) |
| R_NTP3pp | nucleoside-triphosphatase (GTP) (periplasm) |
| R_NTP5 | nucleoside-triphosphatase (CTP) |
| R_NTPP1 | Nucleoside triphosphate pyrophosphorylase (dgtp) |
| R_NTPP10 | Nucleoside triphosphate pyrophosphorylase (ditp) |
| R_NTPP11 | Nucleoside triphosphate pyrophosphorylase (xtp) |
| R_NTPP2 | Nucleoside triphosphate pyrophosphorylase (gtp) |
| R_NTPP3 | Nucleoside triphosphate pyrophosphorylase (dctp) |
| R_NTPP4 | Nucleoside triphosphate pyrophosphorylase (ctp) |
| R_NTPP5 | Nucleoside triphosphate pyrophosphorylase (datp) |
| R_NTPP6 | Nucleoside triphosphate pyrophosphorylase (atp) |
| R_NTPP7 | Nucleoside triphosphate pyrophosphorylase (dttp) |
| R_NTPP8 | Nucleoside triphosphate pyrophosphorylase (utp) |
| R_NTPP9 | Nucleoside triphosphate pyrophosphorylase (itp) |
| R_NTPTP1 | Nucleoside triphosphate tripolyhydrolase |
| R_NTPTP2 | Nucleoside triphosphate tripolyhydrolase |
| R_NTRIR2x | nitrite Reductase (NADH) |
| R_NTRIR3pp | nitrite Reductase (Ubiquinole-8, periplasm) |
| R_NTRIR4pp | nitrite Reductase (Menaquinole-8, periplasm) |
| R_O16A4COLIPAabctex | O16 antigen (x4) core oligosaccharide lipid A transport via ABC system (periplasm to extracellular) |
| R_O16A4Lpp | O16 anitgen (x4) ligase (periplasm) |
| R_O16AP1pp | O16 antigen polymerase (periplasm) |
| R_O16AP2pp | O16 antigen polymerase (periplasm) |
| R_O16AP3pp | O16 antigen polymerase (periplasm) |
| R_O16AT | rhamanosyl-N-acetylglucosamyl-undecaprenyl diphosphate O-acetyltransferase (LPS O16 antigen biosynthesis) |
| R_O16AUNDtpp | O16 antigen (flippase, cytoplasm to periplasm) |
| R_O16GALFT | galactofuranosyltransferase (LPS O16 antigen biosynthesis) |
| R_O16GLCT1 | glucosyltransferase I (LPS O16 antigen biosynthesis) |
| R_O16GLCT2 | glucosyltransferase II (LPS O16 antigen biosynthesis) |
| R_O2Stex | superoxide anion transport via diffusion (extracellular to periplasm) |
| R_O2tex | oxygen transport via diffusion (extracellular to periplasm) |
| R_O2tpp | o2 transport via diffusion (periplasm) |
| R_OAADC | oxaloacetate decarboxylase |
| R_OBTFL | 2-Oxobutanoate formate lyase |
| R_OCBT | ornithine carbamoyltransferase |
| R_OCDCAtexi | Octadecanoate transport via facilitated irreversible diffusion (extracellular to periplasm) |
| R_OCDCEAtexi | Octadecenoate (n-C18:1) transport via facilitated irreversible diffusion (extracellular to periplasm) |
| R_OCTAtex | Octanoate transport via diffusion (extracellular to periplasm) |
| R_OCTDPS | Octaprenyl pyrophosphate synthase |
| R_OCTNLL | Octanoate non-lipoylated apo domain ligase |
| R_OGMEACPD | 3-Oxo-glutaryl-[ACP] methyl ester dehydratase |
| R_OGMEACPR | 3-Oxo-glutaryl-[ACP] methyl ester reductase |
| R_OGMEACPS | 3-Oxo-glutaryl-[ACP] methyl ester synthase |
| R_OHPBAT | O-Phospho-4-hydroxy-L-threonine:2-oxoglutarate aminotransferase |
| R_OHPHM | 2-octaprenyl-6-hydroxyphenol methylase |
| R_OMBZLM | 2-Octaprenyl-6-methoxy-benzoquinol methylase |
| R_OMCDC | 2-Oxo-4-methyl-3-carboxypentanoate decarboxylation |
| R_OMMBLHX | 2-Octaprenyl-3-methyl-6-methoxy-1,4-benzoquinol hydroxylase |
| R_OMMBLHX3 | 2-Octaprenyl-3-methyl-6-methoxy-1,4-benzoquinol hydroxylase (anaerobic) |
| R_OMPDC | orotidine-5'-phosphate decarboxylase |
| R_OMPHHX | 2-octaprenyl-6-methoxyphenol hydroxylase |
| R_OMPHHX3 | 2-octaprenyl-6-methoxyphenol hydroxylase (anaerobic) |
| R_OP4ENH | 2-oxopent-4-enoate hydratase |
| R_OPHBDC | Octaprenyl-hydroxybenzoate decarboxylase |
| R_OPHHX | 2-Octaprenylphenol hydroxylase |
| R_OPHHX3 | 2-Octaprenylphenol hydroxylase (anaerobic) |
| R_OPMEACPD | 3-Oxo-pimeloyl-[ACP] methyl ester dehydratase |
| R_OPMEACPR | 3-Oxo-pimeloyl-[ACP] methyl ester reductase |
| R_OPMEACPS | 3-Oxo-pimeloyl-[ACP] methyl ester synthase |
| R_ORNDC | Ornithine Decarboxylase |
| R_ORNabcpp | ornithine transport via ABC system (periplasm) |
| R_ORNtex | ornithine transport via diffusion (extracellular to periplasm) |
| R_OROTt2_2pp | Orotate transport via proton symport (2 H) (periplasm) |
| R_OROTtex | Orotate transport via diffusion (extracellular to periplasm) |
| R_ORPT | orotate phosphoribosyltransferase |
| R_OXAMTC | oxamate transcarbamoylase |
| R_OXCDC | Oxalyl-CoA decarboxylase |
| R_OXCOAHDH | oxepin-CoA hydrolase/ 3-oxo-5,6-dehydrosuberyl-CoA semialdehyde dehydrogenase (NADP+) |
| R_OXDHCOAT | 3-oxo-5,6-dehydrosuberyl-CoA thiolase |
| R_P5CD | 1-pyrroline-5-carboxylate dehydrogenase |
| R_P5CR | pyrroline-5-carboxylate reductase |
| R_PA120abcpp | phosphatidate transport via ABC system (n-C12:0, periplasm) |
| R_PA140abcpp | phosphatidate transport via ABC system (n-C14:0, periplasm) |
| R_PA141abcpp | phosphatidate transport via ABC system (n-C14:1, periplasm) |
| R_PA160abcpp | phosphatidate transport via ABC system (n-C16:0, periplasm) |
| R_PA161abcpp | phosphatidate transport via ABC system (n-C16:1, periplasm) |
| R_PA180abcpp | phosphatidate transport via ABC system (n-C18:0, periplasm) |
| R_PA181abcpp | phosphatidate transport via ABC system (n-C18:1, periplasm) |
| R_PACALDt2rpp | phenylacetaldehyde reversible transport via proton symport (periplasm) |
| R_PACALDtex | phenethylacetaldehyde transport via diffusion (extracellular to periplasm) |
| R_PACCOAE | ring 1,2-phenylacetyl-CoA epoxidase (NADPH) |
| R_PACCOAL | phenylacetate-CoA ligase |
| R_PANTS | pantothenate synthase |
| R_PAPA120 | Phosphatidate phosphatase (n-C12:0) |
| R_PAPA120pp | Phosphatidate phosphatase (periplasmic, n-C12:0) |
| R_PAPA140 | Phosphatidate phosphatase (n-C14:0) |
| R_PAPA140pp | Phosphatidate phosphatase (periplasmic, n-C14:0) |
| R_PAPA141 | Phosphatidate phosphatase (n-C14:1) |
| R_PAPA141pp | Phosphatidate phosphatase (periplasmic, n-C14:1) |
| R_PAPA160 | Phosphatidate phosphatase (n-C16:0) |
| R_PAPA160pp | Phosphatidate phosphatase (periplasmic, n-C16:0) |
| R_PAPA161 | Phosphatidate phosphatase (n-C16:1) |
| R_PAPA161pp | Phosphatidate phosphatase (periplasmic, n-C16:1) |
| R_PAPA180 | Phosphatidate phosphatase (n-C18:0) |
| R_PAPA180pp | Phosphatidate phosphatase (periplasmic, n-C18:0) |
| R_PAPA181 | Phosphatidate phosphatase (n-C18:1) |
| R_PAPA181pp | Phosphatidate phosphatase (periplasmic, n-C18:1) |
| R_PAPPT3 | phospho-N-acetylmuramoyl-pentapeptide-transferase (meso-2,6-diaminopimelate) |
| R_PAPSR | phosphoadenylyl-sulfate reductase (thioredoxin) |
| R_PAPSR2 | phosphoadenylyl-sulfate reductase (glutaredoxin) |
| R_PDE1 | 3',5'-cyclic-nucleotide phosphodiesterase |
| R_PDE4 | 3',5'-cyclic-nucleotide phosphodiesterase |
| R_PDH | pyruvate dehydrogenase |
| R_PDX5PO2 | pyridoxine 5'-phosphate oxidase (anaerboic |
| R_PDX5POi | pyridoxine 5'-phosphate oxidase |
| R_PDX5PS | Pyridoxine 5'-phosphate synthase |
| R_PDXPP | Pyridoxine 5-phosphate phosphatase |
| R_PE120abcpp | phosphatidylethanolamine transport via ABC system (n-C12:0, periplasm) |
| R_PE140abcpp | phosphatidylethanolamine transport via ABC system (n-C14:0, periplasm) |
| R_PE141abcpp | phosphatidylethanolamine transport via ABC system (n-C14:1, periplasm) |
| R_PE160abcpp | phosphatidylethanolamine transport via ABC system (n-C16:0, periplasm) |
| R_PE161abcpp | phosphatidylethanolamine transport via ABC system (n-C16:1, periplasm) |
| R_PE180abcpp | phosphatidylethanolamine transport via ABC system (n-C18:0, periplasm) |
| R_PE181abcpp | phosphatidylethanolamine transport via ABC system (n-C18:1, periplasm) |
| R_PEAMNOpp | Phenethylamine oxidase |
| R_PEAMNtex | phenethylamine transport via diffusion (extracellular to periplasm) |
| R_PERD | Erythronate 4-phosphate (4per) dehydrogenase |
| R_PETNT161pp | phosphoethanolamine transferase (c-C16:1) |
| R_PETNT181pp | phosphoethanolamine transferase (c-C16:1) |
| R_PFK | phosphofructokinase |
| R_PFK_2 | Phosphofructokinase |
| R_PFK_3 | phosphofructokinase (s7p) |
| R_PFL | pyruvate formate lyase |
| R_PG120abcpp | phosphatidylglycerol transport via ABC system (n-C12:0, periplasm) |
| R_PG140abcpp | phosphatidylglycerol transport via ABC system (n-C14:0, periplasm) |
| R_PG141abcpp | phosphatidylglycerol transport via ABC system (n-C14:1, periplasm) |
| R_PG160abcpp | phosphatidylglycerol transport via ABC system (n-C16:0, periplasm) |
| R_PG161abcpp | phosphatidylglycerol transport via ABC system (n-C16:1, periplasm) |
| R_PG180abcpp | phosphatidylglycerol transport via ABC system (n-C18:0, periplasm) |
| R_PG181abcpp | phosphatidylglycerol transport via ABC system (n-C18:1, periplasm) |
| R_PGAMT | phosphoglucosamine mutase |
| R_PGCD | phosphoglycerate dehydrogenase |
| R_PGI | glucose-6-phosphate isomerase |
| R_PGK | phosphoglycerate kinase |
| R_PGL | 6-phosphogluconolactonase |
| R_PGLYCP | Phosphoglycolate phosphatase |
| R_PGM | phosphoglycerate mutase |
| R_PGMT | phosphoglucomutase |
| R_PGP120abcpp | phosphatidylglycerophosphate transport via ABC system (n-C12:0, periplasm) |
| R_PGP140abcpp | phosphatidylglycerophosphate transport via ABC system (n-C14:0, periplasm) |
| R_PGP141abcpp | phosphatidylglycerophosphate transport via ABC system (n-C14:1, periplasm) |
| R_PGP160abcpp | phosphatidylglycerophosphate transport via ABC system (n-C16:0, periplasm) |
| R_PGP161abcpp | phosphatidylglycerophosphate transport via ABC system (n-C16:1, periplasm) |
| R_PGP180abcpp | phosphatidylglycerophosphate transport via ABC system (n-C18:0, periplasm) |
| R_PGP181abcpp | phosphatidylglycerophosphate transport via ABC system (n-C18:1, periplasm) |
| R_PGPP120 | phosphatidylglycerol phosphate phosphatase (n-C14:0) |
| R_PGPP120pp | phosphatidylglycerol phosphate phosphatase (periplasm, n-C14:0) |
| R_PGPP140 | phosphatidylglycerol phosphate phosphatase (n-C14:0) |
| R_PGPP140pp | phosphatidylglycerol phosphate phosphatase (periplasm, n-C14:0) |
| R_PGPP141 | phosphatidylglycerol phosphate phosphatase (n-C14:1) |
| R_PGPP141pp | phosphatidylglycerol phosphate phosphatase (periplasm, n-C14:1) |
| R_PGPP160 | phosphatidylglycerol phosphate phosphatase (n-C16:0) |
| R_PGPP160pp | phosphatidylglycerol phosphate phosphatase (periplasm, n-C16:0) |
| R_PGPP161 | phosphatidylglycerol phosphate phosphatase (n-C16:1) |
| R_PGPP161pp | phosphatidylglycerol phosphate phosphatase (periplasm, n-C16:1) |
| R_PGPP180 | phosphatidylglycerol phosphate phosphatase (n-C18:0) |
| R_PGPP180pp | phosphatidylglycerol phosphate phosphatase (periplasm, n-C18:0) |
| R_PGPP181 | phosphatidylglycerol phosphate phosphatase (n-C18:1) |
| R_PGPP181pp | phosphatidylglycerol phosphate phosphatase (periplasm, n-C18:1) |
| R_PGSA120 | Phosphatidylglycerol synthase (n-C12:0) |
| R_PGSA140 | Phosphatidylglycerol synthase (n-C14:0) |
| R_PGSA141 | Phosphatidylglycerol synthase (n-C14:1) |
| R_PGSA160 | Phosphatidylglycerol synthase (n-C16:0) |
| R_PGSA161 | Phosphatidylglycerol synthase (n-C16:1) |
| R_PGSA180 | Phosphatidylglycerol synthase (n-C18:0) |
| R_PGSA181 | Phosphatidylglycerol synthase (n-C18:1) |
| R_PHEMEabcpp | protoheme transport via ABC system (periplasm) |
| R_PHEMEtiex | protoheme transport irreversible out via diffusion (periplasm to extracellular) |
| R_PHETA1 | phenylalanine transaminase |
| R_PHETRS | Phenylalanyl-tRNA synthetase |
| R_PHEt2rpp | L-phenylalanine reversible transport via proton symport (periplasm) |
| R_PHEtex | L-phenylalanine transport via diffusion (extracellular to periplasm) |
| R_PHYTSpp | Phytase (periplasm) |
| R_PIt2rpp | phosphate reversible transport via symport (periplasm) |
| R_PItex | phosphate transport via diffusion (extracellular to periplasm) |
| R_PIuabcpp | phosphate transport via ABC system (uptake, periplasm) |
| R_PLIPA1A120pp | Phospholipase A1 (phosphatidate, n-C12:0) (periplasm) |
| R_PLIPA1A140pp | Phospholipase A1 (phosphatidate, n-C14:0) (periplasm) |
| R_PLIPA1A141pp | Phospholipase A1 (phosphatidate, n-C14:1) (periplasm) |
| R_PLIPA1A160pp | Phospholipase A1 (phosphatidate, n-C16:0) (periplasm) |
| R_PLIPA1A161pp | Phospholipase A1 (phosphatidate, n-C16:1) (periplasm) |
| R_PLIPA1A180pp | Phospholipase A1 (phosphatidate, n-C18:0) (periplasm) |
| R_PLIPA1A181pp | Phospholipase A1 (phosphatidate, n-C18:1) (periplasm) |
| R_PLIPA1E120pp | Phospholipase A1 (phosphatidylethanolamine, n-C12:0) (periplasm) |
| R_PLIPA1E140pp | Phospholipase A1 (phosphatidylethanolamine, n-C14:0) (periplasm) |
| R_PLIPA1E141pp | Phospholipase A1 (phosphatidylethanolamine, n-C14:1) (periplasm) |
| R_PLIPA1E160pp | Phospholipase A1 (phosphatidylethanolamine, n-C16:0) (periplasm) |
| R_PLIPA1E161pp | Phospholipase A1 (phosphatidylethanolamine, n-C16:1) (periplasm) |
| R_PLIPA1E180pp | Phospholipase A1 (phosphatidylethanolamine, n-C18:0) (periplasm) |
| R_PLIPA1E181pp | Phospholipase A1 (phosphatidylethanolamine, n-C18:1) (periplasm) |
| R_PLIPA1G120pp | Phospholipase A1 (phosphatidylglycerol, n-C12:0) (periplasm) |
| R_PLIPA1G140pp | Phospholipase A1 (phosphatidylglycerol, n-C14:0) (periplasm) |
| R_PLIPA1G141pp | Phospholipase A1 (phosphatidylglycerol, n-C14:1) (periplasm) |
| R_PLIPA1G160pp | Phospholipase A1 (phosphatidylglycerol, n-C16:0) (periplasm) |
| R_PLIPA1G161pp | Phospholipase A1 (phosphatidylglycerol, n-C16:1) (periplasm) |
| R_PLIPA1G180pp | Phospholipase A1 (phosphatidylglycerol, n-C18:0) (periplasm) |
| R_PLIPA1G181pp | Phospholipase A1 (phosphatidylglycerol, n-C18:1) (periplasm) |
| R_PLIPA2A120pp | Phospholipase A2 (phosphatidate, n-C12:0) (periplasm) |
| R_PLIPA2A140pp | Phospholipase A2 (phosphatidate, n-C14:0) (periplasm) |
| R_PLIPA2A141pp | Phospholipase A2 (phosphatidate, n-C14:1) (periplasm) |
| R_PLIPA2A160pp | Phospholipase A2 (phosphatidate, n-C16:0) (periplasm) |
| R_PLIPA2A161pp | Phospholipase A2 (phosphatidate, n-C16:1) (periplasm) |
| R_PLIPA2A180pp | Phospholipase A2 (phosphatidate, n-C18:0) (periplasm) |
| R_PLIPA2A181pp | Phospholipase A2 (phosphatidate, n-C18:1) (periplasm) |
| R_PLIPA2E120pp | Phospholipase A2 (phosphatidylethanolamine, n-C12:0) (periplasm) |
| R_PLIPA2E140pp | Phospholipase A2 (phosphatidylethanolamine, n-C14:0) (periplasm) |
| R_PLIPA2E141pp | Phospholipase A2 (phosphatidylethanolamine, n-C14:1) (periplasm) |
| R_PLIPA2E160pp | Phospholipase A2 (phosphatidylethanolamine, n-C16:0) (periplasm) |
| R_PLIPA2E161pp | Phospholipase A2 (phosphatidylethanolamine, n-C16:1) (periplasm) |
| R_PLIPA2E180pp | Phospholipase A2 (phosphatidylethanolamine, n-C18:0) (periplasm) |
| R_PLIPA2E181pp | Phospholipase A2 (phosphatidylethanolamine, n-C18:1) (periplasm) |
| R_PLIPA2G120pp | Phospholipase A2 (phosphatidylglycerol, n-C12:0) (periplasm) |
| R_PLIPA2G140pp | Phospholipase A2 (phosphatidylglycerol, n-C14:0) (periplasm) |
| R_PLIPA2G141pp | Phospholipase A2 (phosphatidylglycerol, n-C14:1) (periplasm) |
| R_PLIPA2G160pp | Phospholipase A2 (phosphatidylglycerol, n-C16:0) (periplasm) |
| R_PLIPA2G161pp | Phospholipase A2 (phosphatidylglycerol, n-C16:1) (periplasm) |
| R_PLIPA2G180pp | Phospholipase A2 (phosphatidylglycerol, n-C18:0) (periplasm) |
| R_PLIPA2G181pp | Phospholipase A2 (phosphatidylglycerol, n-C18:1) (periplasm) |
| R_PMANM | phosphomannomutase |
| R_PMDPHT | pyrimidine phosphatase |
| R_PMEACPE | Pimeloyl-[ACP] methyl ester esterase |
| R_PMPK | phosphomethylpyrimidine kinase |
| R_PNTK | pantothenate kinase |
| R_PNTOt4pp | Pantothenate sodium symporter (periplasm) |
| R_PNTOtex | Pantothenate transport via diffusion (extracellular to periplasm) |
| R_POAACR | peroxyaminoacrylate reductase |
| R_POR5 | pyruvate synthase |
| R_POX | pyruvate oxidase |
| R_PPA | inorganic diphosphatase |
| R_PPA2 | inorganic triphosphatase |
| R_PPAKr | Propionate kinase |
| R_PPALtex | propanal transport via diffusion (extracellular to periplasm) |
| R_PPALtpp | propanal transport via channel (periplasm) |
| R_PPAt4pp | Na+/Propionate symporter (periplasm) |
| R_PPAtex | propionate transport via diffusion |
| R_PPBNGS | porphobilinogen synthase |
| R_PPC | phosphoenolpyruvate carboxylase |
| R_PPCDC | phosphopantothenoylcysteine decarboxylase |
| R_PPCK | phosphoenolpyruvate carboxykinase |
| R_PPCSCT | Propanoyl-CoA: succinate CoA-transferase |
| R_PPGPPDP | guanosine-3',5'-bis(diphosphate) 3'-diphosphatase |
| R_PPK2r | polyphosphate kinase |
| R_PPKr | polyphosphate kinase |
| R_PPM | phosphopentomutase |
| R_PPM2 | phosphopentomutase 2 (deoxyribose) |
| R_PPNCL2 | phosphopantothenate-cysteine ligase |
| R_PPND | prephenate dehydrogenase |
| R_PPNDH | prephenate dehydratase |
| R_PPPGO | protoporphyrinogen oxidase (aerobic) |
| R_PPPGO3 | protoporphyrinogen oxidase (anaerobic) |
| R_PPPNDO | Phenylpropanoate Dioxygenase |
| R_PPPNt2rpp | 3-phenylpropionate transport via proton symport, reversible (periplasm) |
| R_PPPNtex | 3-phenylpropionate transport via diffusion (extracellular to periplasm) |
| R_PPS | phosphoenolpyruvate synthase |
| R_PPTHpp | Phosphonate hydrogenase (periplasm) |
| R_PPTtex | Phosphonate transport via diffusion (extracellular to periplasm) |
| R_PRAGSr | phosphoribosylglycinamide synthase |
| R_PRAIS | phosphoribosylaminoimidazole synthase |
| R_PRAIi | phosphoribosylanthranilate isomerase (irreversible) |
| R_PRAMPC | phosphoribosyl-AMP cyclohydrolase |
| R_PRASCSi | phosphoribosylaminoimidazolesuccinocarboxamide synthase |
| R_PRATPP | phosphoribosyl-ATP pyrophosphatase |
| R_PRFGS | phosphoribosylformylglycinamidine synthase |
| R_PRMICI | 1-(5-phosphoribosyl)-5-[(5-phosphoribosylamino)methylideneamino)imidazole-4-carboxamide isomerase |
| R_PROD2 | Proline dehydrogenase |
| R_PROGLYabcpp | L-Prolinylglycine (Pro-Gly) transport via ABC system (periplasm) |
| R_PROGLYtex | L-Prolinylglycine transport via diffusion (extracellular to periplasm) |
| R_PROTRS | Prolyl-tRNA synthetase |
| R_PROabcpp | L-proline transport via ABC system (periplasm) |
| R_PROt2rpp | L-proline reversible transport via proton symport (periplasm) |
| R_PROt4pp | Na+/Proline-L symporter (periplasm) |
| R_PROtex | L-proline transport via diffusion (extracellular to periplasm) |
| R_PRPPS | phosphoribosylpyrophosphate synthetase |
| R_PSCLYSt2pp | psicoselysine transport via proton symport (periplasm) |
| R_PSCLYStex | psicoselysine transporter via diffusion (extracellular) |
| R_PSCVT | 3-phosphoshikimate 1-carboxyvinyltransferase |
| R_PSD120 | Phosphatidylserine decarboxylase (n-C12:0) |
| R_PSD140 | Phosphatidylserine decarboxylase (n-C14:0) |
| R_PSD141 | Phosphatidylserine decarboxylase (n-C14:1) |
| R_PSD160 | Phosphatidylserine decarboxylase (n-C16:0) |
| R_PSD161 | Phosphatidylserine decarboxylase (n-C16:1) |
| R_PSD180 | Phosphatidylserine decarboxylase (n-C18:0) |
| R_PSD181 | Phosphatidylserine decarboxylase (n-C18:1) |
| R_PSERT | phosphoserine transaminase |
| R_PSERtex | phospho-L-serine transport via diffusion (extracellular to periplasm) |
| R_PSP_L | phosphoserine phosphatase (L-serine) |
| R_PSP_Lpp | phospho-L-serine phosphatase (periplasmic) |
| R_PSSA120 | Phosphatidylserine syntase (n-C12:0) |
| R_PSSA140 | Phosphatidylserine syntase (n-C14:0) |
| R_PSSA141 | Phosphatidylserine syntase (n-C14:1) |
| R_PSSA160 | Phosphatidylserine syntase (n-C16:0) |
| R_PSSA161 | Phosphatidylserine syntase (n-C16:1) |
| R_PSSA180 | Phosphatidylserine syntase (n-C18:0) |
| R_PSSA181 | Phosphatidylserine syntase (n-C18:1) |
| R_PTA2 | Phosphate acetyltransferase |
| R_PTAr | phosphotransacetylase |
| R_PTHRpp | phospho-L-threonine phosphatase (periplasmic) |
| R_PTPATi | pantetheine-phosphate adenylyltransferase |
| R_PTRCORNt7pp | putrescine/ornithine antiporter (periplasm) |
| R_PTRCTA | Putrescine Transaminase |
| R_PTRCabcpp | putrescine transport via ABC system (periplasm) |
| R_PTRCt2pp | putrescine transport in via proton symport |
| R_PTRCtex | putrescine transport via diffusion (extracellular to periplasm) |
| R_PUNP1 | purine-nucleoside phosphorylase (Adenosine) |
| R_PUNP2 | purine-nucleoside phosphorylase (Deoxyadenosine) |
| R_PUNP3 | purine-nucleoside phosphorylase (Guanosine) |
| R_PUNP4 | purine-nucleoside phosphorylase (Deoxyguanosine) |
| R_PUNP5 | purine-nucleoside phosphorylase (Inosine) |
| R_PUNP6 | purine-nucleoside phosphorylase (Deoxyinosine) |
| R_PUNP7 | purine-nucleoside phosphorylase (Xanthosine) |
| R_PYAM5PO | pyridoxamine 5'-phosphate oxidase |
| R_PYDAMK | pyridoxamine kinase |
| R_PYDAMtex | pyridoxamine transport via diffusion (extracellular) |
| R_PYDAMtpp | pyridoxamine import |
| R_PYDXK | pyridoxal kinase |
| R_PYDXNK | pyridoxine kinase |
| R_PYDXNtex | pyridoxine transport via diffusion (extracellular) |
| R_PYDXNtpp | pyridoxine import |
| R_PYDXPP | Pyridoxal 5-phosphate phosphatase |
| R_PYDXtex | pyridoxal transport via diffusion (extracellular) |
| R_PYDXtpp | pyridoxal import |
| R_PYK | pyruvate kinase |
| R_PYNP2r | pyrimidine-nucleoside phosphorylase (uracil) |
| R_PYROX | pyrimidine oxygenase |
| R_PYRt2rpp | pyruvate reversible transport via proton symport (periplasm) |
| R_PYRtex | pyruvate transport via diffusion (extracellular to periplasm) |
| R_QMO2 | quinol monooxygenase (Ubiquinol-8) |
| R_QMO3 | quinol monooxygenase (menaquinol 8) |
| R_QUIN2tex | Quinate transport via diffusion (extracellular to periplasm) |
| R_QUIN2tpp | Quinate transport (periplasm) |
| R_QUINDH | Quinate dehydrogenase |
| R_QULNS | quinolinate synthase |
| R_R15BPK | ribose-1,5-bisphosphokinase |
| R_R1PK | ribose 1-phosphokinase |
| R_R5PP | ribose 5-phosphate phosphatase |
| R_R5PPpp | ribose 5-phosphate phosphatase |
| R_R5Ptex | Ribose 5-phosphate transport via diffusion (extracellular to periplasm) |
| R_RBFK | riboflavin kinase |
| R_RBFSa | riboflavin synthase |
| R_RBFSb | riboflavin synthase |
| R_RBK | ribokinase |
| R_RBK_L1 | L-ribulokinase (L-ribulose) |
| R_RBP4E | L-ribulose-phosphate 4-epimerase |
| R_REPHACCOAI | ring 1,2-epoxyphenylacetyl-CoA isomerase (oxepin-CoA forming) |
| R_RFAMPtex | Rifampin transport via diffusion (extracellular to periplasm) |
| R_RFAMPtpp | Rifampin transport via TolC system |
| R_RHAT1 | rhamnosyltransferase I (LPS core biosynthesis) |
| R_RHCCE | S-ribosylhomocysteine cleavage enzyme |
| R_RIBabcpp | D-ribose transport via ABC system (periplasm) |
| R_RIBtex | ribose transport via diffusion (extracellular to periplasm) |
| R_RMI | L-rhamnose isomerase |
| R_RMK | rhamnulokinase |
| R_RMNtex | L-rhamnose transport via diffusion (extracellular to periplasm) |
| R_RMNtpp | L-rhamnose transport via proton symport (periplasm) |
| R_RMPA | Rhamnulose-1-phosphate aldolase |
| R_RNDR1 | ribonucleoside-diphosphate reductase (ADP) |
| R_RNDR1b | ribonucleoside-diphosphate reductase (ADP) (glutaredoxin) |
| R_RNDR2 | ribonucleoside-diphosphate reductase (GDP) |
| R_RNDR2b | ribonucleoside-diphosphate reductase (GDP) (glutaredoxin) |
| R_RNDR3 | ribonucleoside-diphosphate reductase (CDP) |
| R_RNDR3b | ribonucleoside-diphosphate reductase (CDP) (glutaredoxin) |
| R_RNDR4 | ribonucleoside-diphosphate reductase (UDP) |
| R_RNDR4b | ribonucleoside-diphosphate reductase (UDP) (glutaredoxin) |
| R_RNTR1c2 | ribonucleoside-triphosphate reductase (ATP) (flavodoxin) |
| R_RNTR2c2 | ribonucleoside-triphosphate reductase (GTP) (flavodoxin) |
| R_RNTR3c2 | ribonucleoside-triphosphate reductase (CTP) (flavodoxin) |
| R_RNTR4c2 | ribonucleoside-triphosphate reductase (UTP) (flavodoxin) |
| R_RPE | ribulose 5-phosphate 3-epimerase |
| R_RPI | ribose-5-phosphate isomerase |
| R_RZ5PP | alpha-ribazole 5-phosphate phosphatase |
| R_S2FE2SR | SUF [2Fe-2S] regeneration |
| R_S2FE2SS | SUF [2Fe-2S] Synthesis |
| R_S2FE2SS2 | SUF [2Fe-2S] Synthesis II |
| R_S2FE2ST | SUF [2Fe-2S] Transfer |
| R_S4FE4SR | SUF [4Fe-4S] Reduction |
| R_S4FE4ST | SUF [4Fe-4S] Transfer |
| R_S7PI | sedoheptulose 7-phosphate isomerase |
| R_SADH | Succinylarginine dihydrolase |
| R_SADT2 | Sulfate adenyltransferase |
| R_SARCOX | sarcosine oxidase |
| R_SBTPD | sorbitol-6-phosphate dehydrogenase |
| R_SBTptspp | D-sorbitol transport via PEP:Pyr PTS (periplasm) |
| R_SBTtex | D-sorbitol transport via diffusion (extracellular to periplasm) |
| R_SCYSDS | SUF Cysteine desulfuration |
| R_SDPDS | succinyl-diaminopimelate desuccinylase |
| R_SDPTA | succinyldiaminopimelate transaminase |
| R_SELCYSS | selenocysteine synthase |
| R_SELGTHR | selenate glutathione reductase |
| R_SELGTHR2 | selenate glutathione reductase II |
| R_SELGTHR3 | selenate glutathione reductase III |
| R_SELNPS | Selenophosphate synthase |
| R_SELR | selenate reductase |
| R_SELtex | selenate transport via diffusion (extracellular to periplasm) |
| R_SELtpp | selenate transport via proton symport (periplasm) |
| R_SEPHCHCS | 2-succinyl-5-enolpyruvyl-6-hydroxy-3-cyclohexene-1-carboxylate synthase |
| R_SERASr | (L-seryl)adenylate synthase |
| R_SERAT | serine O-acetyltransferase |
| R_SERD_D | D-serine deaminase |
| R_SERD_L | L-serine deaminase |
| R_SERTRS | Seryl-tRNA synthetase |
| R_SERTRS2 | Seryl-tRNA synthetase (selenocystein) |
| R_SERt2rpp | L-serine reversible transport via proton symport (periplasm) |
| R_SERt4pp | L-serine via sodium symport (periplasm) |
| R_SERtex | L-serine transport via diffusion (extracellular to periplasm) |
| R_SFGTHi | S-Formylglutathione hydralase |
| R_SGDS | Succinylglutamate desuccinylase |
| R_SGSAD | Succinylglutamic semialdehyde dehydrogenase |
| R_SHCHCS3 | 2-succinyl-6-hydroxy-2,4-cyclohexadiene 1-carboxylate synthase |
| R_SHCHD2 | sirohydrochlorin dehydrogenase (NAD) |
| R_SHCHF | sirohydrochlorin ferrochelatase |
| R_SHK3Dr | shikimate dehydrogenase |
| R_SHKK | shikimate kinase |
| R_SHSL1 | O-succinylhomoserine lyase (L-cysteine) |
| R_SKMt2pp | shikimate transport in via proton symport (periplasm) |
| R_SKMtex | shikimate transport via diffusion (extracellular to periplasm) |
| R_SLNTtex | selenite transport via diffusion (extracellular to periplasm) |
| R_SLNTtpp | selenite transport via proton symport (periplasm) |
| R_SO2tex | SO2 transport via diffusion (extracellular to periplasm) |
| R_SO2tpp | SO2 transport via diffusion (periplasm) |
| R_SO3tex | sulfite transport via diffusion (extracellular to periplasm) |
| R_SO4t2pp | sulfate transport in via proton symport (periplasm to cytoplasm) |
| R_SO4tex | sulfate transport via diffusion (extracellular to periplasm) |
| R_SOTA | Succinylornithine transaminase |
| R_SPMDAT1 | Spermidine acetyltransferase |
| R_SPMDAT2 | Spermidine acetyltransferase (N8) |
| R_SPMDabcpp | spermidine transport via ABC system (periplasm) |
| R_SPMDt3pp | spermidine transport out via proton antiport (periplasm) |
| R_SPMDtex | spermidine transport via diffusion (extracellular to periplasm) |
| R_SPMS | spermidine synthase |
| R_SPODM | superoxide dismutase |
| R_SPODMpp | superoxide dismutase |
| R_SSALx | succinate-semialdehyde dehydrogenase (NAD) |
| R_SSALy | succinate-semialdehyde dehydrogenase (NADP) |
| R_SUCASPtpp | succinate:aspartate antiporter (periplasm) |
| R_SUCBZL | o-succinylbenzoate-CoA ligase |
| R_SUCBZS | O-succinylbenzoate-CoA synthase |
| R_SUCCt2_2pp | succinate transport via proton symport (2 H) (periplasm) |
| R_SUCCt2_3pp | Succintate transport via proton symport (3 H) (periplasm) |
| R_SUCCt3pp | succinate transport out via proton antiport (periplasm) |
| R_SUCCtex | succinate transport via diffusion (extracellular to periplasm) |
| R_SUCDi | succinate dehydrogenase (irreversible) |
| R_SUCFUMtpp | succinate:fumarate antiporter (periplasm) |
| R_SUCMALtpp | succinate:malate antiporter (periplasm) |
| R_SUCOAS | succinyl-CoA synthetase (ADP-forming) |
| R_SUCRtex | sucrose transport transport via diffusion (extracellular to periplasm) |
| R_SUCTARTtpp | succinate:D-tartrate antiporter (periplasm) |
| R_SUCptspp | sucrose transport via PEP:Pyr (periplasm) |
| R_SULFACabcpp | sulfoacetate transport via ABC system (periplasm) |
| R_SULFACtex | sulfoaceate transport via diffusion (extracellular to periplasm) |
| R_SULRi | sulfite reductase (NADPH2) |
| R_SULabcpp | sulfate transport via ABC system (periplasm) |
| R_T2DECAI | trans-2-decenoyl-ACP isomerase |
| R_TAGURr | tagaturonate reductase |
| R_TALA | transaldolase |
| R_TARTD | L(+)-tartrate dehydratase |
| R_TARTRDtex | D-tartrate transport via diffusion (extracellular to periplasm) |
| R_TARTRt7pp | Tartrate/succinate antiporter (periplasm) |
| R_TARTRtex | Tartrate transport via diffusion (extracellular to periplasm) |
| R_TARTt2_3pp | D-tartrate transport via proton symport (3 H) (periplasm) |
| R_TAUDO | Taurine dioxygenase |
| R_TAURabcpp | taurine transport via ABC system (periplasm) |
| R_TAURtex | taurine transport via diffusion (extracellular to periplasm) |
| R_TCYNTtex | Thiocyanate transport via diffusion (extracellular to periplasm) |
| R_TDP | thiamin pyrophosphatase |
| R_TDPADGAT | dTDP-4-amino-4,6-dideoxy-D-glucose acetyltransferase |
| R_TDPAGTA | dTDP-4-amino-4,6-dideoxy-D-glucose transaminase |
| R_TDPDRE | dTDP-4-dehydrorhamnose 3,5-epimerase |
| R_TDPDRR | dTDP-4-dehydrorhamnose reductase |
| R_TDPGDH | dTDPglucose 4,6-dehydratase |
| R_TDSK | Tetraacyldisaccharide 4'kinase |
| R_TDSR1 | thiol:disulfide reductase (DsbC) |
| R_TDSR2 | thiol:disulfide reductase (DsbG) |
| R_TGBPA | Tagatose-bisphosphate aldolase |
| R_THD2pp | NAD(P) transhydrogenase (periplasm) |
| R_THDPS | tetrahydrodipicolinate succinylase |
| R_THFAT | Tetrahydrofolate aminomethyltransferase |
| R_THIORDXi | hydrogen peroxide reductase (thioredoxin) |
| R_THMDt2pp | thymidine transport in via proton symport (periplasm) |
| R_THMDt2rpp | thymidine transport in via proton symport, reversible (periplasm) |
| R_THMDtex | thymidine transport via diffusion (extracellular to periplasm) |
| R_THMabcpp | thiamine transport via ABC system (periplasm) |
| R_THMtex | Thiamine transport via diffusion (extracellular to periplasm) |
| R_THRA2i | L-allo-Threonine Aldolase |
| R_THRAi | Threonine aldolase |
| R_THRD | L-threonine dehydrogenase |
| R_THRD_L | L-threonine deaminase |
| R_THRPtex | phospho-L-threonine transport via diffusion (extracellular to periplasm) |
| R_THRS | threonine synthase |
| R_THRTRS | Threonyl-tRNA synthetase |
| R_THRabcpp | L-threonine transport via ABC system (periplasm) |
| R_THRt2pp | L-threonine efflux transport via proton antiport (periplasm) |
| R_THRt2rpp | L-threonine reversible transport via proton symport (periplasm) |
| R_THRt4pp | L-threonine via sodium symport (periplasm) |
| R_THRtex | L-threonine transport via diffusion (extracellular to periplasm) |
| R_THYMt3pp | thymine transport out via proton antiport (periplasm) |
| R_THYMtex | thymine transport via diffusion (extracellular to periplasm) |
| R_THZPSN3 | thiazole phosphate synthesis |
| R_TKT1 | transketolase |
| R_TKT2 | transketolase |
| R_TMAOR1 | Trimethylamine N-oxide reductase (menaquinol 8) |
| R_TMAOR1pp | Trimethylamine N-oxide reductase (menaquinol 8) (periplasm) |
| R_TMAOR2 | Trimethylamine N-oxide reductase (demethylmenaquinol 8) |
| R_TMAOR2pp | Trimethylamine N-oxide reductase (demethylmenaquinol 8) (periplasm) |
| R_TMAOtex | Trimethylamine N-oxide transport via diffusion (extracellular to periplasm) |
| R_TMAtex | Trimethylamine transport via diffusion (extracellular to periplasm) |
| R_TMDK1 | thymidine kinase (ATP:thymidine) |
| R_TMDPP | thymidine phosphorylase |
| R_TMDS | thymidylate synthase |
| R_TMK | thiamine kinase |
| R_TMPK | thiamine-phosphate kinase |
| R_TMPPP | thiamine-phosphate diphosphorylase |
| R_TPI | triose-phosphate isomerase |
| R_TPRDCOAS | triphosphoribosyl-dephospho-CoA synthase |
| R_TRDR | thioredoxin reductase (NADPH) |
| R_TRE6PH | trehalose-6-phosphate hydrolase |
| R_TRE6PP | trehalose-phosphatase |
| R_TRE6PS | alpha,alpha-trehalose-phosphate synthase (UDP-forming) |
| R_TREH | alpha,alpha-trehalase |
| R_TREHpp | alpha,alpha-trehalase (periplasm) |
| R_TREptspp | trehalose transport via PEP:Pyr PTS (periplasm) |
| R_TREtex | trehalose transport via diffusion (extracellular to periplasm) |
| R_TRPAS2 | Tryptophanase (L-tryptophan) |
| R_TRPS1 | tryptophan synthase (indoleglycerol phosphate) |
| R_TRPS2 | tryptophan synthase (indole) |
| R_TRPS3 | tryptophan synthase (indoleglycerol phosphate) |
| R_TRPTRS | Tryptophanyl-tRNA synthetase |
| R_TRPt2rpp | L-tryptophan reversible transport via proton symport (periplasm) |
| R_TRPtex | L-tryptophan transport via diffusion (extracellular to periplasm) |
| R_TRSARr | tartronate semialdehyde reductase |
| R_TSULabcpp | thiosulfate transport via ABC system (periplasm) |
| R_TSULtex | thiosulfate transport via diffusion (extracellular to periplasm) |
| R_TTDCAtexi | Tetradecanoate transport via facilitated irreversible diffusion (extracellular to periplasm) |
| R_TTDCEAtexi | Tetradecenoate transport via facilitated irreversible diffusion (extracellular to periplasm) |
| R_TTRCYCtex | Tetracycline transport via diffusion (extracellular to periplasm) |
| R_TTRCYCtpp | Tetracycline transport via TolC system |
| R_TUNGSabcpp | tungstate transport via ABC system (periplasm) |
| R_TUNGStex | tungstate transport via diffusion (extracellular to periplasm) |
| R_TYMtex | tyramine transport via diffusion (extracellular to periplasm) |
| R_TYRL | tyrosine lyase |
| R_TYROXDApp | Tyramine:oxygen oxidoreductase(deaminating)(flavin-containing) (periplasm) |
| R_TYRPpp | phospho-L-tyrosine phosphatase (periplasmic) |
| R_TYRPtex | phopho-L-tyrosine transport via diffusion (extracellular to periplasm) |
| R_TYRTA | tyrosine transaminase |
| R_TYRTRS | tyrosyl-tRNA synthetase |
| R_TYRt2rpp | L-tyrosine reversible transport via proton symport (periplasm) |
| R_TYRtex | L-tyrosine transport via diffusion (extracellular to periplasm) |
| R_ThDPAT | ThDP adenylyl transferase |
| R_U23GAAT | UDP-3-O-(3-hydroxymyristoyl)glucosamine acyltransferase |
| R_UAAGDS | UDP-N-acetylmuramoyl-L-alanyl-D-glutamyl-meso-2,6-diaminopimelate synthetase |
| R_UACGALPpp | UDP-N-acetyl-D-galactosamine pyrophosphohydrolase (periplasm) |
| R_UACGAMPpp | UDP-N-acetyl-D-glucosamine pyrophosphohydrolase (periplasm) |
| R_UACGAMtex | UDP-N-acetyl-D-glucosamine transport via diffusion (extracellular to periplasm) |
| R_UACMAMO | UDP-N-acetyl-D-mannosamine oxidoreductase |
| R_UAG2E | UDP-N-acetylglucosamine 2-epimerase |
| R_UAGAAT | UDP-N-acetylglucosamine acyltransferase |
| R_UAGCVT | UDP-N-acetylglucosamine 1-carboxyvinyltransferase |
| R_UAGDP | UDP-N-acetylglucosamine diphosphorylase |
| R_UAGPT3 | UDP-N-acetylglucosamine-N-acetylmuramyl-(pentapeptide)pyrophosphoryl-undecaprenol N-acetylglucosamine transferase |
| R_UAMAGS | UDP-N-acetylmuramoyl-L-alanyl-D-glutamate synthetase |
| R_UAMAS | UDP-N-acetylmuramoyl-L-alanine synthetase |
| R_UAPGR | UDP-N-acetylenolpyruvoylglucosamine reductase |
| R_UDCPDP | undecaprenyl-diphosphatase |
| R_UDCPDPS | Undecaprenyl diphosphate synthase |
| R_UDCPDPpp | undecaprenyl-diphosphatase (periplasm) |
| R_UDCPPtppi | undecaprenyl phosphate transport (cytoplasm to periplasm) |
| R_UDPACGALtex | UDP-N-acetyl-D-galactosamine transport via diffusion (extracellular to periplasm) |
| R_UDPG4E | UDPglucose 4-epimerase |
| R_UDPGALM | UDPgalactopyranose mutase |
| R_UDPGALPpp | UDPgalactose pyrophosphohydrolase |
| R_UDPGALtex | UDPgalactose transport via diffusion (extracellular to periplasm) |
| R_UDPGD | UDPglucose 6-dehydrogenase |
| R_UDPGDC | UDP-glucuronate C-4'' decarboxylase |
| R_UDPGLCURtex | UDP-D-glucuronate transport via diffusion (extracellular to periplasm) |
| R_UDPGPpp | UDPglucose pyrophosphohydrolase |
| R_UDPGtex | UDPglucose transport via diffusion (extracellular to periplasm) |
| R_UDPKAAT | UDP-4''-ketopentose:UDP-4-amino-4-deoxy-L-arabinose aminotransferase |
| R_UGLCURPpp | UDP-D-glucuronate pyrophosphohydrolase (periplasm) |
| R_UGLT | UDPglucose--hexose-1-phosphate uridylyltransferase |
| R_UGLYCH | Ureidoglycolate hydrolase |
| R_UGMDDS | UDP-N-acetylmuramoyl-L-alanyl-D-glutamyl-meso-2,6-diaminopimeloyl-D-alanyl-D-alanine synthetase |
| R_UHGADA | UDP-3-O-acetylglucosamine deacetylase |
| R_ULA4NFT | UDP-L-Ara4N formyltransferase |
| R_ULA4Ntppi | transport (cytoplasm to periplasm) |
| R_UM3PL | UDP-N-acetylmuramate:L-alanyl-gamma-D-glutamyl-meso-diaminopimelate ligase |
| R_UM4PCP | UDP-N-acetylmuramoyl-L-alanyl-D-gamma-glutamyl-meso-2,6-diaminopimelate-D-alanine L,D-carboxypeptidase |
| R_UM4PL | UDP-N-acetylmuramate:L-alanyl-gamma-D-glutamyl-meso-diaminopimelate-D-alanine ligase |
| R_UMPK | UMP kinase |
| R_UMPtex | UMP transport via diffusion (extracellular to periplasm) |
| R_UPLA4FNF | undecaprenyl phosphate-L-Ara4FN formylase |
| R_UPLA4FNT | undecaprenyl phosphate-L-Ara4FN transferase |
| R_UPP3MT | uroporphyrinogen methyltransferase |
| R_UPP3S | uroporphyrinogen-III synthase |
| R_UPPDC1 | uroporphyrinogen decarboxylase (uroporphyrinogen III) |
| R_UPPRT | uracil phosphoribosyltransferase |
| R_URACPAH | peroxyureidoacrylate hydrolase |
| R_URAt2pp | uracil transport in via proton symport (periplasm) |
| R_URAt2rpp | uracil transport in via proton symport, reversible (periplasm) |
| R_URAtex | uracil transport via diffusion (extracellular to periplasm) |
| R_URDGLYCD | ureidoglycolate dehydrogenase |
| R_UREAtex | Urea transport via diffusion (extracellular to periplasm) |
| R_UREAtpp | Urea transport via facilitate diffusion (periplasm) |
| R_URIC | uricase |
| R_URIH | Uridine hydrolase |
| R_URIK2 | uridine kinase (GTP:Uridine) |
| R_URIt2pp | uridine transport in via proton symport (periplasm) |
| R_URIt2rpp | uridine transport in via proton symport, reversible (periplasm) |
| R_URItex | uridine transport via diffusion (extracellular to periplasm) |
| R_USHD | UDP-sugar hydrolase |
| R_VALTA | valine transaminase |
| R_VALTRS | Valyl-tRNA synthetase |
| R_VALabcpp | L-valine transport via ABC system (periplasm) |
| R_VALt2rpp | L-valine reversible transport via proton symport (periplasm) |
| R_VALtex | L-valine transport via diffusion (extracellular to periplasm) |
| R_VPAMTr | Valine-pyruvate aminotransferase |
| R_WCOS | tungsten pterin cofactor synthase |
| R_X5PL3E | L-xylulose 5-phosphate 3-epimerase |
| R_XAND | xanthine dehydrogenase |
| R_XANt2pp | xanthine transport in via proton symport (periplasm) |
| R_XANtex | xanthine transport via diffusion (extracellular to periplasm) |
| R_XANtpp | xanthine reversible transport (periplasm) |
| R_XMPtex | XMP transport via diffusion (extracellular to periplasm) |
| R_XPPT | xanthine phosphoribosyltransferase |
| R_XTSNH | Xanthosine hydrolase |
| R_XTSNt2rpp | Xanthosine transport via proton symport (periplasm) |
| R_XTSNtex | xanthosine transport via diffusion (extracellular to periplasm) |
| R_XYLI1 | xylose isomerase |
| R_XYLI2 | xylose isomerase |
| R_XYLK | xylulokinase |
| R_XYLK2 | L-xylulokinase |
| R_XYLUt2pp | L-xylulose transport in via proton symport (periplasm) |
| R_XYLUtex | L-xylulose transport via diffusion (extracellular to periplasm) |
| R_XYLabcpp | D-xylose transport via ABC system (periplasm) |
| R_XYLt2pp | D-xylose transport in via proton symport (periplasm) |
| R_XYLtex | D-xylose transport via diffusion (extracellular to periplasm) |
| R_ZN2abcpp | Zinc (Zn+2) ABC transporter, efflux (periplasm) |
| R_ZN2t3pp | zinc (Zn+2) transport out via proton antiport (periplasm) |
| R_ZN2tpp | zinc transport in via permease (no H+) |
| R_ZNabcpp | zinc (Zn+2) transport via ABC system (periplasm) |
| R_Zn2tex | zinc (Zn+2) transport via diffusion (extracellular to periplasm) |
